# The PLEKHA1-TACC2 fusion gene drives tumorigenesis via vascular mimicry formation in esophageal squamous-cell carcinoma

**DOI:** 10.1038/s41418-025-01536-1

**Published:** 2025-07-05

**Authors:** Ting Yang, Zhi-Rui Lin, Tian-Liang Xia, Shang-Xin Liu, Bo-Yu Yuan, Yi-Ling Luo, Wen-Ting Du, Chao-Bo Lei, Yong-Zhan Nie, Mu-Sheng Zeng, Qian Zhong

**Affiliations:** 1 State Key Laboratory of Oncology in South China, Guangdong Provincial Clinical Research Center for Cancer, Sun Yat-sen University Cancer Center, Guangzhou, China; 2 Institute of Medical Research, Guangdong Provincial People’s Hospital (Guangdong Academy of Medical Sciences), Southern Medical University, Guangzhou, China; 3 Department of Biochemistry, Zhongshan School of Medicine, Sun Yat-sen University, Guangzhou, China; 4 State Key Laboratory of Cancer Biology, National Clinical Research Center for Digestive Diseases and Xijing Hospital of Digestive Diseases, The Fourth Military Medical University, Xi’an, Shaanxi China

**Keywords:** Cancer genetics, Tumour biomarkers

## Abstract

Despite advancements of diagnosis and multimodality therapies in esophageal squamous-cell carcinoma (ESCC), the survival is still unsatisfactory. Therefore, it is urgent to identify novel targets for efficient therapeutic strategies. Herein, we identify a fusion gene between *PLEKHA1* and *TACC2* generated by chromosomal rearrangement by performing RNA sequencing from ESCC tissues. *PLEKHA1-TACC2* transcripts are present in ESCC (66/404, 16.3%) and head and neck squamous cell carcinoma (58/402, 14.4%) tissues, correlated with poor prognosis of patients. Mechanistically, the fusion proteins upregulate the EphA2/AKT/MMP2 signaling pathway and promote vascular mimicry formation by reducing the ubiquitylation of EphA2. Moreover, EphA2 inhibitors dasatinib and ALW II-41-27 remarkably suppress the progression of tumors expressing PLEKHA1-TACC2 in vivo. Functionally, PLEKHA1-TACC2 fusion and Trp53 deletion significantly increases tumor incidence, tumor multiplicity, and mouse mortality in transgenic ESCC mouse model, which could be suppressed by regorafenib, a EphA2 inhibitor approved by FDA in solid tumors. Together, our data indicate that PLEKHA1-TACC2 fusion protein has oncogenic activities and serves as a promising prognosis marker and therapeutic target.

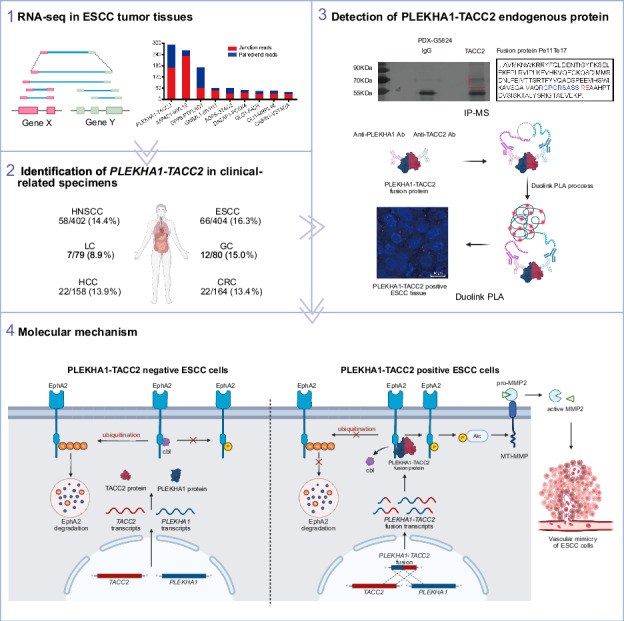

## Introduction

Esophageal carcinoma is one of the leading causes of cancer-related mortality worldwide. Esophageal squamous-cell carcinoma (ESCC), the predominant histological subtype of esophageal carcinoma, accounts for approximately 90% of newly diagnosed esophageal cancers in Asian countries [1]. For locally advanced ESCC, esophagectomy is the current primary treatment approach. Although chemotherapy and radiotherapy are commonly used for treating metastatic and recurrent ESCC, the 5-year survival rates are dismal. This is mainly because of the propensity of ESCC to extensively disseminate and its low sensitivity to chemotherapy or targeted therapy [2, 3]. The carcinogenesis of ESCC is generally a multistep process, reflecting cumulative chromosomal and genetic alterations. Currently, there are no molecule-targeted agents specific for treating ESCC. The identification of early diagnostic tumor markers and effective therapeutic targets is essential to significantly improve the clinical management of ESCC.

Chromosomal rearrangement and chromosomal translocations are the main causes of fusion genes, which can result in cancer as well as other diseases [4]. Gene fusion brings unrelated genes together, which can form new functions or enhance the function of one of the fusion partner to enhance cellular proliferation and survival [5, 6]. Fusion genes have been found to play important roles in various tumors, such as leukemia [7], glioma [8], non-small cell lung cancer [9], cholangiocarcinoma [10], and nasopharyngeal carcinoma [11]. Many therapeutics targeting fusion proteins encoded by these fusion genes are now standard of care. Tyrosine kinase inhibitors (TKIs) such as imatinib, dasatinib, and nilotinib have markedly improved outcomes of patients with chronic myeloid leukemia, the majority of which is caused by the classical oncogenic fusion gene BCR-ABL [12, 13]. ALK TKIs have been shown to be highly effective in lung adenocarcinomas with ALK-fusions [14], leading to dramatically improved survival rate [15]. Selprcatinib has demonstrated marked efficacy in patients with locally advanced or metastatic solid tumors with RET gene fusion [16], and has been approved by FDA for the treatment of such patients [17]. Therefore, it is crucial to identify novel fusion genes for developing new targeted therapeutic strategies of ESCC.

Recent research has suggested that ESCC cells could form vascular mimicry (VM), which was strongly correlated with poor outcomes in patients [18]. VM is a novel tumor microcirculation model in the tumor microenvironment, depending on tumor cells rather than endothelial cells, which is characterized by tubular structures formed by tumor cells expressing endothelial markers and can provide adequate blood supply for tumor progression [19]. At present, some molecular mechanisms of VM have been reported, such as cancer stem cells [20], epithelial-mesenchymal transition [21], and various signaling pathways [22, 23]. However, there are few studies to elucidate the molecular mechanisms of VM formation in ESCC. Tang et al. reported hypoxia-inducible factor-1 (HIF-1) might play a crucial role in VM formation and the key signaling pathway needs to be further explored [24]. Many inhibitors have been developed to restrain tumor VM specifically via targeting some signaling pathways involving factors. For instance, Galunisertib that is currently under clinical trials, was reported to inhibit VM formation by downregulating the expression of transforming growth factor-beta (TGF-β) pathway in glioma [25], while the therapeutic strategies via impairing VM formation remain to be elucidated in ESCC. The approaches for targeting VM have attracted considerable interest in ESCC treatments.

Herein, we report the identification of the *PLEKHA1-TACC2* (PT) fusion gene generated by chromosomal rearrangement in ESCC using RNA sequencing and long-range PCR. Pleckstrin homology domain containing family A member 1 (PLEKHA1) has been demonstrated to specifically bind phosphatidylinositol 3,4-bisphosphate to regulate the formation of signalling complexes in the plasma membrane. Polymorphisms of PLEKHA1 are associated with various diseases, including age-related macular degeneration and type 2 diabetes, while there was no study describing the role of PLEKHA1 in tumors. Transforming acidic coiled-coil protein 2 (TACC2) is critical in stabilizing mitosis spindles and chromosome segregation process [26, 27]. Furthermore, we found that the PLEKHA1-TACC2 fusion proteins could significantly promote tumor growth by inducing VM formation, which is caused by stabilizing EphA2 protein and upregulating the EphA2/AKT/MMP2 signaling pathway. EphA2 inhibitors can significantly inhibit PT-positive tumor growth, indicating that PT plays an important role in the development of PT-positive ESCC, and suppression of EphA2 could be a potent therapeutic strategy for patients with PT-positive ESCC.

## Results

### Identification of the *PLEKHA1-TACC2* fusion gene in ESCC

**Fig. 1:**
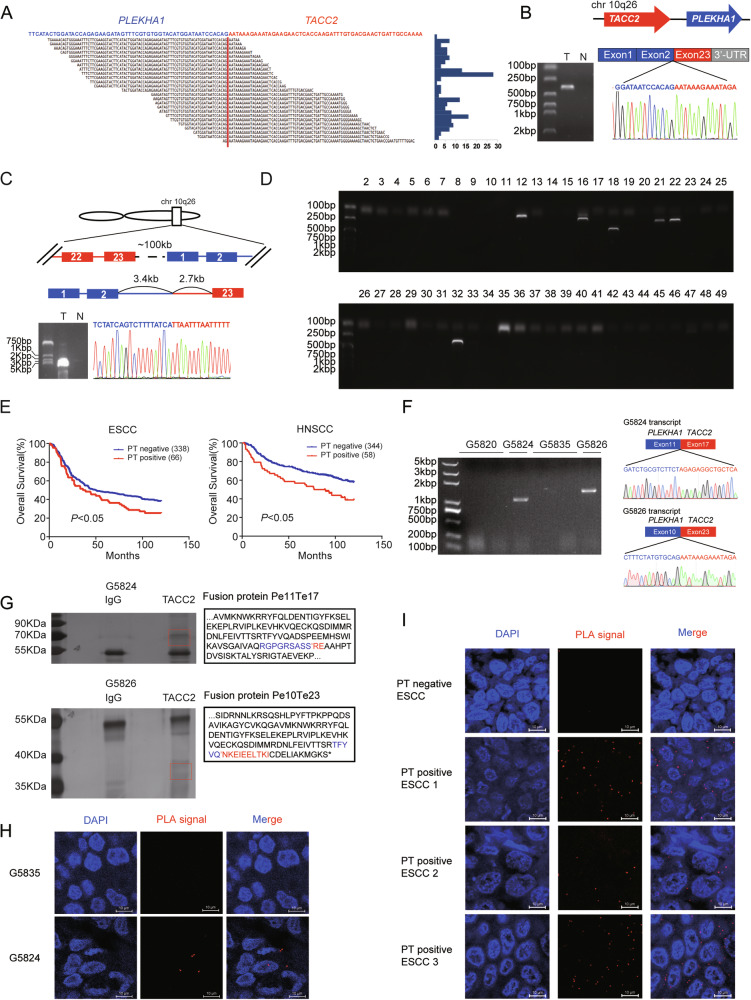
Identification of *PLEKHA1-TACC2* fusion gene. **A** Junction reads for the *PLEKHA1-TACC2* fusion transcript. **B**, **C** Detection of transcriptional (**B**) and genomic (**C**) fusion sties of *PLEKHA1-TACC2* fusion in tumor tissue (T) and normal tissue (N) from ESCC 1. The sequencing results (bottom) and scheme (top) of the PCR product of *PLEKHA1-TACC2* are shown. UTR, untranslated region. **D** Detection of *PLEKHA1-TACC2* transcripts in specimens obtained from ESCC patients by RT–PCR. **E** Kaplan-Meier analysis of the OS in patients with ESCC (left) or HNSCC (right) grouped according to the presence or absence of the expression of *PLEKHA1-TACC2* fusion. The *P* values were calculated using the log-rank test. **F** Detection of *PLEKHA1-TACC2* transcripts in tumor tissues from PDX G5820, G5824, G5835, and G5826 by RT-PCR. The sequencing results (bottom) and scheme (top) of the PCR products of *PLEKHA1-TACC2* are shown. **G** SDS/PAGE and silver staining analysis of proteins pulled down using C-terminal TACC2 antibody or IgG antibody (left) and the predicted endogenous PLEKHA1-TACC2 protein sequences (right) in tumor tissues from PDX G5824 (top) and G5826 (bottom). The predicted fusion peptides were identified via mass spectrometry peptide sequencing. The black indicates the part of predicated fusion protein sequencing. The blue indicates the amino acids from the protein sequence of PLEKHA1, and the red indicates the amino acids from the protein sequence of TACC2. **H** Detection of the endogenous PLEKHA1-TACC2 protein by Duolink PLA assay in specimens obtained from the indicated PDX. Red: positive Duolink PLA fluorescence signals, blue: nuclei. Scale bars: 10 μm. **I** Detection of the endogenous PLEKHA1-TACC2 protein by Duolink PLA assay in ESCC specimens obtained from patients. Red: positive Duolink PLA fluorescence signals, blue: nuclei. Scale bars: 10 μm.

To identify oncogenic fusion genes in ESCC, we collected 6 ESCC samples to perform RNA-seq. Based on RNA-seq reads that spanned exon-exon junctions originated from 2 distinct genes (Fig. S1A), we identified a fusion transcript of *PLEKHA1* and *TACC2* on chromosome 10q26 in one ESCC (ESCC 1), with the relatively high number of reads among the fusion transcripts (Figs. S1B, 1A). The full-length transcript (NM_021622.5) of *PLEKHA1* harboring 12 exons encodes a pleckstrin homology (PH) domain-containing adapter protein localizing to plasma membrane, and the full-length transcript (NM_206862.4) of *TACC2* harboring 23 exons encodes centrosomes/microtubules interaction associated proteins containing a highly conserved C-terminal coiled-coil (TACC domain). We performed RT–PCR using primers for the exon 2 of *PLEKHA1* and the 3’- untranslated regions (UTR) of *TACC2* to detect the predicated *PLEKHA1-TACC2* transcript in tumor tissue of ESCC 1 (Fig. 1B), followed by Sanger sequencing. The result showed that this fusion transcript included exon 1 and 2 of *PLEKHA1* and exon 23 of *TACC2*. To explore whether the *PLEKHA1-TACC2* fusion resulted from a genomic DNA rearrangement, we performed long-range PCR with a pair of primers specific to the fusion-gene and genomic DNA of tumor tissue of ESCC 1 and obtained a PCR product longer than 5 kb. The subsequent Sanger sequencing of the PCR product revealed that *PLEKHA1* was disrupted at 3.4 kb downstream of exon 2 and was connected 2.7 kb upstream of exon 23 of *TACC2* (Fig. 1C). These results confirm that the *PLEKHA1-TACC2* fusion gene resulted from a genomic rearrangement.

### Detection of *PLEKHA1-TACC2* transcripts in clinical specimens

To further determine the prevalence of the *PLEKHA1-TACC2* fusion in ESCC, we examined 404 tumor samples from ESCC patients using RT-PCR as described above and found the existence of *PLEKHA1-TACC2* in 66 ESCC samples (16.3%) (Fig. 1D). According to the cancer cell Lines Gene fusions portal (LiGeA), a comprehensive database of human gene fusion events, the *PLEKHA1-TACC2* fusion gene was detected in GSU, NCIH1184 and NCIH1435 with different transcript forms (Table 1). Therefore, to explore whether the fusion gene was also present in other tumors, we detected *PLEKHA1-TACC2* by RT-PCR, combined with Sanger sequencing, in different types of tumors. The *PLEKHA1-TACC2* fusion gene was found in 58/402 head and neck squamous cell carcinoma (HNSCC) biopsies (14.4%), 22/164 colorectal cancer (CRC) biopsies (13.4%), 22/158 hepatocellular carcinoma (HCC) biopsies (13.9%), 7/79 lung cancer (LC) biopsies (8.9%) and 12/80 gastric cancer (GC) biopsies (15%), but not in breast cancer (Table 2). We detected a total of 28 different *PLEKHA1-TACC2* fusion transcripts, among which 17/28 transcripts were in-frame fusion, while others could just encode truncated PLEKHA1 protein without the TACC2 C-terminus because of a frame-shift mutation. The frequent isoforms of PT fusion gene include a fusion between exon 2 of *PLEKHA1* and exon 23 of *TACC2* (Pe2Te23), a fusion between exon 11 of *PLEKHA1* and exon 17 of *TACC2* (Pe11Te17), a fusion between exon 10 of *PLEKHA1* and exon 23 of *TACC2* (Pe10Te23), a fusion between exon 5 of *PLEKHA1* and exon 17 of *TACC2* (Pe5Te17), and a fusion between exon 4 of *PLEKHA1* and exon 20 of *TACC2* (Pe4Te20) (Fig. S1C). To investigate the clinical importance of *PLEKHA1-TACC2*, we collected the clinical characteristics of ESCC and HNSCC patients and divided the patients into two groups based on *PLEKHA1-TACC2* expression (Tables S1–2). Multivariate Cox regression analyses confirmed that *PLEKHA1-TACC2* expression was significantly associated with unfavorable overall survival (OS) (Fig. S2). And Kaplan–Meier analysis was performed and found that the presence of *PLEKHA1-TACC2* expression in ESCC and HNSCC was significantly associated with poorer OS of patients (Fig. 1E).

**Table 1:** The transcripts of *PLEKHA1-TACC2* in cell lines.

Cell name	Lineage	Fusion type
GSU	Esophagus/stomach	Pe2Te7, Pe2Te8, Pe1Te8
NCIH1184	Lung	Pe6Te8
NCIH1435	Lung	Pe1Te20

**Table 2:** Frequency and the number of fusion types of *PLEKHA1-TACC2* identified in different types of cancers.

Cancer type	Total samples	Positive samples	Frequency
ESCC	404	66	16.3%
HNSCC	402	58	14.4%
CRC	164	22	13.4%
HCC	158	22	13.9%
LC	79	7	8.9%
GC	80	12	15%
BC	132	0	0%

### Detection of PLEKHA1-TACC2 proteins in clinical-related specimens

To examine whether these in-frame fusion PT transcripts could encode proteins, we screened several established patient-derived xenograft (PDX) models of gastric cancer for *PLEKHA1-TACC2* fusion transcripts by RT-PCR. We detected a fusion transcript of *PLEKHA1* exon 11 to *TACC2* exon 17 in G5824 and a fusion transcript *PLEKHA1* exon 10 to *TACC2* exon 23 in G5826, but we did not detect any PT transcript in G5820 or G5835 (Fig. 1F). According to the sequence of Pe11Te17 and Pe10Te23 transcripts, the predicted fusion protein contained major parts of the PLEKHA1 protein and part of the TACC2 conserved C-terminal transforming acidic coiled-coil domain. To validate the presence of predicted fusion proteins, we performed immunoprecipitation using an anti-TACC2 C-terminal antibody and lysates from G5824 and G5826 tumor tissues. As shown in Fig. 1G, several specific TACC2- interacted protein bands were visualized by sliver staining and isolated. The subsequent mass spectrometry analysis showed that N-terminal peptides of PLEKHA1 and C-terminal peptides of TACC2 were identified. It was worth to note that we detected the peptide RGPGRSASSRE containing the fusion site of predicted Pe11Te17 protein and the peptide TFYVQNKEIEELTKI containing the fusion site of predicted Pe10Te23 protein. Additionally, we performed Duolink in situ proximity ligation assay (PLA), which can detect interactions among different endogenous proteins in fixed tissue, to further identify the fusion protein. We observed strong positive fluorescence signals in the PDX G5824, but almost no signal in the PDX G5835 (Fig. 1H), further convincing the occurrence of endogenous PLEKHA1-TACC2 protein in human tumor tissues. In addition, we used 31 ESCC tissues harboring PT fusion mRNA that was predicated to encode protein to detect endogenous protein expression. Strong positive fluorescence signals were detected in 16/31 ESCC cancer tissues but not in the 3 tissues without PT fusion mRNA (Fig. 1I). Additionally, Duolink PLA in 3 ESCC tissues with PT expression and the paired adjacent normal tissues further confirmed endogenous PLEKHA1-TACC2 fusion proteins were specifically expressed in tumor tissues (Fig. S3). Our results suggest that PT transcripts could encode endogenous fusion proteins in tumor tissues from ESCC patients.

### PLEKHA1-TACC2 activated EphA2/AKT/MMP2 signalling pathway

**Fig. 2:**
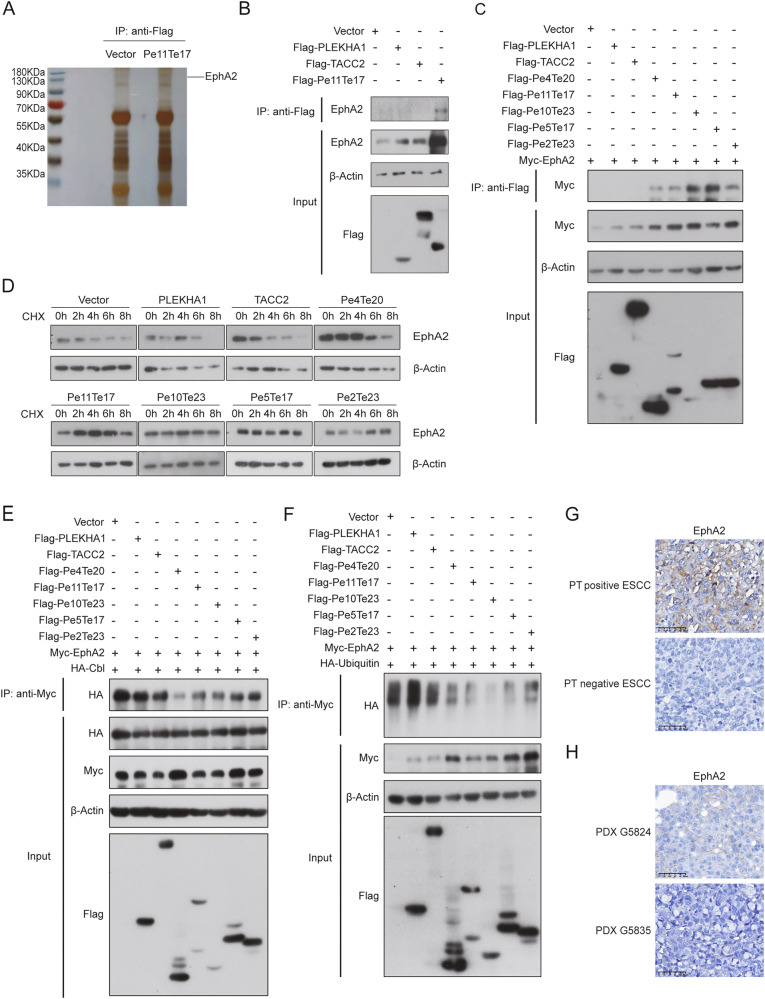
PLEKHA1-TACC2 stabilizes EphA2 by inhibiting EphA2 ubiquitination. **A** SDS/PAGE and silver staining analysis of proteins affinity purified by using anti-Flag beads from lysates of cells overexpressing Flag (vector) or Flag-Pe11Te17. EphA2 was one of the major interacting proteins identified by mass spectrum. **B** Co-immunoprecipitation assay using KYSE30 cell lysates with anti-Flag followed by immunoblotting with antibodies against the indicated proteins. **C** Results of co-immunoprecipitation assays and immunoblotting analyses of KYSE30 cells stably expressing Flag-tagged PLEKHA1, TACC2, Pe4Te20, Pe11Te17, Pe10Te23, Pe5Te17, Pe2Te23, or an empty vector and transfected with Myc-tagged EphA2 using the indicated antibodies. **D** The expression of EphA2 in KYSE30 cells stably expressing Flag-tagged PLEKHA1, TACC2, Pe4Te20, Pe11Te17, Pe10Te23, Pe5Te17, Pe2Te23, or an empty vector treated with CHX for indicated duration was determined by immunoblotting analyses. **E** KYSE30 cells stably expressing Flag-tagged PLEKHA1, TACC2, Pe4Te20, Pe11Te17, Pe10Te23, Pe5Te17, Pe2Te23, or an empty vector were transfected with Myc-tagged EphA2 and HA-cbl, and co-immunoprecipitation assays and immunoblotting analyses with the indicated antibodies were performed. **F** KYSE30 cells stably expressing Flag-tagged PLEKHA1, TACC2, Pe4Te20, Pe11Te17, Pe10Te23, Pe5Te17, Pe2Te23, or an empty vector were transfected with Myc-tagged EphA2 and HA-ubiquitin, and co-immunoprecipitation assays and immunoblotting analyses with the indicated antibodies were performed. **G**, **H** The expression of EphA2 in tumor tissues obtained from the ESCC patients (**G**) and PDX grown in mice (**H**). Scale bars were indicated in the images.

After confirming the expression of PT in tumor tissues, we investigated the role and mechanism of PLEKHA1-TACC2 in ESCC. We generated KYSE30 and KYSE150 cell lines stably expressing Flag-tagged PLEKHA1, TACC2, different PT fusion isoforms (Pe4Te20, Pe11Te17, Pe10Te23, Pe5Te17, and Pe2Te23) or an empty vector via lentivirus infection. Since Pe11Te17 is one of the most frequent PT fusions and its encoded protein is detected in PDX G5824, we applied co-immunoprecipitation in combination with mass spectrometry analysis to identify protein(s) interacting with PLEKHA1-TACC2. EphA2 was identified as an interacting protein with Pe11Te17 (Fig. 2A). Co-immunoprecipitation experiments showed that the endogenous EphA2 interacted with PLEKHA1-TACC2 but did not interact significantly with PLEKHA1 or TACC2 (Fig. 2B). Moreover, the interaction between other PT isoforms (Pe4Te20, Pe11Te17, Pe10Te23, Pe5Te17, and Pe2Te23) and EphA2 were also detected (Fig. 2C). Considering that all the five PT fusion isoforms harbor the N-terminal PH (PH1) domain (aa 7-112) of PLEKHA1, we performed co-IP assay to further confirm the interaction between PH1 domain and EphA2 (Fig. S4A). The results demonstrated that PH1 domain could interact efficiently with EphA2, suggesting that PT fusions might activate EphA2/AKT/MMP2 axis via interaction between PH1 domain and EphA2. To investigate whether PT affects the stability of EphA2, we measured the EphA2 protein level in cells treated with cycloheximide (CHX) for different durations and found that overexpression of PLEKHA1-TACC2 inhibited the degradation of EphA2 in both KYSE30 and KYSE150 cells (Figs. 2D, S4B).

EphA2 is a receptor tyrosine kinase involved in cell proliferation, migration, and differentiation [28]. C-cbl is a well-characterized E3 ubiquitin ligase that mediates ubiquitination of receptor tyrosine kinases, and c-cbl-mediated ubiquitination represents a key mechanism driving EphA2 proteasome degradation [29]. Therefore, we speculated that PT might interfere with the interaction of EphA2 and c-cbl and thus inhibit EphA2 degradation. Co-immunoprecipitation experiments confirmed that overexpression of different PLEKHA1-TACC2 isoforms significantly restrained the interaction between EphA2 and c-cbl to different extents (Figs. 2E, S4C). In addition, we demonstrated that overexpression of PLEKHA1-TACC2 significantly reduced the level of EphA2 ubiquitination in both KYSE30 and KYSE150 cells (Figs. 2F, S4D). Moreover, IHC staining confirmed that the level of EphA2 was increased in PT positive ESCC clinical samples and in the tumors of PDX G5824 compared to that in PT negative ESCC clinical samples and in the tumors of PDX G5835, confirming the finding that PLEKHA1-TACC2 promoted EphA2 protein expression in vivo (Fig. 2G, H). Taken together, these results showed that PLEKHA1-TACC2 increased the protein level of EphA2 by interfering with the interaction between EphA2 and c-cbl, reducing the level of EphA2 ubiquitination, and stabilizing EphA2.

**Fig. 3:**
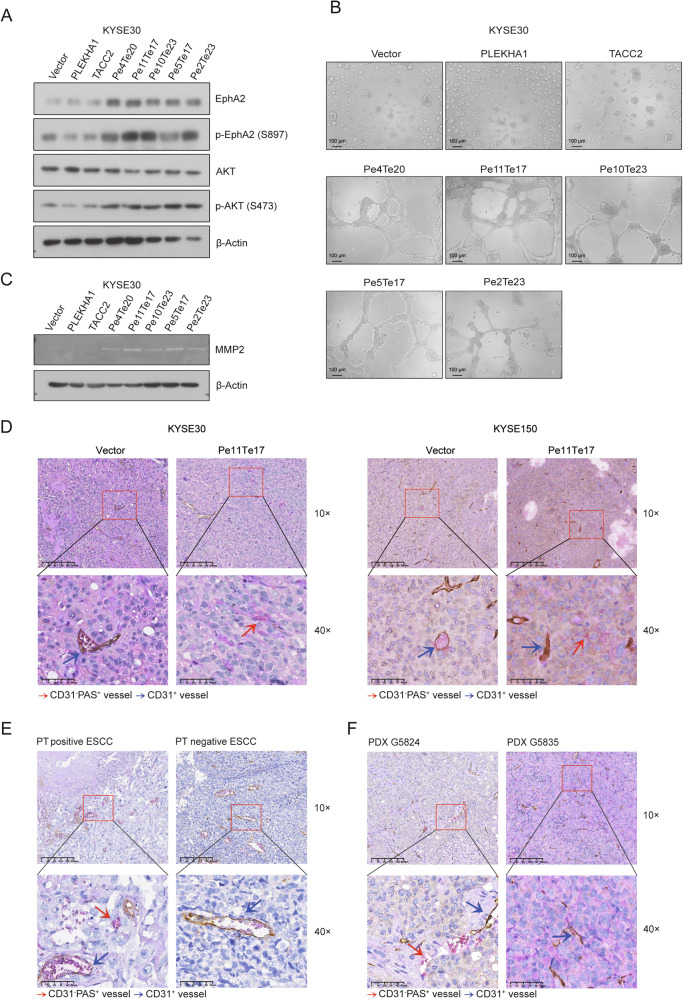
PLEKHA1-TACC2 promotes VM formation via activating the EphA2/AKT/MMP2 signalling pathway. **A** The lysates of KYSE30 cells stably expressing Flag-tagged PLEKHA1, TACC2, Pe4Te20, Pe11Te17, Pe10Te23, Pe5Te17, Pe2Te23, or an empty vector were blotted for total EphA2, p-EphA2 (S897), total AKT, and p-AKT (S473). **B** The VM ability of KYSE30 cells stably expressing Flag-tagged PLEKHA1, TACC2, Pe4Te20, Pe11Te17, Pe10Te23, Pe5Te17, Pe2Te23, or an empty vector was measured by an in vitro tube formation assay. **C** The conditioned medium of KYSE30 cells stably expressing Flag-tagged PLEKHA1, TACC2, Pe4Te20, Pe11Te17, Pe10Te23, Pe5Te17, Pe2Te23, or an empty vector was collected for gelatin zymography (top), and the cell lysates were blotted for β-Actin (bottom). **D**–**F** Representative images of VM in tumor tissues obtained from athymic nude mice bearing KYSE30 (left) or KYSE150 (right) tumors (**D**), ESCC patients (**E**), and PDX grown in mice (**F**). Red arrows: the tubule-like VM channels. Blue arrows: the blood vessels lined by CD31-positive endothelial cells. Scale bars were indicated in the images.

Phosphorylation of the intracellular domain of the EphA2 on serine 897 (S897) has been demonstrated to mediate EphA2 oncogenic activity and activating its downstream pathway, such as PI3K/AKT and MEK/ERK pathways [30]. Indeed, overexpression of PLEKHA1-TACC2 significantly increased the level of both total EphA2 and phospho-EphA2 (S897) (Figs.3A, S5A). Moreover, the phosphorylated AKT (S473) was induced in KYSE30 and KYSE150 cells expressing PLEKHA1-TACC2. These results demonstrated that PLEKHA1-TACC2 increased the level of phosphorylated EphA2 and activated p-AKT, as an EphA2 downstream target.

Previous studies have revealed that high EphA2 expression and signaling are frequently detected in many cancers and are associated with poor patient outcomes [31]. However, CCK8 assay showed that overexpression of PLEKHA1-TACC2 did not markedly promote cell proliferation in KYSE30 and KYSE150 cells (Fig. S5B). Additionally, immunoblotting analysis of the expression of stemness markers [32–36], including Nanog, KLF4, and SOX2, and sphere formation assay were performed in KYSE30 and KYSE150 cells. The results showed that PT expression did not significantly affect the expression of Nanog, KLF4, and SOX2 and the sphere-forming ability (Fig. S5C-D). The data suggested that PT might induce tumorigenesis through oncogenic functions other than cellular proliferation. A series of studies show that EphA2 is associated with VM in ESCC [37], gastric cancer [38], and prostate cancer [39]. To investigate the effect of PLEKHA1-TACC2 on VM formation, we performed 3D culture VM formation assay. After being seeded on a matrigel-coated plate for 16 h, KYSE30 and KYSE150 cells stably expressing PLEKHA1-TACC2 presented typical tubular shapes (Figs. 3B, S6A). Matrix metalloproteinase 2 (MMP2) is required for VM of tumor cells. The cleavage of the laminin 5 g2-chain by MMP2 into laminin 5 g2’ and g2x pro-migratory fragments could induce a vasculogenic phenotype in poorly aggressive tumor cells, resulting in the formation of VM. To assess whether PT affects the activity of MMP2, we performed gelatin zymography using the conditioned medium collected from KYSE30 and KYSE150 cells and found that overexpression of PLEKHA1-TACC2 upregulated the activity of MMP2 (Figs. 3C, S6B). To further demonstrate the formation of VM in vivo, we created a xenograft nude mice model by subcutaneously injecting KYSE30 and KYSE150 cells with Pe11Te17 overexpression alongside respective control cells. The results demonstrated that the volume and weight of xenograft tumors from Pe11Te17 overexpressed groups were significantly greater than those from the control group (Fig. S6C-H). CD31/Periodic Acid-Schiff (PAS) staining combined with Ter119 staining was utilized to analyse VM structure in tumor tissues, and the data showed that Pe11Te17 overexpression significantly increased the ability of VM formation in xenograft tumors from KYSE30 and KYSE150 cells (Fig. 3D).

Furthermore, CD31/PAS staining combined with CD235a or Ter119 staining was performed in the tumor tissues derived from the ESCC patients and PDX. PLEKHA1-TACC2 positive clinical ESCC tissues displayed increased VM channel formation compared to PLEKHA1-TACC2 negative ESCC tissues (Fig. 3E). Consistently, the VM channel formation in PDX G5824 tumor tissues was enhanced compared to PDX G5835 tissues (Fig. 3F). MK2206, a pan-AKT inhibitor, and doxycycline, an MMP2 inhibitor, significantly suppressed the vascular mimicry formation induced by PLEKHA1-TACC2 in KYSE30 and KYSE150 (Fig. S7). Together, these results confirm that the oncogenic PLEKHA1-TACC2 fusion protein activates the EphA2/AKT/MMP2 signaling pathway through stabilizing EphA2 expression and plays a vital role in tumor VM formation.

### Implication of PLEKHA1-TACC2 in targeted therapy

**Fig. 4:**
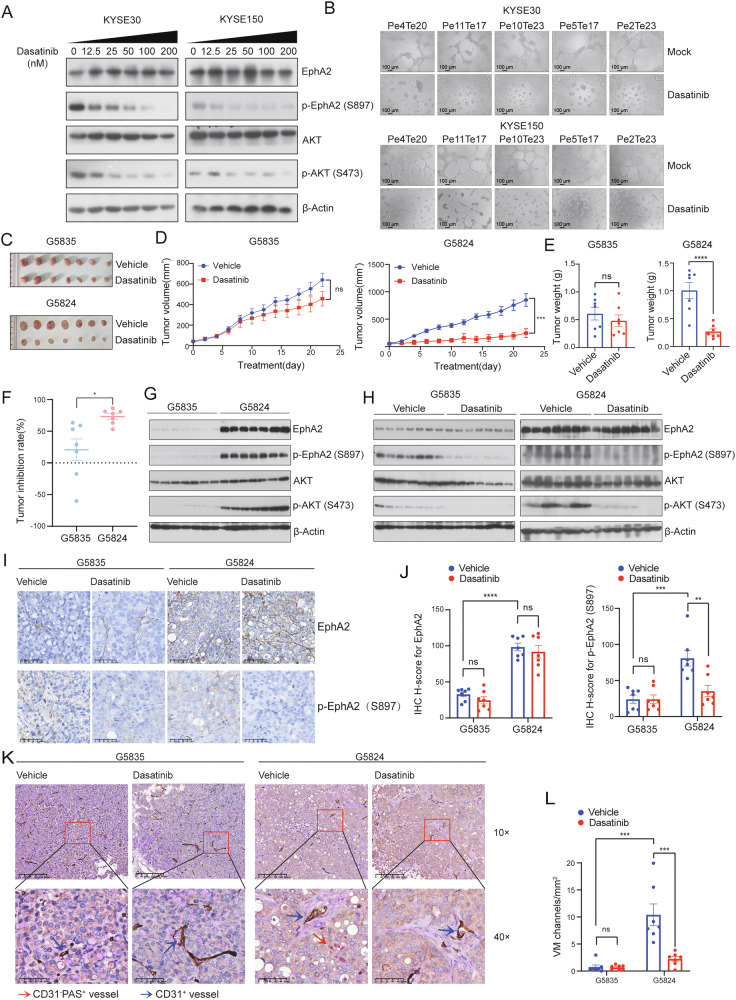
Dasatinib inhibits PLEKHA1-TACC2 activity and suppresses tumor growth. **A** The expression of total EphA2, p-EphA2 (S897), total AKT, and p-AKT (473) in KYSE30 and KYSE150 cells stably expressing Flag-tagged Pe4Te20 treated with indicated concentrations dasatinib for 48 h was determined by immunoblotting analyses. **B** The VM ability of KYSE30 and KYSE150 cells stably expressing Flag-tagged Pe4Te20, Pe11Te17, Pe10Te23, Pe5Te17, Pe2Te23, without or with dasatinib (50 nM) treatment was performed using an in vitro tube formation assay. **C** The images of PDX G5824 and G5835 tumors from mice treated with dasatinib or vehicle for 22 days (*n* = 7, each subgroup). **D** Growth curve of PDX G5835 (left) and G5824 (right) in mice treated with dasatinib or vehicle was plotted by measuring the relative tumor volume at indicated day. Data are presented as mean±s.e.m. The *P* values were calculated using the unpaired t-test. **E** Tumor weight of PDX G5835 (left) and G5824 (right) in mice treated with dasatinib or vehicle were measured at the endpoint of the experiment. Data are presented as mean±s.e.m. The *P* values were calculated using the unpaired t-test. **F** Tumor growth inhibition rates of dasatinib treatment on PDX G5835 and G5824. **G** The expression of total EphA2, p-EphA2 (S897), total AKT, and p-AKT (S473) in PDX G5835 and G5824 from mice with vehicle treatment was determined by immunoblotting analyses. **H** The expression of total EphA2 and p-EphA2 (S897), total AKT and p-AKT (S473) in PDX G5835 and G5824 from mice with dasatinib or vehicle treatment was determined by immunoblotting analyses. **I** The expression of total EphA2 and p-EphA2 (S897) in PDX G5835 and G5824 from mice with dasatinib or vehicle treatment was determined by IHC. Scale bars were indicated in the images. **J** H-score of EphA2 and p-EphA2 (S897) signal intensity in PDX G5835 and G5824 from mice with dasatinib or vehicle treatment as calculated by HALO analysis. The *P* values were calculated using one-way analysis of variance (ANOVA). **K** Representative images of CD31/PAS/Ter119 staining in tumor tissues of PDX G5835 and G5824 from mice with dasatinib or vehicle treatment. Red arrows: the tubule-like VM channels. Blue arrows: the blood vessels lined by CD31-positive endothelial cells. Scale bars were indicated in the images. **L** The number of VM channels in PDX G5835 and G5824 from mice with dasatinib or vehicle treatment as calculated by HALO analysis. The *P* values were calculated using one-way ANOVA. **P < 0.01, ***P < 0.001, ****P < 0.0001, ns indicates no significance.

Based on the above results, we investigated whether inhibiting the EphA2 kinase activity would impact downstream signaling responsible for tumor progression. Dasatinib is an oral dual Bcr/Abl and Src family kinases inhibitor approved for treating patients with chronic myelogenous leukaemia and has been shown to have significant activity against EphA2 [40, 41]. Indeed, dasatinib inhibited EphA2/AKT signaling pathway in a concentration dependent manner and remarkably restrained VM formation (Fig. 4A, B). These results indicated that dasatinib reversed the activated effects of PLEKHA1-TACC2 on the EphA2/AKT/MMP2 signaling pathway by affecting the phosphorylation of EphA2.

We then examined the in vivo effect of dasatinib. PDX G5835 and G5824 were separately transplanted into 14 nude mice each. When the tumors grew to about 5 mm in diameter, 7 PDX-bearing mice from each group were randomized to receive either dasatinib or the vehicle via intragastrical administration. Dasatinib administration effectively inhibited the progression of tumors derived from PDX G5824 but only slightly inhibited the progression of tumors derived from PDX G5835 (Fig. 4C-F), indicating that overexpression of PLEKHA1-TACC2 sensitizes tumor cells to dasatinib. We then examined levels of EphA2 and phospho-EphA2 in these tumors. IHC staining and immunoblotting analysis showed that the tumors derived from PDX G5824 had higher levels of EphA2 and phospho-AKT than tumors derived from PDX G5835 and that the PDX G5824-derived tumors from mice treated with dasatinib had reduced levels of phospho-EphA2 and phospho-Akt (Fig. 4G-J). CD31/PAS/Ter119 staining was utilized to assess VM formation in the tumor tissues; dasatinib obviously diminished the formation of VM channels (Fig. 4K, L). These results suggested that dasatinib significantly suppressed VM formation in vitro and in vivo, resulting in reduced tumor growth.

Considering that dasatinib is a multi-targeted kinase inhibitor, we verified the sensitivity of PLEKHA1-TACC2 positive tumors to ALW II-41-27, a small molecular EphA2 specific inhibitor that has been shown to block the phosphorylation of EphA2 [42]. We found that ALW II-41-27 significantly inhibited EphA2/AKT signaling pathway and VM formation induced by PLEKHA1-TACC2 in KYSE30 and KYSE150 cells in vitro (Fig. S8A-B) and suppressed the progression of PLEKHA1-TACC2 expressing tumors in vivo (Fig. S8C-L). Together, these results may pave the way for the development of the targeted therapy of dasatinib or ALW II-41-27 in ESCC harboring *PLEKHA1-TACC2* fusion gene.

### Oncogenic potential of PLEKHA1-TACC2 in ESCC

**Fig. 5:**
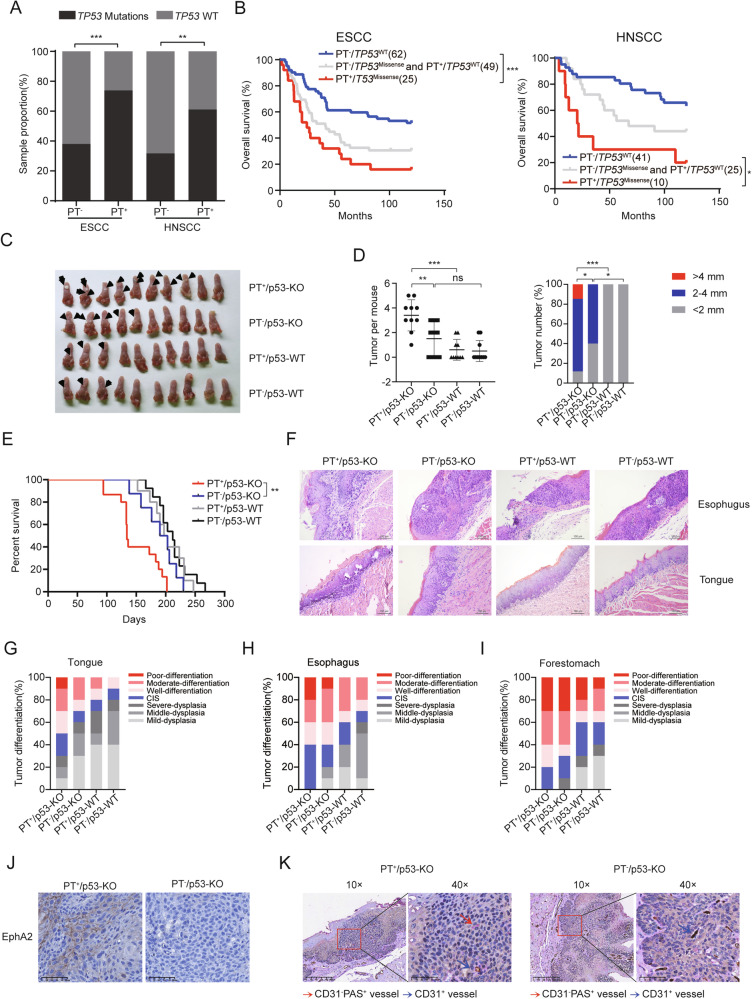
Oncogenic potential of the PLEKHA1-TACC2 fusion gene in vivo. **A** The prevalence of *TP53* mutations in human ESCC and HNSCC tissues harboring *PLEKHA1-TACC2* fusion. χ2 test was used for analysis. **B** Kaplan-Meier analysis of the OS in patients with ESCC or HNSCC grouped according to the expression of *PLEKHA1-TACC2* fusion and *TP53* missense mutations. The *P* values were calculated using the log-rank test. **C** The image of the tongue obtained from the indicated transgenic mice after 4-NQO treatment. Black arrow: the resulting tumors. **D** The number (left) and the size (right) of tumors from the indicated transgenic mice after 4-NQO treatment. The *P* values were calculated using one-way ANOVA (left) or contingency table analysis (right). **E** Kaplan-Meier analysis of the OS in the indicated transgenic mice after 4-NQO induction. The *P* values were calculated using the log-rank test. **F** Representative images of hematoxylin-eosin staining of esophagus and tongue obtained from the indicated transgenic mice after 4-NQO treatment. Scale bars were indicated in the images. **G**–**I** Tumor differentiation stages by histopathological analysis of the tongue (**G**), esophagus (**H**), and forestomach (**I**) from the indicated transgenic mice after 4-NQO treatment. **J** The expression of EphA2 in tumor tissues obtained from the indicated transgenic mice. Scale bars were indicated in the images. **K** Representative images of VM in tumor tissues obtained from the indicated transgenic mice. Red arrows: the tubule-like VM channels. Blue arrows: the blood vessels lined by CD31-positive endothelial cells. Scale bars were indicated in the images. *P < 0.05, **P < 0.01, ***P < 0.001, ns indicates no significance.

Previous studies indicated that mutations in the tumor suppressor *TP53* are frequently detected in some of the ESCC and HNSCC patients at the initial stage [43, 44] and are correlated with poor survival and high metastatic rates [45, 46]. Thus, we assessed the correlation of the presence of *PLEKHA1-TACC2* transcripts and the presence of *TP53* mutations in ESCC and HNSCC. We found that higher proportions of ESCC and HNSCC samples containing *PLEKHA1-TACC2* transcripts had *TP53* mutations (Fig. 5A). Positive PLA signals and TP53 expression were observed in ESCC tissues performed by Duolink PLA and IHC staining of TP53 (Fig. S9). Subsequently, we observed the types of *TP53* mutations in ESCC and HNSCC patients with *TP53* mutations, including missense, nonsense, and frame shift, among which the most patients harbored *TP53* missense mutation. Baseline characteristics of these patients are shown in Tables S3–4. Importantly, Kaplan–Meier analysis showed that *PLEKHA1-TACC2* expression combined with *TP53* missense mutation in ESCC and HNSCC was remarkably associated with poorer OS (Fig. 5B).

To better determine the oncogenic potential of *PLEKHA1-TACC2* in vivo, we crossed the transgenic mice overexpressing Pe4Te20 transcript that was one of recurrent transcripts and *Trp53* deletion mice to generate mice harboring both PT and *Trp53* deletion (PT^+^/p53-KO), mice with PT fusion only (PT^+^/p53-WT), mice with *Trp53 deletion* only (PT^-^/p53-KO) and the wild-type mice (PT^-^/p53-WT) (Fig. S10). After being treated with 4-nitroquilonine N-oxide (4-NQO) for 16 weeks followed by normal drinking water for another 12 weeks, all animals were sacrificed. We observed atypical hyperplasia lesions or squamous cell carcinoma of the tongue, esophagus, and stomach, 8/10 PT^+^/p53-KO mice, 5/10 PT^-^/p53-KO mice, 3/10 PT^+^/p53-WT mice and 3/10 PT^-^/p53-WT mice developed distinct squamous cell carcinoma of the tongue (Fig. 5C). Interestingly both tumor number and tumor size of PT^+^/p53-KO mice were larger than those of all other, including PT^-^/p53-KO mice (Fig. 5D), and PT^+^/p53-KO mice had worse prognosis (Fig. 5E). Consistently, histopathological analysis showed that PT^+^/p53-KO mice had the poorer differentiated tumor of the tongue, esophagus, and stomach (Fig. 5F-I). Furthermore, IHC staining of EphA2 and CD31/PAS/Ter119 staining showed that the EphA2 expression and the ability of VM formation were significantly upregulated in tumors derived from PT^+^/p53-KO mice compared to that derived from PT^-^/p53-KO mice (Fig. 5J, K). Our data indicated a strong oncogenic potential of PLEKHA1-TACC2 via promoting VM formation in ESCC.

**Fig. 6:**
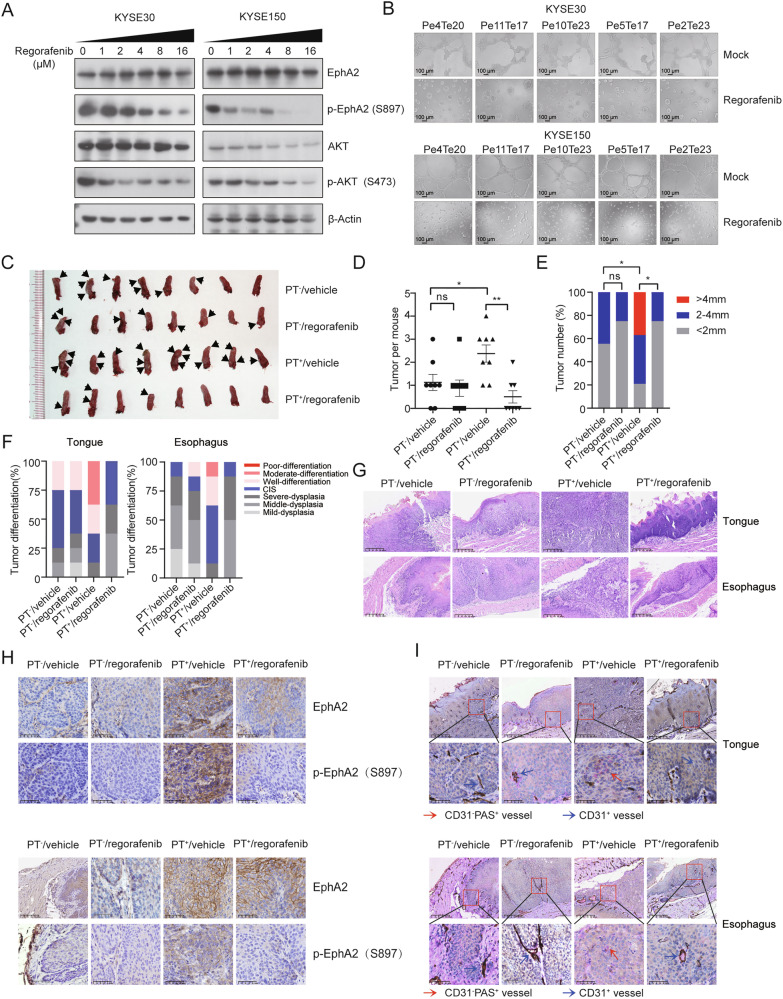
Regorafenib inhibits PLEKHA1-TACC2 activity and tumor progression. **A** The expression of total EphA2, p-EphA2 (S897), total AKT, and p-AKT (S473) in KYSE30 and KYSE150 cells stably expressing Flag-tagged Pe4Te20 treated with indicated concentration of regorafenib for 48 h was determined by immunoblotting analyses. **B** The VM ability of KYSE30 and KYSE150 cells stably expressing Flag-tagged Pe4Te20, Pe11Te17, Pe10Te23, Pe5Te17, Pe2Te23, without or with regorafenib (4 μM) treatment was performed using an in vitro tube formation assay. **C** The images of the tumors from the indicated transgenic mice following 4-NQO treatment first then 28 days of regorafenib or vehicle treatment (*n* = 8, each subgroup). **D** The number of tumors from the indicated transgenic mice following 4-NQO treatment first then 28 days of regorafenib or vehicle treatment. One-way ANOVA was used for analysis. **E** The size of tumors from the indicated transgenic mice following 4-NQO treatment first then 28 days of regorafenib or vehicle treatment. The *P* values were calculated using contingency table analysis. **F** Tumor differentiation stages by histopathological analysis of the tongue and esophagus from the indicated transgenic mice following 4-NQO treatment first then 28 days of regorafenib or vehicle treatment. **G** Representative images of hematoxylin-eosin staining of tongue and esophagus obtained from the indicated transgenic mice following 4-NQO treatment first then 28 days of regorafenib or vehicle treatment. Scale bars were indicated in the images. **H** The expression of total EphA2 and p-EphA2 (S897) in tongue (top) and esophagus (bottom) obtained from the indicated transgenic mice following 4-NQO treatment first then 28 days of regorafenib or vehicle treatment. Scale bars were indicated in the images. **I** Representative images of CD31/PAS/Ter119 staining of tongue and esophagus obtained from the indicated transgenic mice following 4-NQO treatment first then 28 days of regorafenib or vehicle treatment. Red arrows: the tubule-like VM channels. Blue arrows: the blood vessels lined by CD31-positive endothelial cells. Scale bars were indicated in the images. *P < 0.05, **P < 0.01, ns indicates no signifance.

In addition to dasatinib, regorafenib, a small molecular kinase inhibitor approved by the FDA to treat advanced solid tumors [47], has recently been reported to inhibit EphA2 Ser897 phosphorylation [48]. Our results showed that regorafenib strongly inhibited EphA2/AKT signaling and attenuated VM formation induced by PLEKHA1-TACC2 (Fig. 6A, B). We further investigated in vivo efficacy of regorafenib using transgenic mice and 4-NQO treatment as described earlier. PT^+^/p53-KO and PT^-^/p53-KO mice after 4-NQO treatment were randomized to receive either regorafenib or the vehicle via intragastrical administration for 28 days, all animals were then sacrificed, and their tongue and esophagus were examined for atypical hyperplasia lesions or squamous cell carcinoma. The tumor number and size of the tongue of PT^+^/p53-KO mice were larger than those of PT^-^/p53-KO mice and were significantly suppressed by regorafenib (Fig. 6C-E). Histopathological analysis showed that regorafenib treatment significantly improved the tumor differentiation grades of the tongue and esophagus in PT^+^/p53-KO mice (Fig. 6F, G). IHC staining showed that the tumors of tongue and esophagus of PT^+^/p53-KO mice showed higher expression of EphA2 compared with PT^-^/p53-KO mice and that regorafenib administration reduced the level of phospho-EphA2 in the tumors of tongue and esophagus of PT^+^/p53-KO mice (Fig. 6H). CD31/PAS/Ter119 staining was performed and showed obvious VM channels in the tumors of tongue and esophagus derived from PT^+^/p53-KO mice, and that regorafenib drastically inhibited VM formation (Fig. 6I). Our data indicated a promising therapeutic potential of regorafenib in PLEKHA1-TACC2 positive tumors.

To further evaluate the sensitivity of dasatinib, ALW-II-41-27 and regorafenib on ESCC cells by CCK8 assay, we found that the cell proliferate rates of KYSE30 and KYSE150 cells with PT expression upon the treatment of these inhibitors were similar to those of KYSE30 and KYSE150 cells without PT expression (Fig. S11A-B), suggesting that PLEKHA1-TACC2 didn’t affect the sensitivity on cell proliferation of these inhibitors. Growing evidence reported tumor-derived organoids have shown a great potential as a tool for precision medicine [49, 50]. To further explore the therapeutic potential of dasatinib, ALW II-41-27, and regorafenib, we established tumor organoids derived from PDX G5835 and G5824 models and were treated with these three inhibitors for 96 h, and the results indicated that the size and number of organoids derived from PDX G5824 models were significantly diminished after the treatment with dasatinib, ALW II-41-27, and regorafenib compared to those of organoids derived from PDX G5835 models (Fig. S11C), indicating organoids with PT expression are more sensitive to dasatinib, ALW II-41-27, and regorafenib treatment. The data suggest a promising therapeutic strategy of EphA2 inhibitors in ESCC patients with PLEKHA1-TACC2 expression.

## Discussion

In this study, we identified a fusion gene *PLEKHA1-TACC2* in a specimen of ESCC, which resulted from a genomic DNA rearrangement. Our results revealed several different isoforms of PT and PT was frequently detected in ESCC, HNSCC and various malignancy. Mass spectrometry analysis and Duolink PLA were performed to confirm that PT protein encoded by PT transcripts were present in tumors from patients. Kaplan–Meier analysis showed that the presence of PT transcripts in ESCC and HNSCC was significantly associated with poorer OS of patients. Mechanistically, we indicated PT overexpression promoted the formation of vascular mimicry by upregulating EphA2/AKT/MMP2 signalling pathway, and PT interfered with the interaction between c-cbl and EphA2, which inhibited the degradation of EphA2, by strongly interacting with EphA2. Additionally, we demonstrated that a strong oncogenic potential of PT in vivo, suggesting PT is a promising therapeutic marker in ESCC.

PLEKHA1 harbors two pleckstrin homology domains, including N-terminal and C-terminal PH domain. The PT transcripts encoding the fusion proteins were predicted to harbor the N-terminal PH domain found in PLEKHA1, locating upstream of the TACC domain [51]. Accumulating evidence suggests that the C-terminal PH domain of PLEKHA1 is highly selective for phosphatidylinositol 3,4-bisphosphate (PI(3,4)P2) and not phosphatidylinositol 3,4,5-trisphosphate (PI(3,4,5)P3), suggesting that PLEKHA1 participated in PI(3,4)P2-mediated signaling [52]. However, there have been few reports on the function of the N-terminal PH domain of PLEKHA1. Our study demonstrates that the fusion protein PLEKHA1-TACC2 interacts with EphA2, likely owing to the interaction between the N-terminal PH domain of PLEKHA1 and EphA2. While we have demonstrated that some of in-frame fusion transcripts of PLEKHA1-TACC2 could activate the EphA2/AKT/MMP2 signaling pathway, other transcripts were predictably able to encode PT fusion protein or truncated PLEKHA1 protein, the function of which might be in consistency with the transcripts that have been verified and requires additional investigation.

In addition to the important functions in the process of mitosis, TACC2, one of fusion partners, has been identified as a tumor suppressor gene in breast cancer [53] and hepatocellular carcinoma [54], the downregulation of which is an important step to drive tumorigenesis. Our previous study revealed inactivation of TACC2 significantly enhance tumor growth in ESCC [55]. Lack of TACC2 expression caused by PT fusion might have a potential role in PT positive tumor progression. In addition, TACC family genes have been demonstrated to fuse with a variety of genes to drive tumorigenesis. For example, FGFR-TACCs fusion proteins could result in the activation of the PI3K/AKT/mTOR signalling pathway, resulting leading in to abnormal expression of the cell cycle related genes CDK4, CDK2 and CCNE1 [56]. However, there was few studies to explore the role of 3’-UTR of TACC2 within fusion genes in tumor progression. All PT transcripts harbor 3’-UTR of TACC2, the role of the noncoding sequence needs to be further examined.

This study has demonstrated the role of the novel fusion gene *PLEKHA1-TACC2* in VM formation and its potential as a biomarker for effective ESCC treatment, but it nonetheless has certain limitations. Although this study has successfully identified the genomic breakpoint of *PLEKHA1-TACC2* in ESCC 1, genomic breakpoints in other tumors harboring *PLEKHA1-TACC2* transcripts could not be assessed owing to technical limitations of long-range PCR. Deep whole genome sequencing (WGS) needs to be conducted to survey entire DNA sequencing in tumors, which is cost-prohibitive in both research and clinical settings. Furthermore, we could not detect the endogenous fusion protein in ESCC clinical samples through mass spectrum owing to the limited protein content and the accessibility of patients’ specimens, which brings challenges in clinical investigation. We have demonstrated that DuoLink PLA could be a convenient and accurate technology to assess the endogenous protein in patients’ tissues. Further investigation of more patients’ specimens needs to be performed. Moreover, our data showed that PT promoted tumor progression, at least in part, via increased ability of vascular mimicry formation. However, the role of PT on other tumor intrinsic phenotype needs further investigation in future study.

A variety of fusion genes have been identified as biomarkers and therapeutic targets in multiple cancer types. The best examples of these are fusions involving kinases, which often result in enhanced or dysregulated kinase activity. Many kinase inhibitors successfully target these kinase fusions and provide better disease management options [14, 57]. For instance, FGFR-TACC fusions are frequently found in glioblastoma multiforme (GBM) and have been demonstrated to be transforming and oncogenic [58, 59]. Clinical data have shown promising therapeutic effects of FGFR inhibitors in GBM patients whose tumors harbor FGFR-TACC fusions [60]. ROS1 fusions have been examined in a variety of human malignancies, including NSCLC, cholangiocarcinoma and GBM. Crizotinib and entrectinib have shown marked activity against ROS1-positive advanced NSCLC [61, 62]. NTRK fusion oncoprotein has been shown to activate aberrant TRK signalling and acted as oncogenic drivers of various adult and paediatric tumor types [63]. Entrectinib and larotrectinib are effective in patients with tumors harboring NTRK fusions [64, 65]. Apart from kinases activated by gene fusions, MAML3 fusion gene could upregulate the Wnt signalling pathway and may be associated with an increased growth rate in pheochromocytomas and paragangliomas. WNT inhibitors may be clinically active in malignant tumors [66]. Our data show that PLEKHA1-TACC2 fusion protein could activate EphA2 signalling. In addition, various EphA2 inhibitors, including dasatinib, ALW II-41-27 and regorafenib could significantly inhibit vascular mimicry formation and the progression of PLEKHA1-TACC2 positive tumors in vitro and in vivo. Moreover, our data suggest PLEKHA1-TACC2 did not modulate the impact of dasatinib, ALW II-41-27, and regorafenib on cell proliferation.  This indicates that the primary role of these inhibitors on PLEKHA1-TACC2 positive tumor progression likely involves the inhibition of vascular mimicry, which might be independent of the inhibition of cell proliferation. Among these inhibitors, regorafenib has been approved by FDA for the treatment of multiple solid tumors with favorable safety and tolerability. Our results provide a strong rationale for clinical investigation of EphA2 inhibitor or its downstream inhibitor in patients having ESCC with PT fusions, which might also be useful in other types of cancers exhibiting PT fusions, besides ESCC.

## Materials and methods

### Cell lines, tumor xenografts, and patient samples

KYSE30 cell lines were gifted from Professor Yan Li. KYSE150 cell lines were gifted from Professor Dongxin Lin. HEK293T cells were purchased from ATCC (https://www.atcc.org/). All cells were grown in DMEM (Gibco-BRL Life Technologies, Carlsbad, USA) supplemented with 10% (vol/vol) fetal bovine serum (FBS; Gibco-BRL Life Technologies). All cell lines were maintained at 37 °C in a humidified incubator with 5% CO2. For experiments involving stable overexpression genes, cells were infected with the viral particles for 24 h and selected with 1 μg/mL puromycin for 72 h before being used for further experiments. All cell lines were authenticated with short tandem repeat DNA profiling and checked *Mycoplasma* free.

PDX models of human gastric cancer (G5820, G5824, G5826, and G5835) were gifted from Professor Yongzhan Nie [67]. The tumor xenografts were grafted in athymic nude mice.

Fresh frozen tissues for PCR analysis were surgically resected from histopathologically and clinically diagnosed patients with ESCC, HNSCC, colorectal cancer, hepatocellular carcinoma lung cancer, gastric cancer or breast cancer by Department of Pathology at Sun Yat-Sen University Cancer Center (SYSUCC). All formalin-fixed, paraffin-embedded (FFPE) ESCC samples were acquired from Department of Pathology of SYSUCC. 404 ESCC patients and 402 HNSCC patients with detailed long-term follow-up clinical data were used for analysis the association between *PLEKHA1-TACC2* expression and overall survival, among which 146 ESCC patients and 78 HNSCC patients were tested *TP53* status and used to survival analysis based on *PLEKHA1-TACC2* expression and *TP53* status.

### Library construction and sequencing

Poly(A) RNA was isolated from total RNA using oligo d(T) magnetic beads (New England Biolabs; S1419S). Purified poly(A) RNA was treated with DNase I (ThermoFisher; EN0521) and then randomly fragmented via fragmentation buffer (New England Biolabs; E6150S). The first and second cDNA strand were synthesized with random hexamer primers by using Superscript II Transcription Kit (ThermoFisher; 18064014) and RNase H (New England Biolabs; M0297S) plus DNA polymerase, respectively. The end of the cDNA fragments was repaired by Klenow polymerase (New England Biolabs; M0210), T4 DNA polymerase (New England Biolabs; M0203), and T4 polynucleotide kinase (New England Biolabs; M0201) to create blunt-ended DNA. A single 3’ adenosine moiety was added to the DNA, and Illumina PE adapters were ligated to the ‘A’ base on the repaired DNA ends. Size selection (200 base pair) of the DNA fragments on agarose gels was performed by cutting the target fragments to recover ligation products. The libraries were then amplified by PCR with Phusion DNA polymerase and primers containing barcode sequence to distinguish different libraries. Paired-end sequencing was performed using Illumina HiSeq^TM^ 2000 platform.

### Sequence data processing and alignment

The human reference genome assembly hg18 and annotated transcript set were obtained from UCSC and NCBI RefSeq database, respectively. The raw reads generated by the sequencer were firstly processed by removing reads containing sequencing adaptors and reads with low quality to get clean reads. The clean reads were then aligned to the human reference genome (hg18) and reference transcripts from NCBI Refseq database using SOAP2. A maximal of four mismatches were allowed for each hit.

### Detection of gene fusions

Gene fusions were identified using in-house software. Two types of sequencing reads were used to identify fusion genes: the fusion-spanning reads, which span the fusion junction, and the fusion-encompassing reads, which are mate pair reads aligned to two different genes. Firstly, all sequencing reads were aligned to the human reference genome and human reference transcripts from NCBI RefSeq database. Mate pairs aligning to multiple positions of the genome were discarded. Potential fusion gene partners were reported if mate pairs aligned to two different genes. We next enumerated all possible fusion junctions between the two fusion pairs and reads that cannot be mapped to the human genome, Refseq transcripts were aligned to fusion junctions to detect fusion-spanning reads. Fusion-spanning reads were required to span the fusion junction site no less than 8bp. At least one fusion-spanning read and one fusion-encompassing read are required to identify a fusion gene.

### Detection of *PLEKHA1-TACC2*

The fusion transcripts derived from *PLEKHA1* and *TACC2* were detected by RT-PCR combined with Sanger sequencing. Total RNA was isolated from the clinical biopsies by an E.Z.N.A. Total DNA/RNA Isolation Kit according to the manufacturer’s instructions (OMEGA Bio-Tek; R6731). The RNA was eluted with 50 μL nuclease-free water. The concentration and quantity of RNA were determined with a NanoDrop spectrophotometer (ThermoFisher; ND-1000). We performed reverse transcription (RT) with an oligo (dT) primer and the total RNA followed by PCR with the primers PT-RT-Sense (5′-TCGTCAGAATCGCATTTGTGG-3′) and PT-RT-Antisense (5′-GCTGCTCACCCTGGGAACTT-3′). The PLEKHA1-TACC2 fusion gene was detected by PCR, using genomic DNA of the clinical specimen from ESCC 1 extracted from the clinical biopsies by an E.Z.N.A. Total DNA/RNA Isolation Kit as the template. Amplification was performed with LA Taq Hot Start Version (Takara; RR042Q) and the primers PT-genome-Sense (5′-TCGTCAGAATCGCATTTGTGG-3′) and PT-genome-Antisense (5′-GCTGCTCACCCTGGGAACTT-3′). All the PCR products were subjected to sequencing.

### DNA constructs, virus production, and establishment of stable cell lines

PCR-amplified CDS region of human TACC2 and human PLEKHA1 was cloned into pHAGE vector (#118692; Addgene) (NotI/XhoI) with Flag-epitope-tag. The DNA fragments of fusion transcripts were obtained from the two plasmids DNA templates mentioned above. The expression plasmids for Flag-epitope tagged *PLEKHA1-TACC2* fusion transcripts were generated with pHAGE vector using multi-fragment homologous recombination. PCR-amplified CDS region of human EPHA2 and Ubiquitin were cloned into pHAGE with Myc-tag or HA-tag. All the primers sequences were provided in Table S5.

For generation of lentivirus particles, the lentiviral vectors along with psPAX2 (#12260; Addgene) and pMD2G (#12259; Addgene) (1:1:2 molar ratio) were transfected into HEK-293T cells using PEI. The supernatant containing virus particles was filtered through a 0.45-μm syringe filter after 48 h. KYSE30 and KYSE150 cell lines were infected by lentiviruses for 24 h, and cells were selected by 1 μg/mL puromycin for 3 days.

### In vitro vasculogenic mimicry tube formation assay

To assess the vascular mimicry ability of ESCC cells, a matrigel-based in vitro tube formation assay was applied. Briefly, 40 μL/well of matrigel (Corning; 354234) was plated in 96-well culture plate, and the culture plate was incubated at 37 °C for 30 min. Then 1 × 10^5^ cells/well were seeded on matrigel pre-coated wells. The cells were incubated in 5% CO2 incubator at 37 °C for 16 h and the VM structure could be seen. For drugs sensitivity test, ESCC cells were pre-treated separately with MK2206 (500 nM; Selleck; S1078), doxycycline (125 ng/mL; MCE; HY-N0565), dasatinib (50 nM; Selleck; S1021), ALW II-41-27 (125 nM; Selleck; S6515) or regorafenib (4 μM; Selleck; S11784) for 48 h and then the cells were seeded on matrigel pre-coated wells and incubated for 16 h, with each group comprising five independent biological replicates. VM structures were captured with a phase contrast microscope (Zeiss Axiovert 40c) at 10× field.

### IHC analysis and VM staining

The tumor tissues from clinical ESCC patients or mice were fixed in formalin and embedded in paraffin. Briefly, the tissue samples were cut into 4-μm sections and heated at 65 °C for 2 h. Next, the FFPE sections were deparaffinized in xylene and rehydrated in graded alcohol solutions ending with distilled water. The sections were treated with high temperature antigen retrieval in ethylenediaminetetraacetic acid (EDTA) solution (pH 8.0) for 2.5 min after blocking endogenous peroxidase activity using 3% hydrogen peroxide. After the retrieval solution had cooled to room temperature, sections were blocked with 5% BSA and incubated with diluted anti-EphA2 antibody (rabbit monoclonal, 1:200; Cell Signaling Technology; 6997S), anti-phospho EphA2 antibody (rabbit monoclonal, 1:100; Cell Signaling Technology; 6437S) or anti-TP53 antibody (rabbit monoclonal, 1:200; Cell Signaling Technology; 2527) overnight at 4 °C. The sections were washed and incubated with secondary antibody at 37 °C for 30 min. After washing, the sections were stained with 3,3’-diaminobenzidine (DAB) until the desired intensity was achieved. Sections were subsequently counterstained with haematoxylin to visualize nuclei and preserved in neutral balsam (ZSGB-BIO). For VM staining, the sections were incubated with CD235a (1:1000; ZSGB-BIO; ZA-0620) or Ter119 (1:500; Biolegend; 116201) antibody at 37 °C for 30 min followed CD31 (1:5000; Abcam; ab281583) IHC staining. After the sections were washed three times for 5 min each time, the sections were incubated with AP-labeled secondary antibodies at 37 °C for 30 min, signal detection was performed using fast red dye reagent. The PAS staining was performed followed CD235a or Ter119 staining. CD31-negative, PAS-positive vascular-like patterns containing red blood cells formed by tumor cells were identified as positive for VM.

The histopathological investigation was determined by two pathologists independently based on the World Health Organization criteria. The tumor differentiation status from mice tissues was graded as well-differentiation, moderate-differentiation, poor-differentiation, CIS, severe-dysplasia, moderate-dysplasia or mild-dysplasia.

### Polyclonal antibodies preparation

An anti-PLEKHA1 (N-terminal) mouse polyclonal antibody and an anti-TACC2 (C-terminal) rabbit polyclonal antibody are performed as previously described [68], with minor modifications. In brief, PLEKHA1 (N-terminal) peptide (specific targeting of exon1-2 of PLEKHA1) and TACC2 (C-terminal) peptide (specific targeting of exon23 of TACC2) as specific antigens were separately injected into mice or rabbits. After the priming immunization 4–8 weeks, booster immunizations are performed at 2-to-3-week intervals. The serum of the animals was collected and detected antibody titer. When the antibody titer reached an acceptable level, the specific polyclonal antibodies were isolated from serum. The purity of polyclonal antibody is checked using immunoblotting.

### Duolink in situ proximity ligation assay

Duolink PLA kit was used to detect endogenous protein PT expression (Sigma-Aldrich; DUO92008). PLA was conducted in accordance with the manufacturer’s instructions. In short, the FFPE sections were deparaffinized in xylene and rehydrated in graded alcohol solutions. Antigen retrieval was performed in a pressure-boiling container with EDTA buffer (pH 8.0) for 2.5 min. After incubation with a blocking solution for 60 minutes at 37 °C, the sections were then incubated overnight with an anti-PLEKHA1 (N-terminal) mouse polyclonal antibody (1:200 dilution) and an anti-TACC2 (C-ternimal) rabbit polyclonal antibody (1:200 dilution) at 4 °C. Anti-rabbit MINUS and anti-mouse PLUS PLA probes (1:5 dilution) were used to incubate the slides for 60 min at 37 °C. After washing twice for 5 min each time in wash buffer A, the slides were incubated with 5X Ligation Stock (1:5 dilution, Duolink, Sigma-Aldrich) and Ligase (1:40 dilution, Duolink, Sigma-Aldrich) for 30 min at 37 °C, washed again in wash buffer A, and incubated in Amplification Stock 5X (1:5 dilution, Duolink, Sigma-Aldrich) and Polymerase (1:80 dilution, Duolink, Sigma-Aldrich) for 100 min at 37 °C, and washed twice for 10 min each time in wash buffer B. Finally, mounting medium with 4,6-diamidino-2-phenyl-indole (DAPI) was used to detect tissue nuclei. The slides were imaged using a confocal laser scanning microscope (LSM880 with fast airyscan).

### Co-immunoprecipitation and mass spectrometry analysis

Protein lysates from cells or tissues extracted from IP lysis buffer (50 mM Tris-HCl (pH 7.4), 150 mM NaCl, 0.6% NP-40, 5 mM EDTA) supplemented with complete protease (MedChem Express; HY-K0011) and phosphatase inhibitor cocktail (MedChem Express; HY-K0021) were incubated with Flag Labelled-Beads (Sigma-Aldrich; M8823), Myc Labelled-Beads (Biolegend; 658502) or anti-TACC2 antibody-conjugated protein A/G agarose beads (Thermo Fisher) overnight at 4°C. After washing with IP wash buffer (50 mM Tris-HCl (pH 7.4), 150 mM NaCl, 0.6% NP40, 5 mM EDTA), the complexes were eluted to further western blotting or silver staining. The indicated bands were analysed by mass spectrometry.

### Immunoblotting, protein degradation, and ubiquitylation

Protein lysates from cells or tissues were extracted using RIPA buffer (50 mM Tris HCl pH 7.4, 150 mM NaCl, 1% NP-40, 0.5% sodium deoxycholate, 0.1% SDS, and 1 mM phenylmethyl sulfonyl fluoride) with complete protease (MedChem Express; HY-K0011) and phosphatase inhibitor cocktail (MedChem Express; HY-K0021) and quantified with a BCA kit (Thermo Fisher; 23225). The protein lysates were mixed with loading buffer and then separated by SDS-PAGE gel electrophoresis. Next, proteins were transferred onto PVDF membranes (Millipore). The membrane blocked with 5% BSA for 60 min at room temperature was incubated with primary antibody overnight at 4 °C and a HRP-conjugated secondary antibody for 45 min at RT. Membranes were detected using an ECL detection kit (Advansta, 141104-03). To examine protein degradation, cells were treated with CHX (100 μg/mL, MedChemExpress; hy-12320) for several time points, followed by western blotting. To examine the ubiquitylation level of EphA2, cells were transfected with Myc-tagged EphA2 and HA-tagged Ubiquitin before harvested. Protein lysates extracted by IP lysis buffer were incubated with Myc-beads (Biolegend; 658502) overnight at 4 °C. After washing, proteins were eluted and followed by western blotting detection using an anti-HA antibody.

The antibodies utilized include the following: anti-EphA2 (1:1000; Cell Signaling Technology; 6997S); anti-β-Actin (1:4000; Cell Signaling Technology; 4970S); anti-Flag (1:1000; Proteintech; 20543-1-AP-100μl); anti-Myc (1:1000; Cell Signaling Technology; 2278S); anti-HA (1:500; Santa Cruz; sc-7392); anti-p-EphA2 (S897) (1:1000; Cell Signaling Technology; 6437S); anti-AKT (1:1000; Cell Signaling Technology; 9272S), anti-p-AKT (S473) (1:1000; Cell Signaling Technology; 4060S), anti-Nanog (1:500; Cell Signaling Technology; 4903P), anti-KLF4 (1:500; Cell Signaling Technology; 4038T), anti-SOX2 (1:500; Proteintech; 11064-1-AP-50ul) and anti-α-Tubulin (1:4000; Cell Signaling Technology; 2125S). All the uncropped images of gels and blots were provided in the Supplementary Materials.

### Gelatin zymography assay

Gelatin zymography was used to measure MMP2 and MMP9 activity. Briefly, cells were incubated in DMEM without FBS for 24 h. The cell supernatants were collected and mixed with 5× zymography sample buffer and then loaded into each lane of a SDS-PAGE (10%) gel containing 1 mg/mL gelatin. After electrophoresis, the gel was washed for 30 min in zymogram renaturing buffer (2.5% Triton X-100) with gentle agitation at room temperature to remove SDS and incubated in reaction buffer (50 mM Tris–HCl pH 7.4, 200 mM NaCl, and 5 mM CaCl2) at 37 °C overnight. After staining with Coomassie brilliant blue, MMP activity was visualized as clear zones against blue background.

### PDX-derived organoids culture and EphA2 inhibitors tests

PDX-G5835 and G5824 tissues were separated and rinsed three times with cold PBS. Following washing, the tissues were minced into small cubes, approximately 1 mm³ in size, and transferred into a fresh 50 mL centrifuge tube. 15 mL of 0.25% trypsin-EDTA (Gibco, Cat# 25200072) was added and used to digest the tissues at 37 °C for 1 h. The digestion process was halted using 15 mL of DMEM supplemented with 10% FBS. Single-cell suspension was obtained by filtering the digested mixture through a 70 µm cell strainer and then centrifuging at 400 × *g* for 10 min. After centrifuging, the cell pellet was resuspended with Matrigel, and 30 µL of this mixture were plated at the bottom of 48-well plates to form a hemispherical shape upon incubation at 37 °C for 15 min. Once solidified, the organoids were cultured in 500 µL of the Gastric Cancer Organoid Kit (bioGenous, K2179-GC). After 3–4 days, PDX-derived organoids would be seen. For the sensitivity detection of EphA2 inhibitors, PDX-derived organoids were digested with 0.25% trypsin-EDTA at 37 °C for 15 min, and DMEM with 10% FBS was used to stop the digestion reaction. Single-cell suspension was obtained as above mentioned, and 4000 single cells were cultured in a 48-well plate embedded in 30 µL Matrigel. After 1 day, the organoids were treated with dasatinib (50 nM), ALW II-41-27 (125 nM), or regorafenib (4 µM) separately for 96 h, with each group comprising five independent biological replicates. The organoids structures were captured with a phase contrast microscope (Zeiss Axiovert 40c) at 10× field.

### Animal experiments

Female nude mice (4–5 weeks old) were purchased from Beijing Vital River Laboratory Animal Technology Co., Ltd. and housed in microisolator cages.

To generate subcutaneous ESCC tumors in nude mice, 1 × 10^6^ KYSE30 or KYSE150 cells stably expressing Flag-tagged Pe11Te17 or an empty vector were inoculated subcutaneously into 4 to 5-week-old nude mice. For in vivo studies involving dasatinib, PDX G5835 and G5824 were separately transplanted into nude mice. When the tumors had grown to 50–100 mm^3^, dasatinib (30 mg/kg/i.g.) or vehicle (0.5% Carboxymethylcellulose sodium in sterile water) was injected once daily for 22 days. For in vivo studies involving ALW II-41-27, treatment (10 mg/kg/i.p., twice daily for 14 days) started when the tumors had grown to 50–100 mm^3^. The vehicle for ALW II-41-27 was 5% DMSO, 40% PEG300, 5%Tween 80 in sterile water. Tumor dimensions were measured using a Vernier calliper, and tumor volume was calculated according to the formula V = 0.52 × (a^2 ^× b), where a and b are the minimal and maximal diameters, respectively, in millimetres. The tumor growth inhibition rate (%) was calculated using the following formula: [1 − (Ti − T0)/(Vi − V0)] × 100%. (Ti: Mean tumor volume of the treatment group at time of tumor extraction, T0: Mean tumor volume of the treatment group at the start of administration; Vi: Mean tumor volume of the vehicle group at time of tumor extraction, V0: Mean tumor volume of the vehicle group at the start of administration). In accordance with institutional guidelines, mice bearing subcutaneous xenografts smaller than 2000 mm^3^ were sacrificed. Explanted tumors were weighed and photographed.

We used the ED-L2 promoter of Epstein-Barr virus, which drives epithelial-specific expression in the tongue, esophagus, and squamous forestomach [69], to overexpress the Pe4Te20 transcript in oral-esophageal epithelia of C57BL/6 J mice (PT^+^ mice). The p53 deletion mice (B6.129S2-Trp53^tm1Tyj^/J) were developed in the laboratory of Dr. Tyler Jacks at the Center for Cancer Research, Massachusetts Institute of Technology [70]. PT^+^ mice were crossed to p53 deletion mice to obtain PT^+^/p53-KO, PT^+^/p53-WT, PT^-^/p53-KO, and PT^-^/p53-WT mice. For genotyping, genomic DNA was extracted and purified from mouse tails as templates. Amplification was performed using 2 × SuperNova PCR Mix (GenStar; A065). Cycling conditions were as follows: initial denaturation at 94 °C for 5 min followed by 35 cycles of 94 °C for 1 min, 58 °C for 30 s, and 72 °C for 40 s, and finally 1 cycle at 72 °C for 5 min. All the primer sequences were provided in Table S6. The PCR product size of the wild‑type p53 is 288 bp, whereas that of the p53-KO is 319 bp. The PCR product size of PT fusion is 1032 bp. For 4-NQO-induced ESCC animal model, 6-week-old PT^+^/p53-KO, PT^+^/p53-WT, PT^-^/p53-KO, and PT^-^/p53-WT BALB/c nude mice were fed with water containing 4-NQO (100 μg/mL; Meilunbio; MB6089) for 16 weeks then with normal drinking water for another 12 weeks. Mice were then sacrificed, and their tongue, esophagus, and stomach were obtained for further histopathological analysis. For 4-NQO-induced ESCC animal model involving regorafenib, 6-week-old PT^+^/p53-KO and PT^-^/p53-KO C57BL/6 J mice were exposed to water containing 4-NQO (100 μg/mL) for 16 weeks followed by normal drinking water for another 4 weeks. Then the mice were divided into two groups randomly and treated with regorafenib (80 mg/kg/i.g. once daily) or vehicle for 28 days separately. The vehicle for regorafenib was 0.5% CMC-Na in sterile water. The tongue and esophagus were collected for further histopathological analysis.

### Statistical analysis

All experiments for quantitative analysis and representative images were repeated at least three times. Data sets with normal distribution were analyzed with unpaired Student’s two-sided t-tests to compare two groups. Two-sided log-rank (Mentel–Cox) test was used to evaluate the Kaplan–Meier survival analyses. Multivariate Cox regression model was performed to analyse the baseline variables for prognostic significance. Associations between the prevalence of TP53 mutations and PLEKHA1-TACC2 expression in ESCC and HNSCC patients were analysed using the χ2 test. Contingency table analysis was performed to evaluate the significance of tumor size in different groups in transgenic mice model. One-way ANOVA was performed to evaluate the difference among three or more groups. *P* < 0.05 was considered statistically significant (*P < 0.05, **P < 0.01, ***P < 0.001, ****P < 0.0001, ns indicates no significance). All statistical analyses were performed by GraphPad Prism 9.3.1 software.

The sample size was not predetermined through statistical tests but was based on previously published findings that identified significant differences. All collected samples were included in the analyses without exclusion. And the investigators were not blinded during the experimental procedures or outcome assessments.

## Supplementary information

Supplementary material

aj-checklist

Westwen blotting data

## Data Availability

The raw sequence data for the study have been deposited in the Genome Sequence Archive of the BIG Data Center at the Beijing Institute of Genomics, Chinese Academy of Science, under accession number HRA010205 (http://bigd.big.ac.cn/gsa-human). The raw data of all the functional experiments of the main figures in this work have been deposited onto the Research Data Deposit public platform (www.researchdata.org.cn) with the approval number RDDB2025850433.

## References

[1] Jemal A, Bray F, Center MM, Ferlay J, Ward E, Forman D. Global cancer statistics. CA Cancer J Clin. 2011;61:69–90. 21296855 10.3322/caac.20107

[2] Wouters MWJM, Gooiker GA, van Sandick JW, Tollenaar RAEM. The volume-outcome relation in the surgical treatment of esophageal cancer: a systematic review and meta-analysis. Cancer. 2012;118:1754–63. 22009562 10.1002/cncr.26383

[3] Ohashi S, Miyamoto S, Kikuchi O, Goto T, Amanuma Y, Muto M. Recent advances from basic and clinical studies of esophageal squamous cell carcinoma. Gastroenterology. 2015;149:1700–15. 26376349 10.1053/j.gastro.2015.08.054

[4] Gniadecki R, Iyer A, Hennessey D, Khan L, O’Keefe S, Redmond D, et al. Genomic instability in early systemic sclerosis. J Autoimmun. 2022;131:102847. 35803104 10.1016/j.jaut.2022.102847

[5] Brawand D, Soumillon M, Necsulea A, Julien P, Csárdi G, Harrigan P, et al. The evolution of gene expression levels in mammalian organs. Nature. 2011;478:343–8. 22012392 10.1038/nature10532

[6] King MC, Wilson AC. Evolution at two levels in humans and chimpanzees. Science. 1975;188:107–16. 1090005 10.1126/science.1090005

[7] Branford S, Yeung DT, Ross DM, Prime JA, Field CR, Altamura HK, et al. Early molecular response and female sex strongly predict stable undetectable BCR-ABL1, the criteria for imatinib discontinuation in patients with CML. Blood. 2013;121:3818–24. 23515925 10.1182/blood-2012-10-462291

[8] Clarke M, Mackay A, Ismer B, Pickles JC, Tatevossian RG, Newman S, et al. Infant high-grade gliomas comprise multiple subgroups characterized by novel targetable gene fusions and favorable outcomes. Cancer Discov. 2020;10:942–63. 32238360 10.1158/2159-8290.CD-19-1030 PMC8313225

[9] Li W, Liu Y, Li W, Chen L, Ying J. Intergenic breakpoints identified by DNA sequencing confound targetable kinase fusion detection in NSCLC. J Thorac Oncol Publ Int Assoc Study Lung Cancer. 2020;15:1223–31. 10.1016/j.jtho.2020.02.023 32151779

[10] Neumann O, Burn TC, Allgäuer M, Ball M, Kirchner M, Albrecht T, et al. Genomic architecture of FGFR2 fusions in cholangiocarcinoma and its implication for molecular testing. Br J Cancer. 2022;127:1540–9. 35871236 10.1038/s41416-022-01908-1 PMC9553883

[11] Terlecki-Zaniewicz S, Humer T, Eder T, Schmoellerl J, Heyes E, Manhart G, et al. Biomolecular condensation of NUP98 fusion proteins drives leukemogenic gene expression. Nat Struct Mol Biol. 2021;28:190–201. 33479542 10.1038/s41594-020-00550-w PMC7116736

[12] Singh VK, Coumar MS. Chronic myeloid leukemia: existing therapeutic options and strategies to overcome drug resistance. Mini Rev Med Chem. 2019;19:333–45. 30332954 10.2174/1389557518666181017124854

[13] Soverini S, Mancini M, Bavaro L, Cavo M, Martinelli G. Chronic myeloid leukemia: the paradigm of targeting oncogenic tyrosine kinase signaling and counteracting resistance for successful cancer therapy. Mol Cancer. 2018;17:49. 29455643 10.1186/s12943-018-0780-6 PMC5817796

[14] Schram AM, Chang MT, Jonsson P, Drilon A. Fusions in solid tumours: diagnostic strategies, targeted therapy, and acquired resistance. Nat Rev Clin Oncol. 2017;14:735–48. 28857077 10.1038/nrclinonc.2017.127 PMC10452928

[15] Solomon BJ, Kim D-W, Wu Y-L, Nakagawa K, Mekhail T, Felip E, et al. Final overall survival analysis from a study comparing first-line crizotinib versus chemotherapy in ALK-mutation-positive non-small-cell lung cancer. J Clin Oncol J Am Soc Clin Oncol. 2018;36:2251–8. 10.1200/JCO.2017.77.4794 29768118

[16] Drilon A, Subbiah V, Gautschi O, Tomasini P, de Braud F, Solomon BJ, et al. Selpercatinib in patients with RET fusion-positive non-small-cell lung cancer: updated safety and efficacy from the registrational LIBRETTO-001 phase I/II Trial. J Clin Oncol J Am Soc Clin Oncol. 2023;41:385–94. 10.1200/JCO.22.00393 PMC9839260 36122315

[17] Duke ES, Bradford D, Marcovitz M, Amatya AK, Mishra-Kalyani PS, Nguyen E, et al. FDA approval summary: selpercatinib for the treatment of advanced RET fusion-positive solid tumors. *Clin. Cancer Res.* **29**, 3573-8 (2023). 10.1158/1078-0432.CCR-23-0459 PMC10524590 37265412

[18] Hujanen R, Almahmoudi R, Karinen S, Nwaru BI, Salo T, Salem A. Vasculogenic mimicry: a promising prognosticator in head and neck squamous cell carcinoma and esophageal cancer? A systematic review and meta-analysis. Cells. 2020;9:507. 32102317 10.3390/cells9020507 PMC7072765

[19] Maniotis AJ, Folberg R, Hess A, Seftor EA, Gardner LM, Pe’er J, et al. Vascular channel formation by human melanoma cells in vivo and in vitro: vasculogenic mimicry. Am J Pathol. 1999;155:739–52. 10487832 10.1016/S0002-9440(10)65173-5 PMC1866899

[20] Liu T, Sun B, Zhao X, Li Y, Zhao X, Liu Y, et al. USP44+ cancer stem cell subclones contribute to breast cancer aggressiveness by promoting vasculogenic mimicry. Mol Cancer Ther. 2015;14:2121–31. 26232424 10.1158/1535-7163.MCT-15-0114-T

[21] Wang M, Zhao X, Zhu D, Liu T, Liang X, Liu F, et al. HIF-1α promoted vasculogenic mimicry formation in hepatocellular carcinoma through LOXL2 up-regulation in hypoxic tumor microenvironment. J Exp Clin Cancer Res CR. 2017;36:60. 28449718 10.1186/s13046-017-0533-1 PMC5408450

[22] Seftor RE, Seftor EA, Koshikawa N, Meltzer PS, Gardner LM, Bilban M, et al. Cooperative interactions of laminin 5 gamma2 chain, matrix metalloproteinase-2, and membrane type-1-matrix/metalloproteinase are required for mimicry of embryonic vasculogenesis by aggressive melanoma. Cancer Res. 2001;61:6322–7. 11522618

[23] Delgado-Bellido D, Fernández-Cortés M, Rodríguez MI, Serrano-Sáenz S, Carracedo A, Garcia-Diaz A, et al. VE-cadherin promotes vasculogenic mimicry by modulating kaiso-dependent gene expression. Cell Death Differ. 2019;26:348–61. 29786069 10.1038/s41418-018-0125-4 PMC6329820

[24] Tang N-N, Zhu H, Zhang H-J, Zhang W-F, Jin H-L, Wang L, et al. HIF-1α induces VE-cadherin expression and modulates vasculogenic mimicry in esophageal carcinoma cells. World J Gastroenterol WJG. 2014;20:17894–904. 25548487 10.3748/wjg.v20.i47.17894 PMC4273139

[25] Zhang C, Chen W, Zhang X, Huang B, Chen A, He Y, et al. Galunisertib inhibits glioma vasculogenic mimicry formation induced by astrocytes. Sci Rep. 2016;6:23056. 26976322 10.1038/srep23056 PMC4791658

[26] Lee MJ, Gergely F, Jeffers K, Peak-Chew SY, Raff JW. Msps/XMAP215 interacts with the centrosomal protein D-TACC to regulate microtubule behaviour. Nat Cell Biol. 2001;3:643–9. 11433296 10.1038/35083033

[27] Srayko M, Quintin S, Schwager A, Hyman AA. Caenorhabditis elegans TAC-1 and ZYG-9 form a complex that is essential for long astral and spindle microtubules. Curr Biol. 2003;13:1506–11. 12956952 10.1016/s0960-9822(03)00597-9

[28] Xiao T, Xiao Y, Wang W, Tang YY, Xiao Z, Su M. Targeting EphA2 in cancer. J Hematol Oncol. 2020;13:114. 32811512 10.1186/s13045-020-00944-9 PMC7433191

[29] Sabet O, Stockert R, Xouri G, Brüggemann Y, Stanoev A, Bastiaens PIH. Ubiquitination switches EphA2 vesicular traffic from a continuous safeguard to a finite signalling mode. Nat Commun. 2015;6:8047. 26292967 10.1038/ncomms9047 PMC4560775

[30] Miao B, Ji Z, Tan L, Taylor M, Zhang J, Choi HG, et al. EPHA2 is a mediator of vemurafenib resistance and a novel therapeutic target in melanoma. Cancer Discov. 2015;5:274–87. 25542448 10.1158/2159-8290.CD-14-0295 PMC4355085

[31] Zhou Y, Sakurai H. Emerging and diverse functions of the EphA2 noncanonical pathway in cancer progression. Biol Pharm Bull. 2017;40:1616–24. 28966234 10.1248/bpb.b17-00446

[32] MacDonagh L, Gray SG, Breen E, Cuffe S, Finn SP, O’Byrne KJ, et al. Lung cancer stem cells: the root of resistance. Cancer Lett. 2016;372:147–56. 26797015 10.1016/j.canlet.2016.01.012

[33] Silva J, Smith A. Capturing pluripotency. Cell. 2008;132:532–6. 18295569 10.1016/j.cell.2008.02.006 PMC2427053

[34] Young RA. Control of the embryonic stem cell state. Cell. 2011;144:940–54. 21414485 10.1016/j.cell.2011.01.032 PMC3099475

[35] Boyer LA, Lee TI, Cole MF, Johnstone SE, Levine SS, Zucker JP, et al. Core transcriptional regulatory circuitry in human embryonic stem cells. Cell. 2005;122:947. 16153702 10.1016/j.cell.2005.08.020 PMC3006442

[36] Bian X, Shi D, Xing K, Zhou H, Lu L, Yu D, et al. AMD1 upregulates hepatocellular carcinoma cells stemness by FTO mediated mRNA demethylation. Clin Transl Med. 2021;11:e352. 33783988 10.1002/ctm2.352 PMC7989706

[37] Li S, Meng W, Guan Z, Guo Y, Han X. The hypoxia-related signaling pathways of vasculogenic mimicry in tumor treatment. Biomed Pharmacother Biomed Pharmacother. 2016;80:127–35. 27133049 10.1016/j.biopha.2016.03.010

[38] Kim HS, Won YJ, Shim JH, Kim HJ, Kim J, Hong HN, et al. Morphological characteristics of vasculogenic mimicry and its correlation with EphA2 expression in gastric adenocarcinoma. Sci Rep. 2019;9:3414. 30833656 10.1038/s41598-019-40265-7 PMC6399224

[39] Yeo C, Lee H-J, Lee E-O. Serum promotes vasculogenic mimicry through the EphA2/VE-cadherin/AKT pathway in PC-3 human prostate cancer cells. Life Sci. 2019;221:267–73. 30797819 10.1016/j.lfs.2019.02.043

[40] Bantscheff M, Eberhard D, Abraham Y, Bastuck S, Boesche M, Hobson S, et al. Quantitative chemical proteomics reveals mechanisms of action of clinical ABL kinase inhibitors. Nat Biotechnol. 2007;25:1035–44. 17721511 10.1038/nbt1328

[41] Wang X-D, Reeves K, Luo FR, Xu L-A, Lee F, Clark E, et al. Identification of candidate predictive and surrogate molecular markers for dasatinib in prostate cancer: rationale for patient selection and efficacy monitoring. Genome Biol. 2007;8:R255. 18047674 10.1186/gb-2007-8-11-r255 PMC2258199

[42] Xiang Y-P, Xiao T, Li Q-G, Lu S-S, Zhu W, Liu Y-Y, et al. Y772 phosphorylation of EphA2 is responsible for EphA2-dependent NPC nasopharyngeal carcinoma growth by Shp2/Erk-1/2 signaling pathway. Cell Death Dis. 2020;11:1–14. 32848131 10.1038/s41419-020-02831-0 PMC7449971

[43] Chen X-X, Zhong Q, Liu Y, Yan S-M, Chen Z-H, Jin S-Z, et al. Genomic comparison of esophageal squamous cell carcinoma and its precursor lesions by multi-region whole-exome sequencing. Nat Commun 2017;8:524. 10.1038/s41467-017-00650-0 PMC5595870 28900112

[44] Kawakubo H, Ozawa S, Ando N, Kitagawa Y, Mukai M, Ueda M, et al. Alterations of p53, cyclin D1 and pRB expression in the carcinogenesis of esophageal squamous cell carcinoma. Oncol Rep. 2005;14:1453–9. 16273238 10.3892/or.14.6.1453

[45] Leemans CR, Braakhuis BJM, Brakenhoff RH. The molecular biology of head and neck cancer. Nat Rev Cancer. 2011;11:9–22. 21160525 10.1038/nrc2982

[46] Song Y, Li L, Ou Y, Gao Z, Li E, Li X, et al. Identification of genomic alterations in oesophageal squamous cell cancer. Nature. 2014;509:91–5. 24670651 10.1038/nature13176

[47] Kudo M. Regorafenib as second-line systemic therapy may change the treatment strategy and management paradigm for hepatocellular carcinoma. Liver Cancer. 2016;5:235–44. 27781196 10.1159/000449335 PMC5075814

[48] Yan H, Wu W, Hu Y, Li J, Xu J, Chen X, et al. Regorafenib inhibits EphA2 phosphorylation and leads to liver damage via the ERK/MDM2/p53 axis. Nat Commun. 2023;14:2756. 37179400 10.1038/s41467-023-38430-8 PMC10182995

[49] Veninga V, Voest EE. Tumor organoids: opportunities and challenges to guide precision medicine. Cancer Cell. 2021;39:1190–201. 34416168 10.1016/j.ccell.2021.07.020

[50] Tong L, Cui W, Zhang B, Fonseca P, Zhao Q, Zhang P, et al. Patient-derived organoids in precision cancer medicine. Med. 2024;5:1351–77. 39341206 10.1016/j.medj.2024.08.010

[51] Dowler S, Currie RA, Campbell DG, Deak M, Kular G, Downes CP, et al. Identification of pleckstrin-homology-domain-containing proteins with novel phosphoinositide-binding specificities. Biochem J. 2000;351:19–31. 11001876 10.1042/0264-6021:3510019 PMC1221362

[52] Hogan A, Yakubchyk Y, Chabot J, Obagi C, Daher E, Maekawa K, et al. The phosphoinositol 3,4-bisphosphate-binding protein TAPP1 interacts with syntrophins and regulates actin cytoskeletal organization. J Biol Chem. 2004;279:53717–24. 15485858 10.1074/jbc.M410654200

[53] Lauffart B, Gangisetty O, Still IH. Molecular cloning, genomic structure and interactions of the putative breast tumor suppressor TACC2. Genomics. 2003;81:192–201. 12620397 10.1016/s0888-7543(02)00039-3

[54] Shakya M, Zhou A, Dai D, Zhong Q, Zhou Z, Zhang Y, et al. High expression of TACC2 in hepatocellular carcinoma is associated with poor prognosis. Cancer Biomark Sect Dis Markers. 2018;22:611–9. 10.3233/CBM-170091 PMC6130418 29843208

[55] Lin ZR, Xia TL, Wang MY, Zhang LJ, Liu YM, Yuan BY, et al. Inactivation of TACC2 epigenetically represses CDKN1A and confers sensitivity to CDK inhibitors. Med. 2025;6:100568. 10.1016/j.medj.2024.12.002 39793578

[56] Costa R, Carneiro BA, Taxter T, Tavora FA, Kalyan A, Pai SA, et al. FGFR3-TACC3 fusion in solid tumors: mini review. Oncotarget. 2016;7:55924–38. 27409839 10.18632/oncotarget.10482 PMC5342462

[57] Gao Q, Liang WW, Foltz SM, Mutharasu G, Jayasinghe RG, Cao S, et al. Driver fusions and their implications in the development and treatment of human cancers. Cell Rep. 2018;23:227–38.e3. 29617662 10.1016/j.celrep.2018.03.050 PMC5916809

[58] Singh D, Chan JM, Zoppoli P, Niola F, Sullivan R, Castano A, et al. Transforming fusions of FGFR and TACC genes in human glioblastoma. Science. 2012;337:1231–5. 22837387 10.1126/science.1220834 PMC3677224

[59] Brennan C. FGFR-TACC approaches the first turn in the race for targetable GBM mutations. Neuro-Oncol. 2017;19:461–2. 28388710 10.1093/neuonc/nox005 PMC5464322

[60] Lasorella A, Sanson M, Iavarone A. FGFR-TACC gene fusions in human glioma. Neuro-Oncol. 2017;19:475–83. 27852792 10.1093/neuonc/now240 PMC5464372

[61] Shaw AT, Riely GJ, Bang Y-J, Kim D-W, Camidge DR, Solomon BJ, et al. Crizotinib in ROS1-rearranged advanced non-small-cell lung cancer (NSCLC): updated results, including overall survival, from PROFILE 1001. Ann Oncol J Eur Soc Med Oncol. 2019;30:1121–6. 10.1093/annonc/mdz131 PMC6637370 30980071

[62] Drilon A, Siena S, Dziadziuszko R, Barlesi F, Krebs MG, Shaw AT, et al. Entrectinib in ROS1 fusion-positive non-small-cell lung cancer: integrated analysis of three phase 1-2 trials. Lancet Oncol. 2020;21:261–70. 31838015 10.1016/S1470-2045(19)30690-4 PMC7811790

[63] Cocco E, Scaltriti M, Drilon A. NTRK fusion-positive cancers and TRK inhibitor therapy. Nat Rev Clin Oncol. 2018;15:731–47. 30333516 10.1038/s41571-018-0113-0 PMC6419506

[64] Drilon A, Laetsch TW, Kummar S, DuBois SG, Lassen UN, Demetri GD, et al. Efficacy of Larotrectinib in TRK fusion-positive cancers in adults and children. N Engl J Med. 2018;378:731–9. 29466156 10.1056/NEJMoa1714448 PMC5857389

[65] Desai AV, Robinson GW, Gauvain K, Basu EM, Macy ME, Maese L, et al. Entrectinib in children and young adults with solid or primary CNS tumors harboring NTRK, ROS1, or ALK aberrations (STARTRK-NG). Neuro-Oncol. 2022;24:1776–89. 35395680 10.1093/neuonc/noac087 PMC9527518

[66] Fishbein L, Leshchiner I, Walter V, Danilova L, Robertson AG, Johnson AR, et al. Comprehensive molecular characterization of pheochromocytoma and paraganglioma. Cancer Cell. 2017;31:181–93. 28162975 10.1016/j.ccell.2017.01.001 PMC5643159

[67] Liu H, Zhang Z, Han Y, Fan A, Liu H, Zhang X, et al. The FENDRR/FOXC2 Axis Contributes to Multidrug Resistance in Gastric Cancer and Correlates With Poor Prognosis Frontiers in Oncology. 2021;11:634579. 10.3389/fonc.2021.634579 PMC8044876 33869020

[68] P.P.A. Marlies, Leenaars Coenraad F.M., Hendriksen Wim A., de Leeuw Florina, Carat Philippe, Delahaut René, et al. The Production of Polyclonal Antibodies in Laboratory Animals. Alternatives to Laboratory Animals. 1999;27:79–102. 10.1177/026119299902700106 25423403

[69] Nakagawa H, Wang TC, Zukerberg L, Odze R, Togawa K, May GH, et al. The targeting of the cyclin D1 oncogene by an Epstein-Barr virus promoter in transgenic mice causes dysplasia in the tongue, esophagus and forestomach. Oncogene 1997;14:1185–90. 10.1038/sj.onc.1200937 9121767

[70] Jacks T, Remington L, Williams BO, Schmitt EM, Halachmi S, Bronson RT, et al. Tumor spectrum analysis in p53-mutant mice. Curr Biol 1994;4:1–7. 10.1016/s0960-9822(00)00002-6 7922305

