# Supplementary material for: The PLEKHA1-TACC2 fusion gene drives tumorigenesis via vascular mimicry formation in esophageal squamous-cell carcinoma
Source: Cell Death Differ. 2025 Jul 5;32(12):2323–39. doi: 10.1038/s41418-025-01536-1 (PMC12669629; doi:10.1038/s41418-025-01536-1)
Supplement: Supplementary file 1 — Supplementary material [file 41418_2025_1536_MOESM1_ESM.pdf]

Supplemental information

The PLEKHA1-TACC2 fusion gene drives tumorigenesis via vascular mimicry formation in esophageal squamous-cell carcinoma

Supplemental Figures

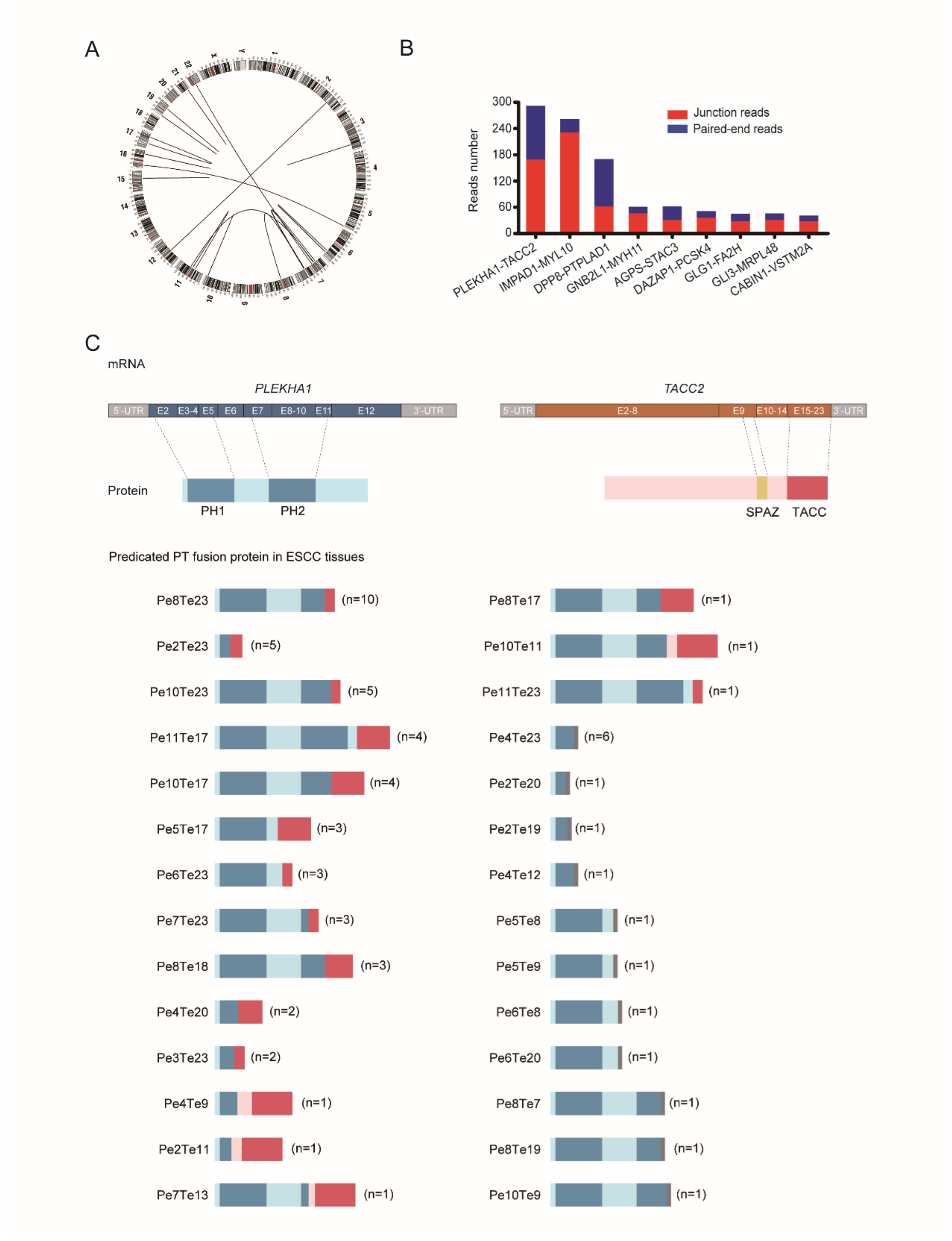

Supplemental Figure 1 Identification of fusion transcripts by RNA-seq and different forms of *PLEKHA1-TACC2* present in ESCC.

A. Circos plot of fusion gene candidates through RNA sequencing from ESCC 1. Chromosome ideograms are shown in the outer layer. Line arcs linking the two genomic

loci indicate fusion transcripts.

B. The number of junction and paired-end reads for the fusion genes, as detected by RNA-seq.

C. Schematic graph of mRNA and protein structures of *PLEKHA1* and *TACC2* genes (top). The mature mRNA transcript of *PLEKHA1* harbours 12 exons, and the translational product is a protein containing N-terminal PH (PH1) (encoded by exon 2-5) and C-terminal PH (PH2) (encoded by exon 7-11) domain. The mature mRNA transcript of *TACC2* harbours 23 exons, and the translational product is a protein containing SPAZ (encoded by exon 9) and TACC (encoded by exon 15-23) domain. UTR, untranslated region; E, exon. Predicted protein structures of the *PLEKHA1-TACC2* transcripts according to Sanger sequencing (bottom). Dark blue parts, PH domain of PLEKHA1; Dark Red parts, TACC domain of TACC2; Grey parts, the sequence with frame shift mutation.

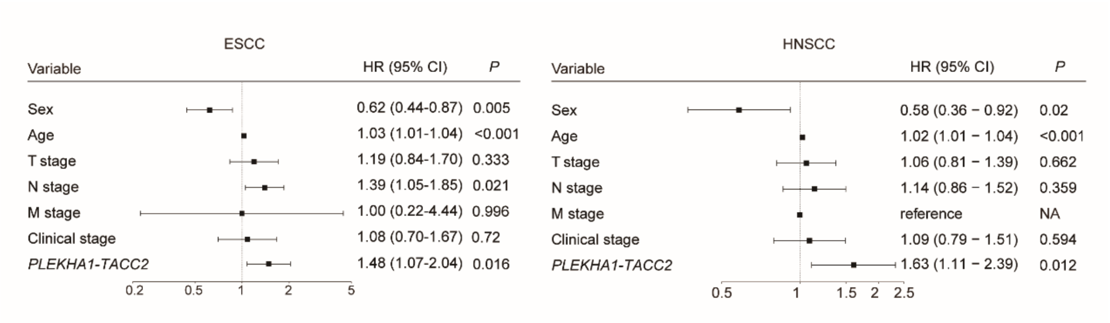

Supplemental Figure 2 *PLEKHA1-TACC2* was positively correlated with unfavourable overall survival in patients with ESCC and HNSCC.

Forest plots show that *PLEKHA1-TACC2* expression could be an independent unfavorable prognostic factor for overall survival in ESCC and HNSCC patients. The 95% confidence interval of hazard ratio (HR) (95% CI) and *P* values were calculated using Cox regression analysis.

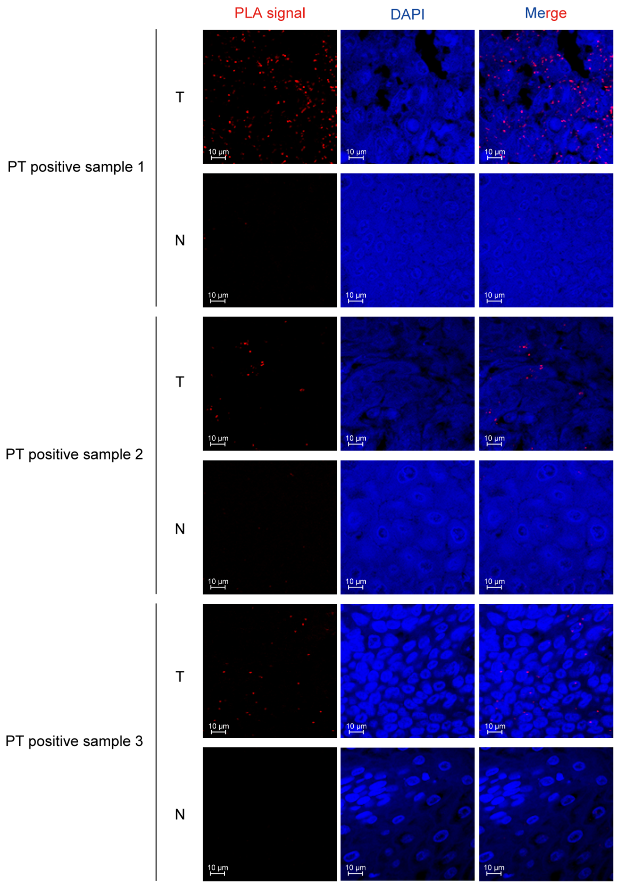

Supplemental Figure 3 PLEKHA1-TAC2 fusion protein was expressed in tumor tissues compared to adjacent normal tissues.

Detection of the endogenous PLEKHA1-TACC2 protein by Duolink PLA assay in ESCC tissues (T) and adjacent normal tissues (N) obtained from patients. Red: positive Duolink PLA fluorescence signals, blue: nuclei. Scale bars: 10 μm.

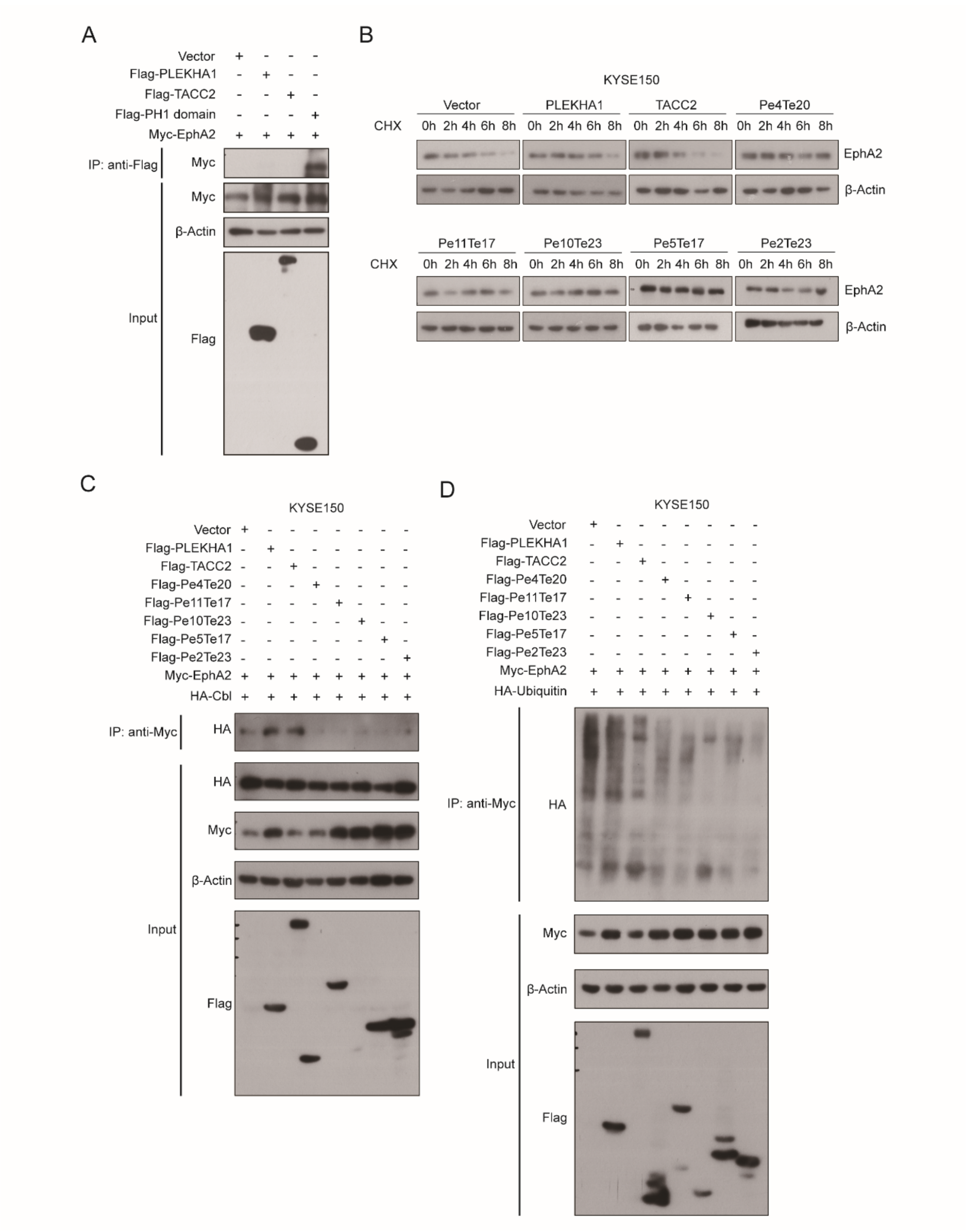

Supplemental Figure 4 PLEKHA1-TACC2 constrains the level of EphA2 ubiquitination through inhibiting the interaction between EphA2 and c-cbl.

A. HEK-293T cells were co-transfected with Myc-tagged EphA2 and Flag-tagged PLEKHA1, TACC2, PH1 domain of PLEKHA1, or an empty control for 48 hours and co-immunoprecipitation assays and immunoblotting analyses with the indicated antibodies

were performed.

B. The expression of EphA2 in KYSE150 cells stably expressing Flag-tagged PLEKHA1, TACC2, Pe4Te20, Pe11Te17, Pe10Te23, Pe5Te17, Pe2Te23, or an empty vector with CHX treatment for indicated duration was determined by the immunoblotting analysis.

C. KYSE150 cells stably expressing Flag-tagged PLEKHA1, TACC2, Pe4Te20, Pe11Te17, Pe10Te23, Pe5Te17, Pe2Te23, or an empty vector were transfected with Myc-tagged EphA2 and HA-cbl then co-immunoprecipitation assays and immunoblotting analyses with the indicated antibodies were performed.

D. KYSE150 cells stably expressing Flag-tagged PLEKHA1, TACC2, Pe4Te20, Pe11Te17, Pe10Te23, Pe5Te17, Pe2Te23, or an empty vector were transfected with Myc-tagged EphA2 and HA-ubiquitin then co-immunoprecipitation assays and immunoblotting analyses with the indicated antibodies were performed.

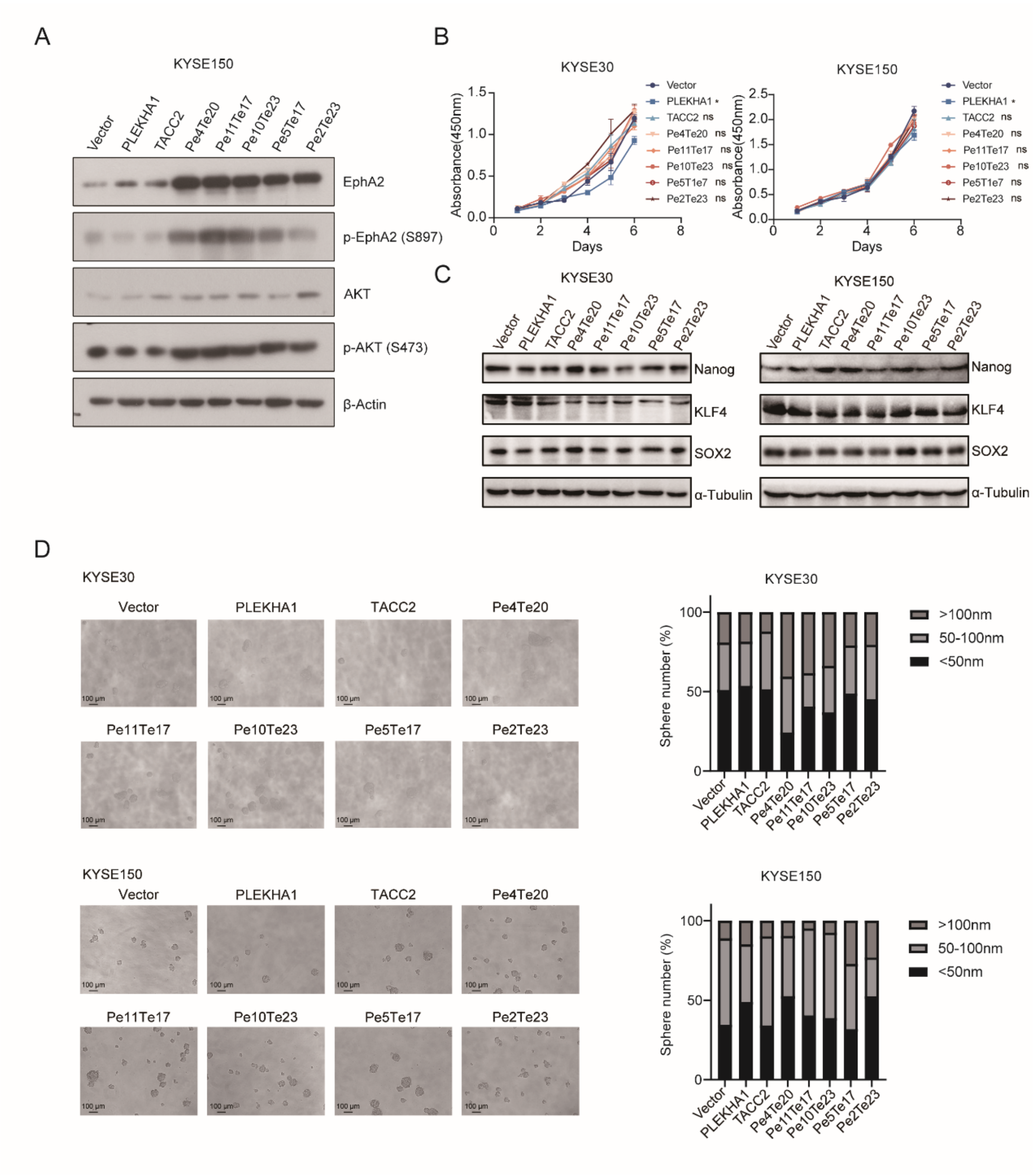

Supplemental Figure 5 PLEKHA1-TACC2 upregulates the EphA2/AKT signalling pathway but not affect cell proliferation.

A. The lysates of KYSE150 cells stably expressing Flag-tagged PLEKHA1, TACC2, Pe4Te20, Pe11Te17, Pe10Te23, Pe5Te17, Pe2Te23, or an empty vector were blotted for total EphA2, p-EphA2 (S897), total AKT and p-AKT (S473).

B. CCK8 assay showing growth curves of KYSE30 (left) and KYSE150 (right) cells stably expressing Flag-tagged PLEKHA1, TACC2, Pe4Te20, Pe11Te17, Pe10Te23, Pe5Te17,

Pe2Te23, or an empty vector. Significance was measured by one-way ANOVA. *$P<0.05$, ns indicates no significance.

C. The expression of Nanog, KLF4 and SOX2 in KYSE30 and KYSE150 cells stably expressing Flag-tagged PLEKHA1, TACC2, Pe4Te20, Pe11Te17, Pe10Te23, Pe5Te17, Pe2Te23, or an empty vector was determined by immunoblotting analyses.

D. Representative images and quantification analysis of sphere formation analysis in KYSE30 and KYSE150 cells stably expressing Flag-tagged PLEKHA1, TACC2, Pe4Te20, Pe11Te17, Pe10Te23, Pe5Te17, Pe2Te23, or an empty vector. Scale bars: 100 μm.

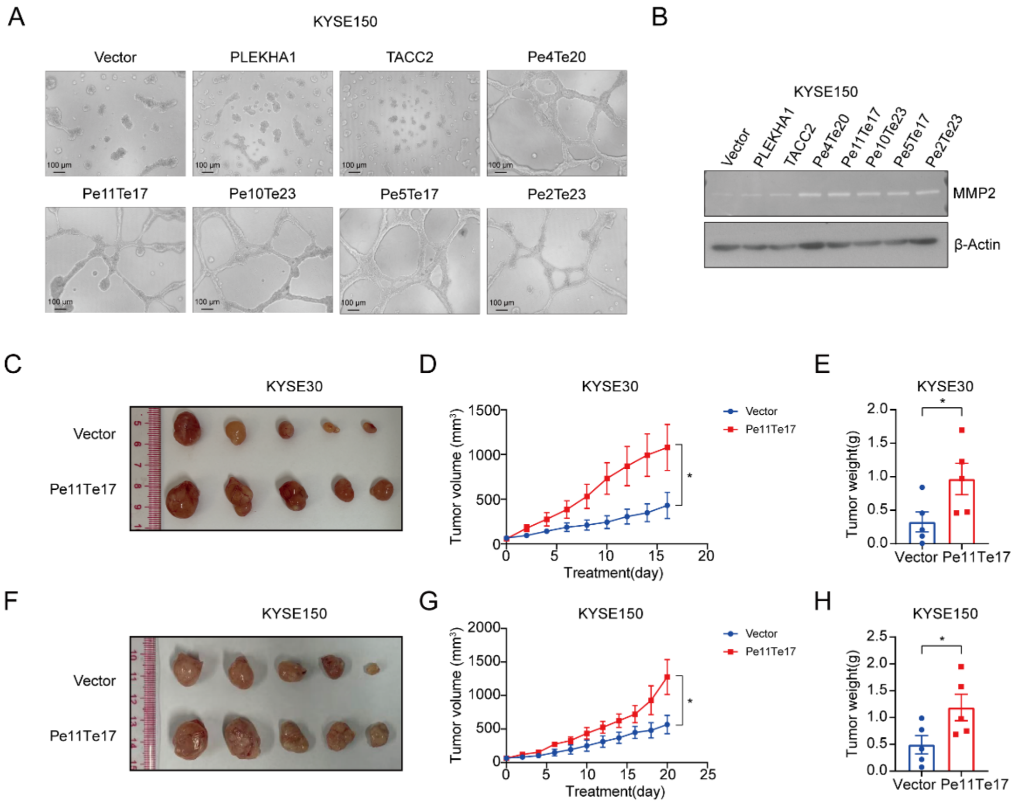

Supplemental Figure 6 PLEKHA1-TACC2 promotes VM formation and MMP2 expression.

A. The VM ability of KYSE150 cells stably expressing Flag-tagged PLEKHA1, TACC2, Pe4Te20, Pe11Te17, Pe10Te23, Pe5Te17, Pe2Te23, or an empty vector was determined by an *in vitro* tube formation assay. Scale bars: 100 μm.

B. The conditioned medium of KYSE150 cells stably expressing Flag-tagged PLEKHA1, TACC2, Pe4Te20, Pe11Te17, Pe10Te23, Pe5Te17, Pe2Te23, or an empty vector was collected for gelatin zymography (top), and the cell lysates of these cells were blotted for β-Actin (bottom).

C. KYSE30 cells stably expressing Flag-tagged Pe11Te17 or an empty vector were subcutaneously injected into the left flanks of athymic nude mice. Representative images of resected tumors from each group of mice at 16 days after injection (n = 5).

D-E. Growth curves (D) and tumor weight (E) in the above-mentioned groups in athymic nude mice bearing KYSE30 tumors ($n = 5$). Data are presented as mean±s.e.m. The P values were calculated using the unpaired t-test.

F. KYSE150 cells stably expressing Flag-tagged Pe11Te17 or an empty vector were subcutaneously injected into the left flanks of athymic nude mice. Representative images of resected tumors from each group of mice at 20 days after injection ($n = 5$).

G-H. Growth curves (G) and tumor weight (H) in the above-mentioned groups in athymic nude mice bearing KYSE150 tumors ($n = 5$). Data are presented as mean±s.e.m. The P values were calculated using the unpaired t-test.

*$P<0.05$, ns indicates no significance.

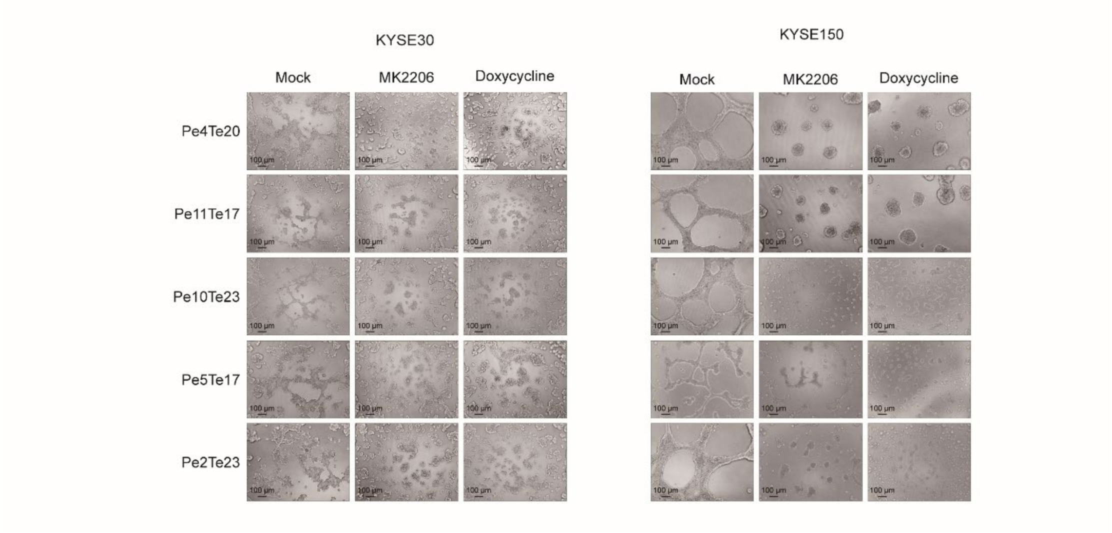

Supplemental Figure 7 MK2206 and Doxycyline inhibit VM formation induced by PLEKHA1-TACC2.

The VM ability of KYSE30 and KYSE150 cells stably expressing Flag-tagged Pe4Te20, Pe11Te17, Pe10Te23, Pe5Te17, Pe2Te23, with indicated treatment was determined by an *in vitro* tube formation assay. Scale bars: 100 μm.

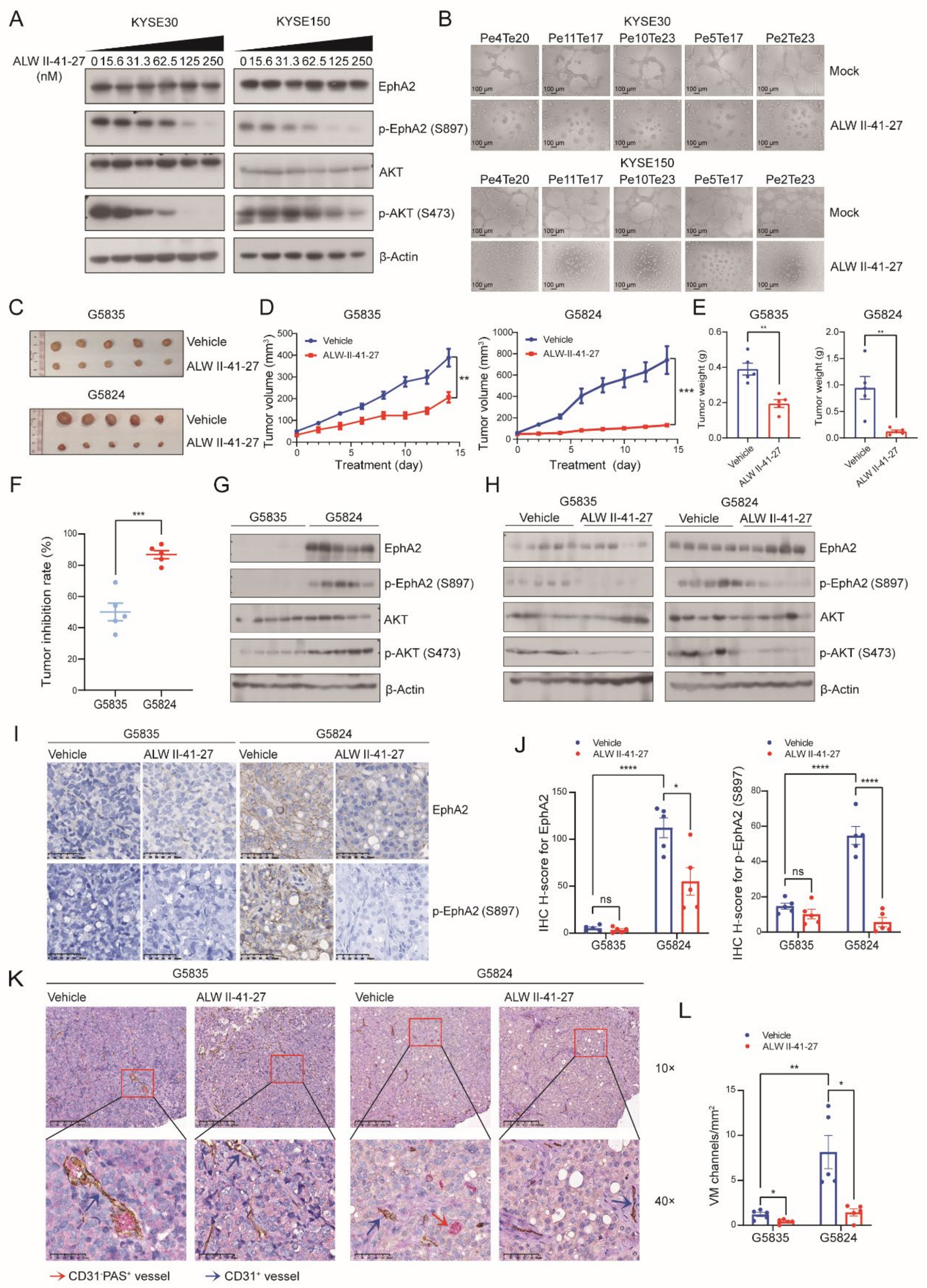

Supplemental Figure 8 ALW II-41-27 inhibits the PLEKHA1-TACC2 activity and suppresses tumor growth.

A. The expression of total EphA2, p-EphA2 (S897), total AKT and p-AKT (S473) in KYSE30

and KYSE150 cells stably expressing Flag-tagged Pe4Te20 treated with different concentrations of ALW II-41-27 for 48 h was determined by immunoblotting analyses.

B. The VM ability of KYSE30 and KYSE150 cells stably expressing Flag-tagged Pe4Te20, Pe11Te17, Pe10Te23, Pe5Te17, Pe2Te23, without and with ALW II-41-27 (125 nM) treatment was determined by an *in vitro* tube formation assay. Scale bars: 100 μm.

C. The images of the tumors obtained from PDX G5835 and G5824 after ALW II-41-27 or vehicle treatment for 14 days (n=5, each subgroup).

D. Growth curve of PDX G5835 (left) and G5824 (right) in mice treated with ALW II-41-27 or vehicle was plotted by measuring the relative tumor volume at indicated day. Data are presented as mean±s.e.m. The *P* values were calculated using the unpaired t-test.

E. Tumor weight of PDX G5835 (left) and G5824 (right) in mice treated with ALW II-41-27 or vehicle were measured at the endpoint of the experiment. Data are presented as mean±s.e.m.

F. Tumor growth inhibition rates of PDX G5835 and G5824 with ALW II-41-27 treatments.

G. The expression of total EphA2, p-EphA2 (S897), total AKT and p-AKT (S473) in PDX G5835 and G5824 with vehicle treatment was determined by immunoblotting analyses.

H. The expression of total EphA2 and p-EphA2 (S897), total AKT and p-AKT (S473) in PDX G5835 and G5824 treated with ALW II-41-27 or vehicle was determined by immunoblotting analyses.

I. The expression of total EphA2 and p-EphA2 (S897) in PDX G5835 and G5824 with ALW II-41-27 or vehicle treatment was determined by IHC. Scale bars were indicated in the images.

J. H-score of EphA2 and p-EphA2 (S897) signal intensity in PDX G5835 and G5824 with ALW II-41-27 or vehicle treatment as calculated by HALO analysis. The *P* values were calculated using one-way ANOVA.

K. Representative images of CD31/PAS/Ter119 staining in tumor tissues of PDX G5835 and G5824 with ALW II-41-27 or vehicle treatment. Red arrows: the tubule-like VM channels. Blue arrows: the blood vessels lined by CD31-positive endothelial cells. Scale bars were indicated in the images.

L. The number of VM channels in PDX G5835 and G5824 with ALW II-41-27 or vehicle treatments as calculated by HALO analysis. The *P* values were calculated using one-way ANOVA.

*P<0.05, **P<0.01, ***P<0.001, ****P<0.0001, ns indicates no significance.

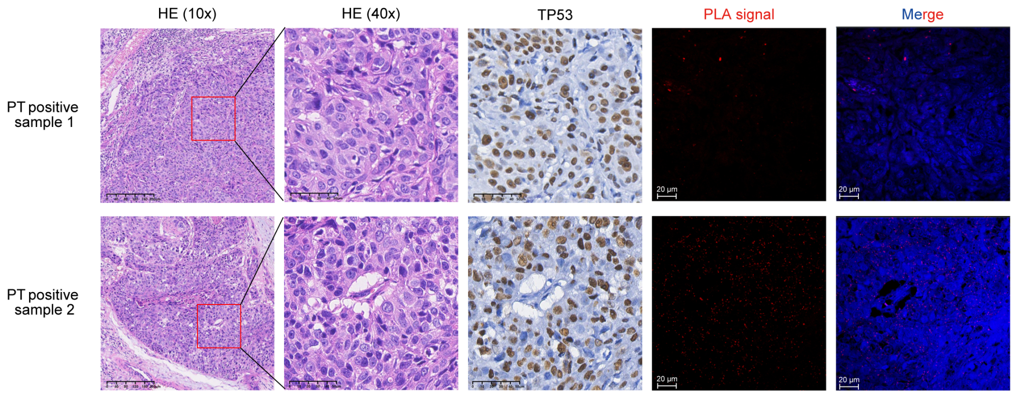

Supplemental Figure 9 Representative images of TP53 and endogenous PLEKHA1-TACC2 protein staining.

Hematoxylin-eosin staining, IHC staining for TP53 and Duolink PLA assay for endogenous PLEKHA1-TACC2 protein detection in serial sections of ESCC tissues. Red: positive Duolink PLA fluorescence signals, blue: nuclei. Scale bars were indicated in the images.

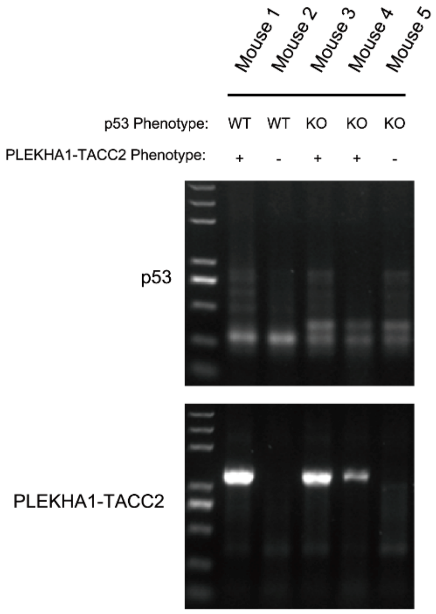

Supplemental Figure 10 Genetic confirmation of transgenic mice.

The presence of p53 mutant (top) and PT fusion (bottom) in mice were identified by PCR. The PCR product size of the wild-type p53 is 288 bp whereas that of the p53-KO is 319 bp. The PCR product size of PT fusion is 1032bp.

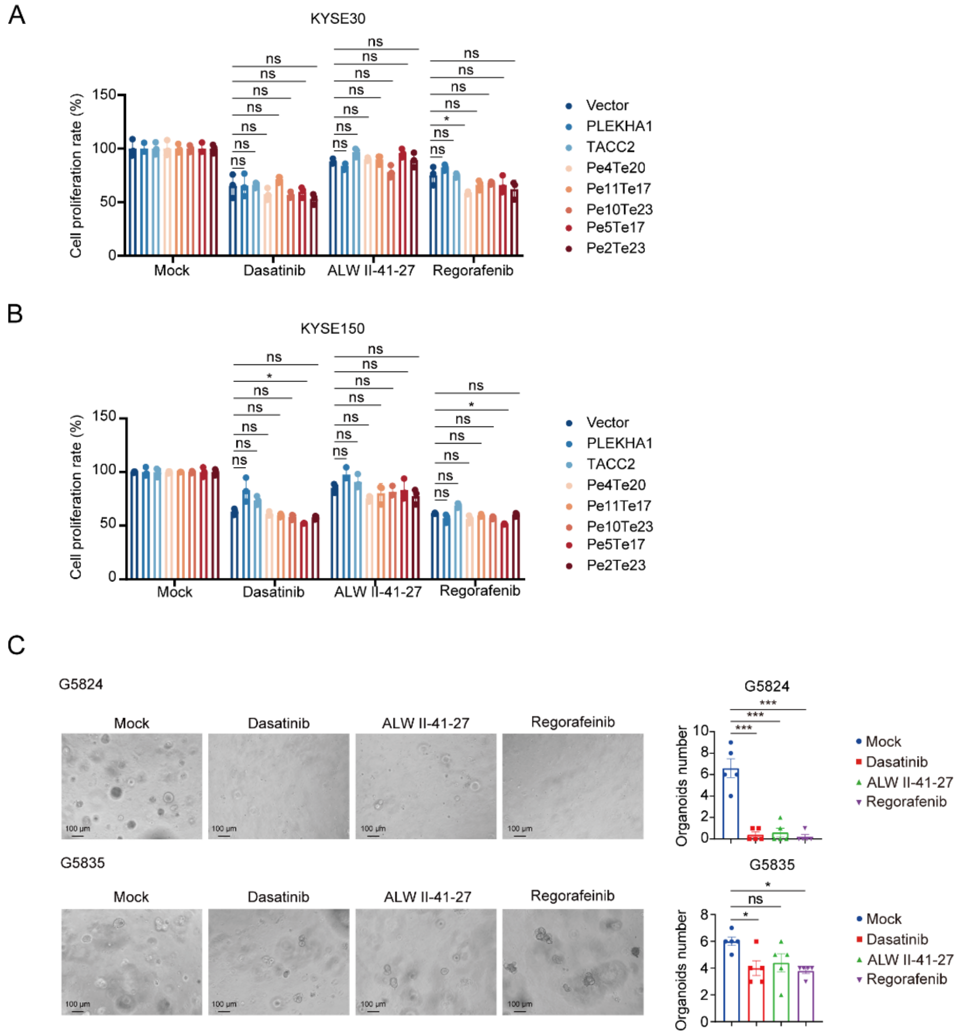

Supplemental Figure 11 EphA2 inhibitors sensitivity assays were performed in ESCC cells lines and PDX derived organoids.

A-B. Cell proliferation rate in KYSE30 (A) and KYSE150 (B) cells stably expressing Flag-tagged PLEKHA1, TACC2, Pe4Te20, Pe11Te17, Pe10Te23, Pe5Te17, Pe2Te23, or an empty vector with mock, dasatinib, ALW II-41-27 or regorafenib treatment.

C. Representative images of the organoids derived from PDX G5835 and G5824 with dasatinib, ALW II-41-27 and regorafenib treatment for 96 h. Scar bar, 100 μm. The *P* values were calculated using one-way ANOVA.

Supplemental Tables

Supplemental Table 1. Baseline characteristics of enrolled ESCC patients used for survival analysis based on *PLEKHA1-TACC2* expression.

**Characteristics**	**n (%) or mean±SD**
**Age (years)**	59.39±9.142
**Gender**	
Male	314 (77.7)
Female	90 (22.3)
**T stage**	
T1	16 (4.0)
T2	76 (18.8)
T3	297 (73.5)
T4	15 (3.7)
**N stage**	
N0	150 (37.3)
N1	174 (43.0)
N2	56 (13.8)
N3	24 (5.9)
**M stage**	
M0	401 (99.3)
M1	3 (0.7)
**Clinical stage (UICC/AJCC 8th edition)**	
I	15 (3.7)
II	174 (43.0)
III	176 (43.7)
IV	39 (9.6)
***PLEKHA1-TACC2* expression**	
Negative	338 (83.7)
Positive	66 (16.3)
***TP53* status**	
Wild type	74 (18.3)
Mutant	72 (17.8)
Untested	258 (63.9)

Data are presented as n (%) or mean±SD.

Supplemental Table 2. Baseline characteristics of enrolled HNSCC patients used for survival analysis based on *PLEKHA1-TACC2* expression.

Characteristics	n (%) or mean±SD
**Age (years)**	56.08±11.408
**Gender**	
Male	325 (80.8)
Female	77 (19.2)
**T stage**	
Tis	4 (1.0)
T1	94 (23.4)
T2	140 (34.8)
T3	86 (21.4)
T4	77 (19.2)
NA	1 (0.2)
**N stage**	
N0	253 (62.9)
N1	66 (16.4)
N2	81 (20.1)
N3	1 (0.3)
NA	1 (0.3)
**M stage**	
M0	401 (99.8)
NA	1 (0.2)
**Clinical stage (UICC/AJCC 8th edition)**	
0	4 (1.0)
I	77 (19.2)
II	85 (21.1)
III	96 (23.9)
IV	139 (34.6)
NA	1 (0.2)
***PLEKHA1-TACC2* expression**	
Negative	344 (85.6)
Positive	58 (14.4)
***TP53* status**	
Wild type	48 (11.9)
Mutant	30 (7.5)
Untested	324 (80.6)

Data are presented as n (%) or mean±SD.

Supplemental Table 3. Baseline characteristics of enrolled ESCC patients used for survival analysis based on *PLEKHA1-TACC2* expression and *TP53* status.

**Characteristics**	**n (%) or mean±SD**
**Age (years)**	59.31±9.221
**Gender**	
Male	110 (75.3)
Female	36 (24.7)
**T stage**	
T1	8 (5.5)
T2	24 (16.4)
T3	111 (76.0)
T4	3 (2.1)
**N stage**	
N0	59 (40.4)
N1	59 (40.4)
N2	21 (14.4)
N3	7 (4.8)
**M stage**	
M0	144 (98.6)
M1	2 (1.4)
**Clinical stage (UICC/AJCC 8th edition)**	
I	8 (5.4)
II	63 (43.2)
III	63 (43.2)
IV	12 (8.2)
***PLEKHA1-TACC2* expression**	
Negative	100 (68.5)
Positive	46 (31.5)
***TP53* status**	
Wild type	74 (50.7)
Missense mutant	62 (42.5)
Frame shift mutant	7 (4.8)
Non-sense mutant	3 (2.0)

Data are presented as n (%) or mean±SD.

Supplemental Table 4. Baseline characteristics of enrolled HNSCC patients used for survival analysis based on *PLEKHA1-TACC2* expression and *TP53* status.

**Characteristics**	**n (%) or mean±SD**
**Age (years)**	53.72±12.830
**Gender**	
Male	67 (85.9)
Female	11 (14.1)
**T stage**	
T1	11 (14.1)
T2	25 (32.1)
T3	23 (29.5)
T4	19 (24.3)
**N stage**	
N0	48 (61.6)
N1	21 (26.8)
N2	8 (10.3)
N3	1 (1.3)
**M stage**	
M0	78 (100)
**Clinical stage (UICC/AJCC 8th edition)**	
I	11 (14.1)
II	14 (17.9)
III	26 (33.4)
IV	27 (34.6)
***PLEKHA1-TACC2* expression**	
Negative	58 (74.5)
Positive	20 (25.5)
***TP53* status**	
Wild type	48 (61.5)
Missense mutant	28 (35.9)
Frame shift mutant	1 (1.3)
Non-sense mutant	1 (1.3)

Data are presented as n (%) or mean±SD.

Supplemental Table 5. Sequences of primers of DNA constructs.

Sequence Name	Sequence
pHAGE-FLAG-PLEKHA1-F	CCTCCATAGAAGACACCGATGGATTACAAGGATGACGACGATAAGATGCCTTATGTGGATCGTCAGAAT
pHAGE-FLAG-PLEKHA1-R	GGGGAGGGATCCTCTAGATCACACGTCACTGACCGGAAGGCT
pHAGE-FLAG-TACC2-F	CCTCCATAGAAGACACCGATGGATTACAAGGATGACGACGATAAGATGGGCAATGAGAACAGCACCTCG
pHAGE-FLAG-TACC2-R	GGGGAGGGATCCTCTAGATTAGCTTTTCCCCATTTTGGCAAT
pHAGE-FLAG-Pe2Te23-F1	CCTCCATAGAAGACACCGATGGATTACAAGGATGACGACGATAAGATGCCTTATGTGGATCGTCAGAAT
pHAGE-FLAG-Pe2Te23-R1	TTCTTCTATTTCTTTATTCTGTGGATTATCCATGTA
pHAGE-FLAG-Pe2Te23-F2	TACATGGATAATCCACAGAATAAAGAAATAGAAGAA
pHAGE-FLAG-Pe2Te23-R2	GGGGAGGGATCCTCTAGATTAGCTTTTCCCCATTTTGGCAAT
pHAGE-FLAG-Pe4Te20-F1	CCTCCATAGAAGACACCGATGGATTACAAGGATGACGACGATAAGATGCCTTATGTGGATCGTCAGAAT
pHAGE-FLAG-Pe4Te20-R1	TCTCTCTCTGTTCGTCCTCAAAACAGAACTCCGCCT
pHAGE-FLAG-Pe4Te20-F2	AGGCGGAGTTCTGTTTTGAGGACGAACAGAGAGAGA
pHAGE-FLAG-Pe4Te20-R2	GGGGAGGGATCCTCTAGATTAGCTTTTCCCCATTTTGGCAAT
pHAGE-FLAG-Pe11Te17-F1	CCTCCATAGAAGACACCGATGGATTACAAGGATGACGACGATAAGATGCCTTATGTGGATCGTCAGAAT
pHAGE-FLAG-Pe11Te17-R1	TGGGTGAGCAGCCTCTCTAGAAGACGCAGATCTGCC
pHAGE-FLAG-Pe11Te17-F2	GGCAGATCTGCGTCTTCTAGAGAGGCTGCTCACCCA
pHAGE-FLAG-Pe11Te17-R2	GGGGAGGGATCCTCTAGATTAGCTTTTCCCCATTTTGGCAAT
pHAGE-FLAG-Pe10Te23-F1	CCTCCATAGAAGACACCGATGGATTACAAGGATGACGACGATAAGATGCCTTATGTGGATCGTCAGAAT
pHAGE-FLAG-Pe10Te23-R1	TTCTTCTATTTCTTTATTCTGCACATAGAAAGTTCG
pHAGE-FLAG-Pe10Te23-F2	CGAACTTTCTATGTGCAGAATAAAGAAATAGAAGAA
pHAGE-FLAG-Pe10Te23-R2	GGGGAGGGATCCTCTAGATTAGCTTTTCCCCATTTTGGCAAT
pHAGE-FLAG-Pe5Te17-F1	CCTCCATAGAAGACACCGATGGATTACAAGGATGACGACGATAAGATGCCTTATGTGGATCGTCAGAAT
pHAGE-FLAG-Pe5Te17-R1	TGGGTGAGCAGCCTCTCTTGTAATTTTTATAGCTTT

pHAGE-FLAG-Pe5Te17-F2	AAAGCTATAAAAATTACAAGAGAGGCTGCTCACCCA
pHAGE-FLAG-Pe5Te17-R2	GGGGAGGGATCCTCTAGATTAGCTTTTCCCCATTTTGGCAAT
pHAGE-MYC-EPHA2-F	CCTCCATAGAAGACACCGATGGAACAAAAACTCATCTCAGAAGAGGATCTGATGGAGCTCCAGGCAGCCCGCGCC
pHAGE-MYC-EPHA2-R	GGGGAGGGATCCTCTAGATCAGATGGGGATCCCCACAGTGTT

175

Supplemental Table 6. Sequences of primers of transgenic mice validation.

Sequence Name	Sequence
Pe4Te20-F	ACACACTCCCTTAGCCACAC
Pe4Te20-R	GCCTCGAACCTGAGCAATCT
p53-F	CACAAAAACAGGTTAAACCCAG
p53-R	AGCACATAGGAGGCAGAGAC
