# Supplementary material for: The PLEKHA1-TACC2 fusion gene drives tumorigenesis via vascular mimicry formation in esophageal squamous-cell carcinoma
Source: Cell Death Differ. 2025 Jul 5;32(12):2323–39. doi: 10.1038/s41418-025-01536-1 (PMC12669629; doi:10.1038/s41418-025-01536-1)
Supplement: Supplementary file 3 — Westwen blotting data [file 41418_2025_1536_MOESM3_ESM.pdf]

Figure 1G

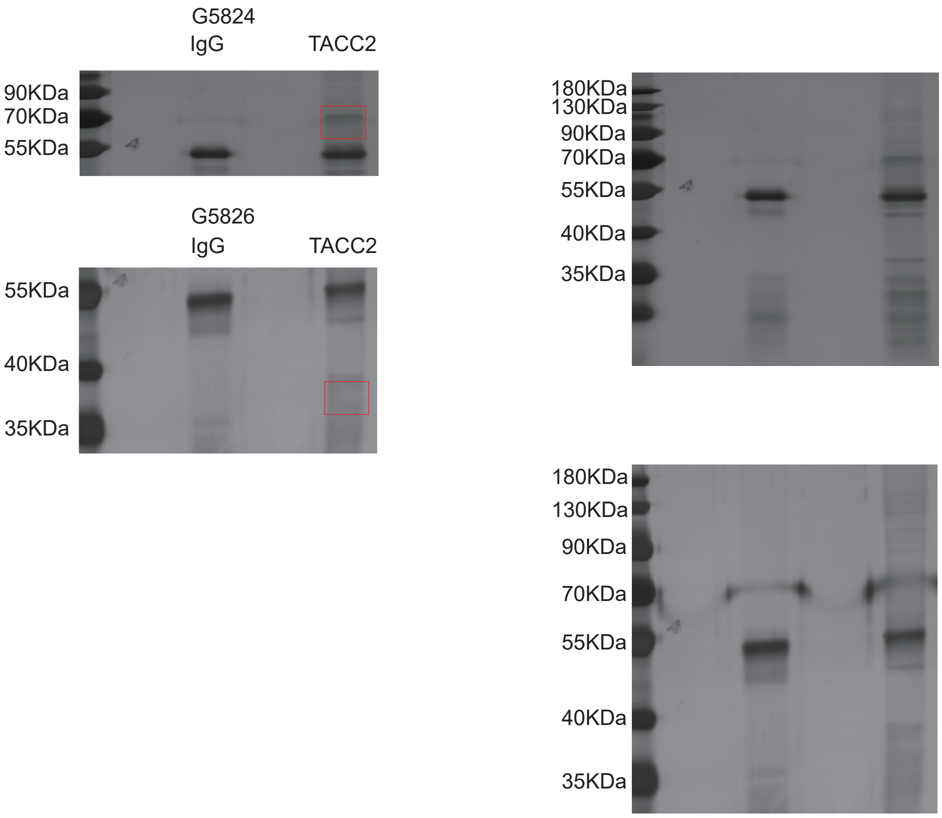
G5824
IgG
TACC2
90KDa
70KDa
55KDa
G5826
IgG
TACC2
55KDa
40KDa
35KDa
180KDa
130KDa
90KDa
70KDa
55KDa
40KDa
35KDa
180KDa
130KDa
90KDa
70KDa
55KDa
40KDa
35KDa

Figure 2A

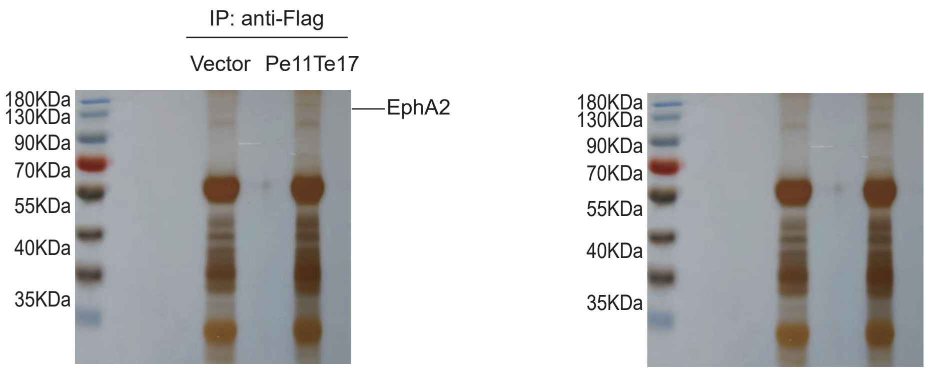

IP: anti-Flag
Vector
Pe11Te17
180KDa
130KDa
90KDa
70KDa
55KDa
40KDa
35KDa
EphA2
180KDa
130KDa
90KDa
70KDa
55KDa
40KDa
35KDa

Figure 2B

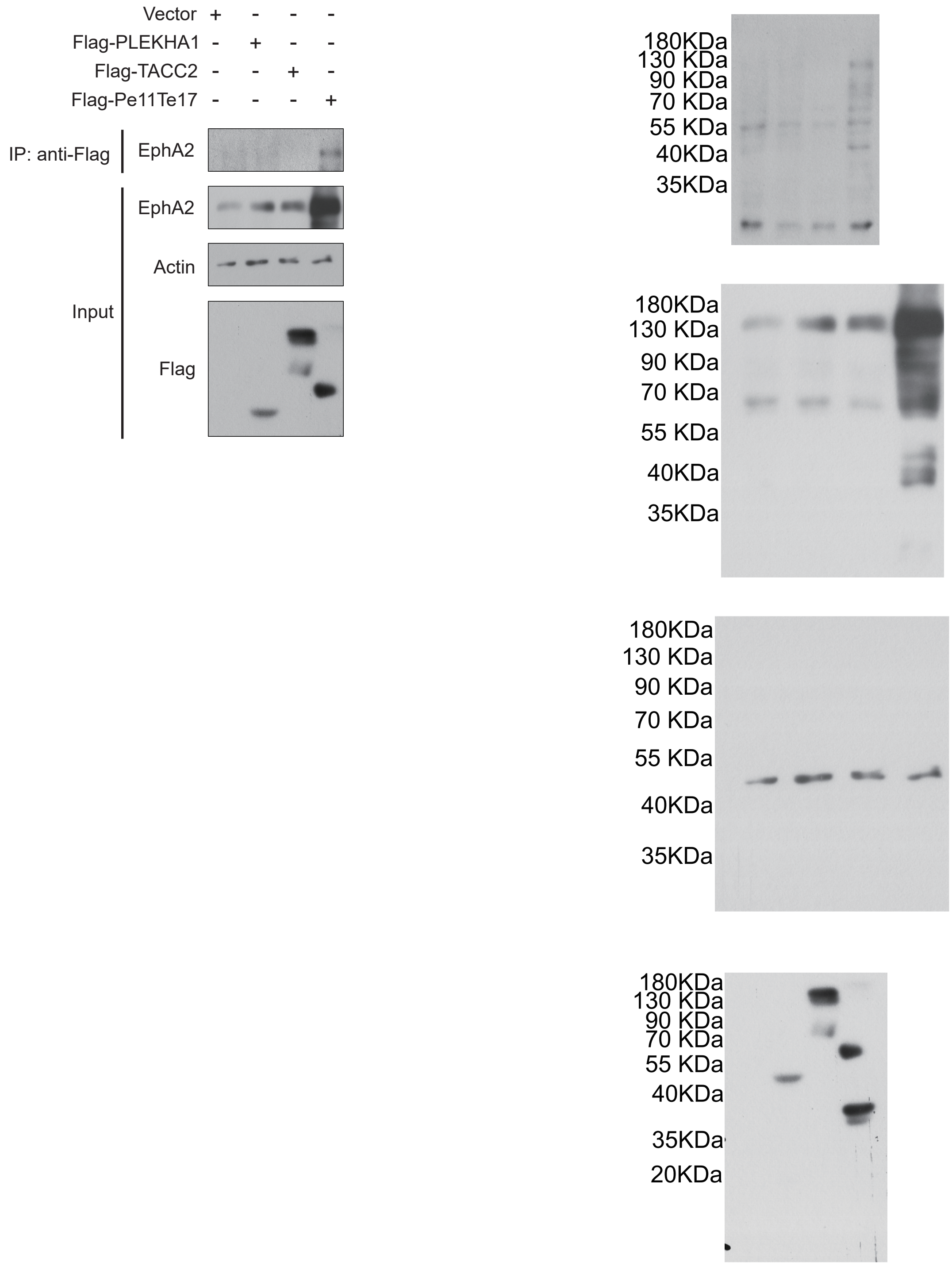

Vector + - - -
Flag-PLEKHA1 - + - -
Flag-TACC2 - - + -
Flag-Pe11Te17 - - - +
IP: anti-Flag
EphA2
EphA2
Actin
Input
Flag
180KDa
130 KDa
90 KDa
70 KDa
55 KDa
40KDa
35KDa
180KDa
130 KDa
90 KDa
70 KDa
55 KDa
40KDa
35KDa
180KDa
130 KDa
90 KDa
70 KDa
55 KDa
40KDa
35KDa
180KDa
130 KDa
90 KDa
70 KDa
55 KDa
40KDa
35KDa
20KDa

Figure 2C

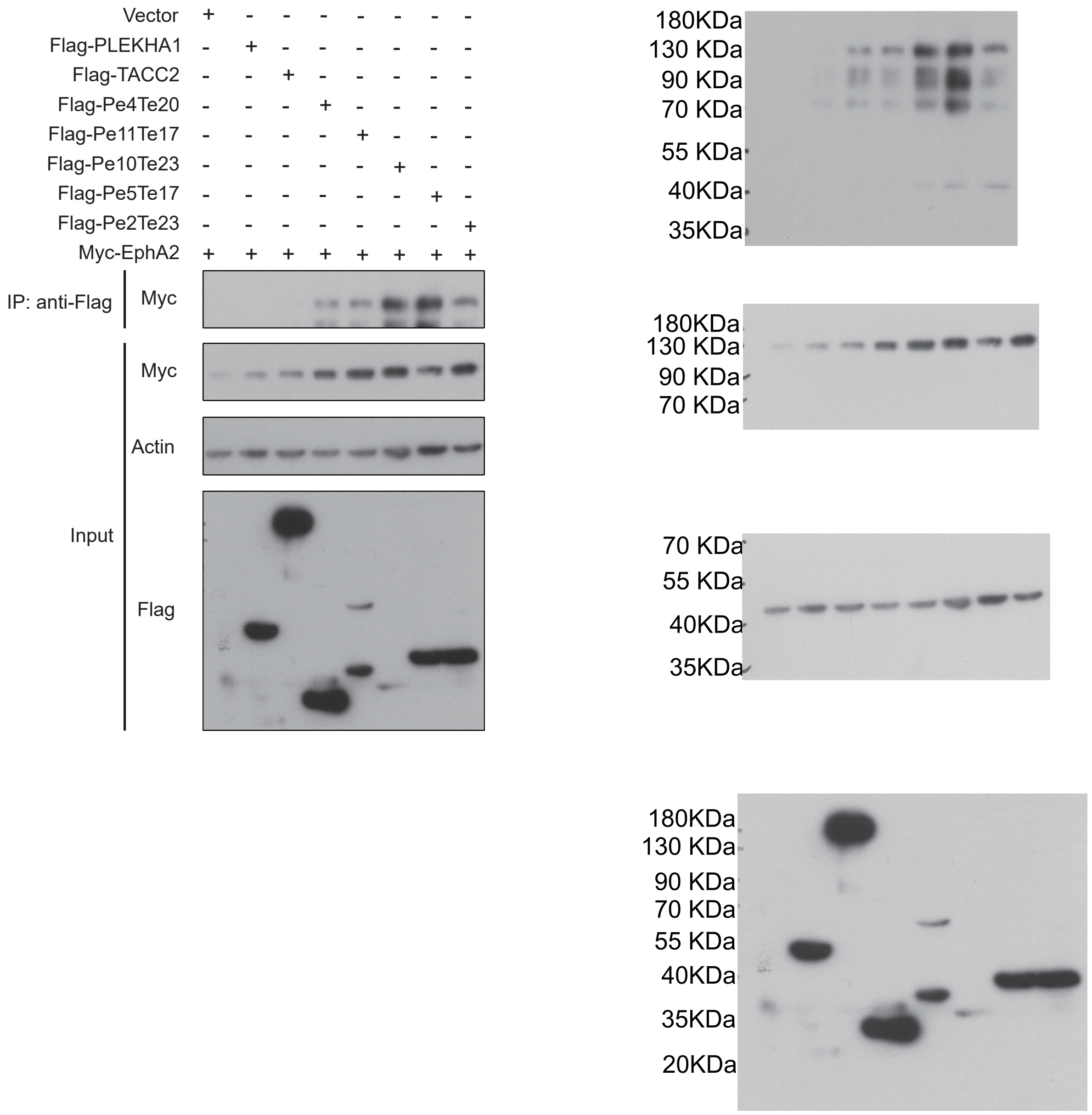
Vector + - - - - - - -
Flag-PLEKHA1 - + - - - - - -
Flag-TACC2 - - + - - - - -
Flag-Pe4Te20 - - - + - - - -
Flag-Pe11Te17 - - - - + - - -
Flag-Pe10Te23 - - - - - + - -
Flag-Pe5Te17 - - - - - - + -
Flag-Pe2Te23 - - - - - - - +
Myc-EphA2 + + + + + + + +
IP: anti-Flag
Myc
Input
Myc
Actin
Flag
180KDa
130 KDa
90 KDa
70 KDa
55 KDa
40KDa
35KDa
180KDa
130 KDa
90 KDa
70 KDa
70 KDa
55 KDa
40KDa
35KDa
180KDa
130 KDa
90 KDa
70 KDa
55 KDa
40KDa
35KDa
20KDa

Figure 2D

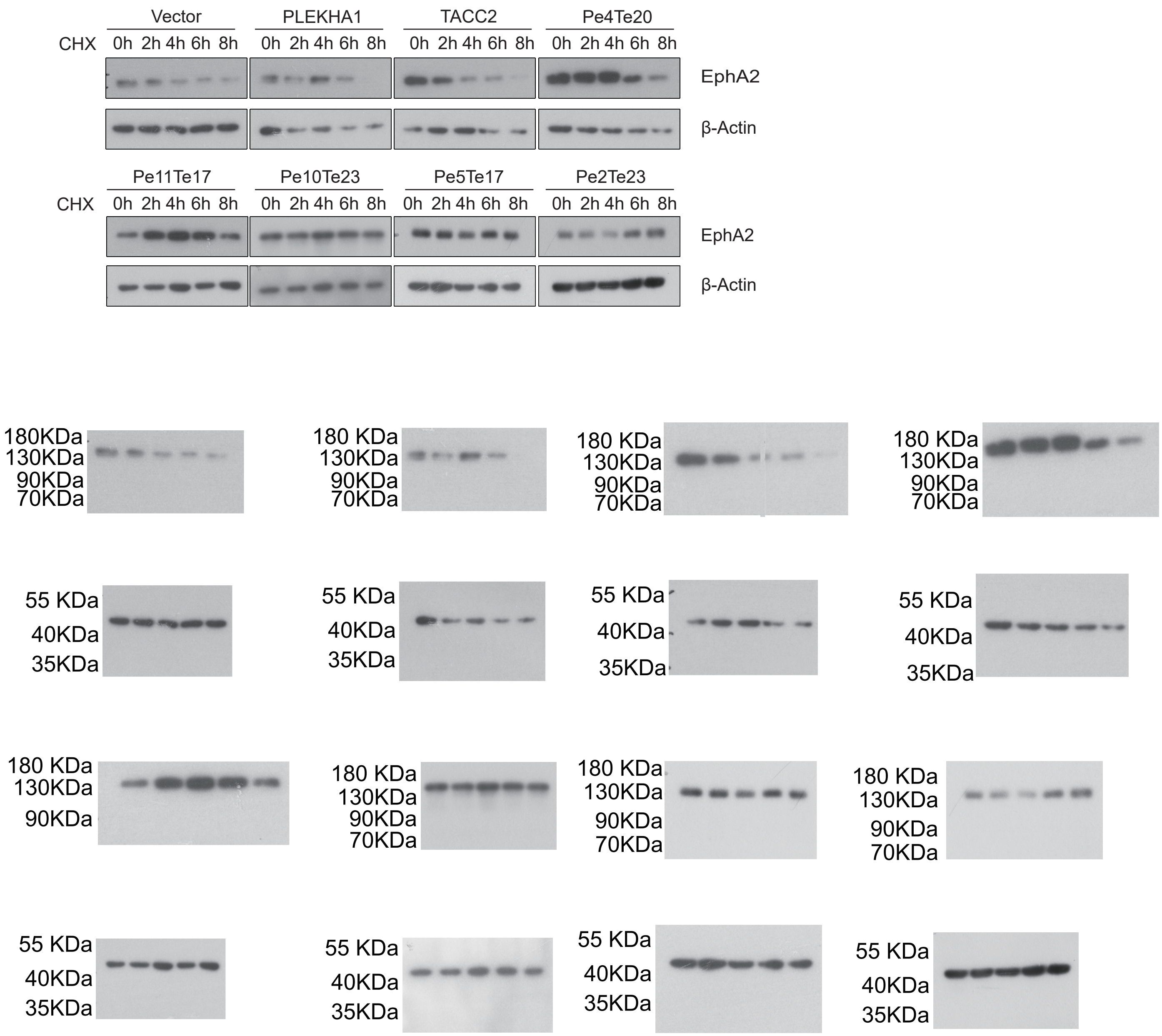
Vector
PLEKHA1
TACC2
Pe4Te20
CHX 0h 2h 4h 6h 8h 0h 2h 4h 6h 8h 0h 2h 4h 6h 8h 0h 2h 4h 6h 8h
EphA2
β-Actin
Pe11Te17
Pe10Te23
Pe5Te17
Pe2Te23
CHX 0h 2h 4h 6h 8h 0h 2h 4h 6h 8h 0h 2h 4h 6h 8h 0h 2h 4h 6h 8h
EphA2
β-Actin
180KDa
130KDa
90KDa
70KDa
180 KDa
130KDa
90KDa
70KDa
180 KDa
130KDa
90KDa
70KDa
180 KDa
130KDa
90KDa
70KDa
55 KDa
40KDa
35KDa
55 KDa
40KDa
35KDa
55 KDa
40KDa
35KDa
55 KDa
40KDa
35KDa
180 KDa
130KDa
90KDa
180 KDa
130KDa
90KDa
70KDa
180 KDa
130KDa
90KDa
70KDa
180 KDa
130KDa
90KDa
70KDa
55 KDa
40KDa
35KDa
55 KDa
40KDa
35KDa
55 KDa
40KDa
35KDa
55 KDa
40KDa
35KDa

Figure 2E

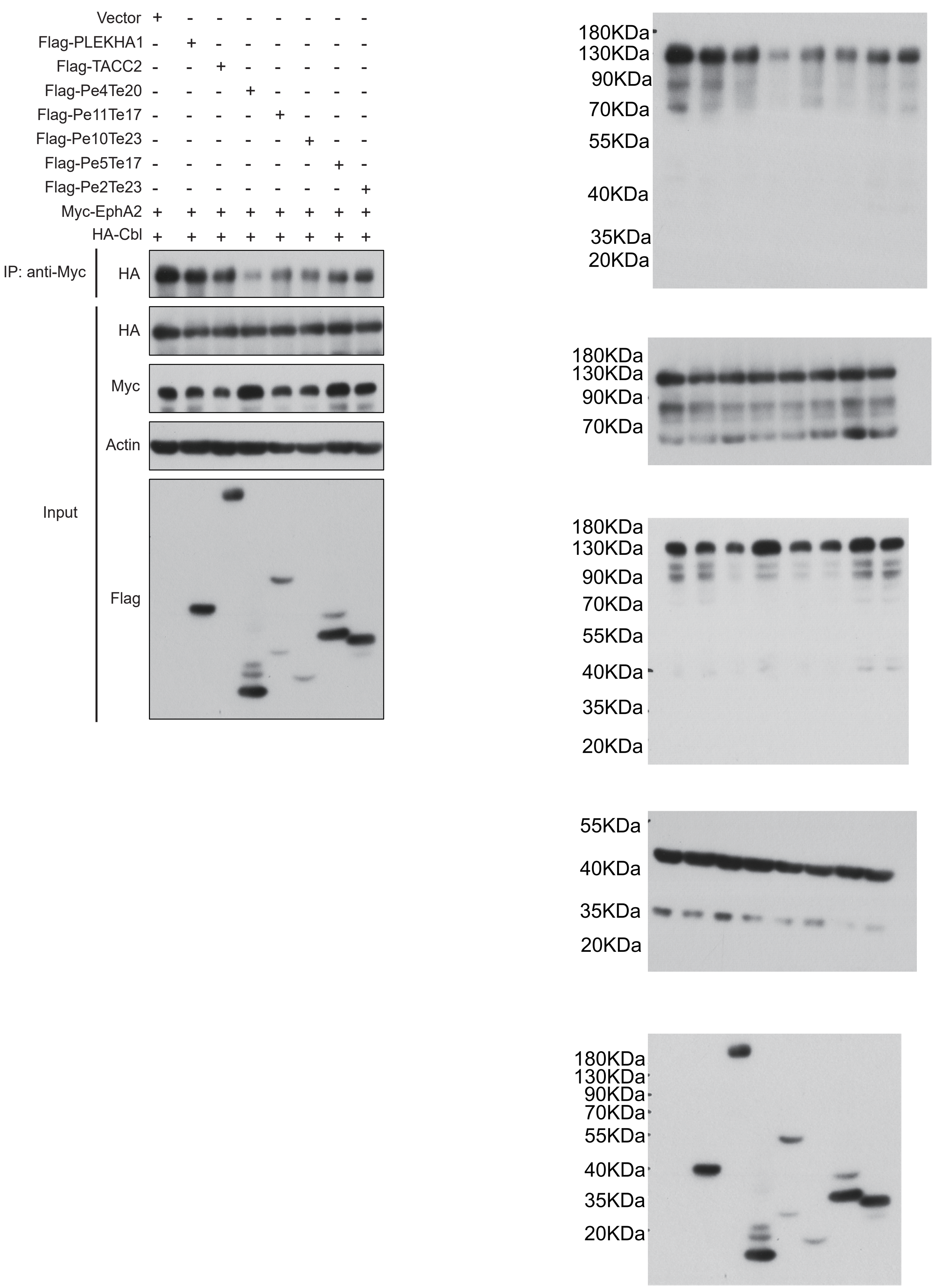
Vector + - - - - - - -
Flag-PLEKHA1 - + - - - - - -
Flag-TACC2 - - + - - - - -
Flag-Pe4Te20 - - - + - - - -
Flag-Pe11Te17 - - - - + - - -
Flag-Pe10Te23 - - - - - + - -
Flag-Pe5Te17 - - - - - - + -
Flag-Pe2Te23 - - - - - - - +
Myc-EphA2 + + + + + + + +
HA-Cbl + + + + + + + +
IP: anti-Myc
HA
HA
Myc
Actin
Input
Flag
180KDa
130KDa
90KDa
70KDa
55KDa
40KDa
35KDa
20KDa
180KDa
130KDa
90KDa
70KDa
180KDa
130KDa
90KDa
70KDa
55KDa
40KDa
35KDa
20KDa
55KDa
40KDa
35KDa
20KDa
180KDa
130KDa
90KDa
70KDa
55KDa
40KDa
35KDa
20KDa

Figure 2F

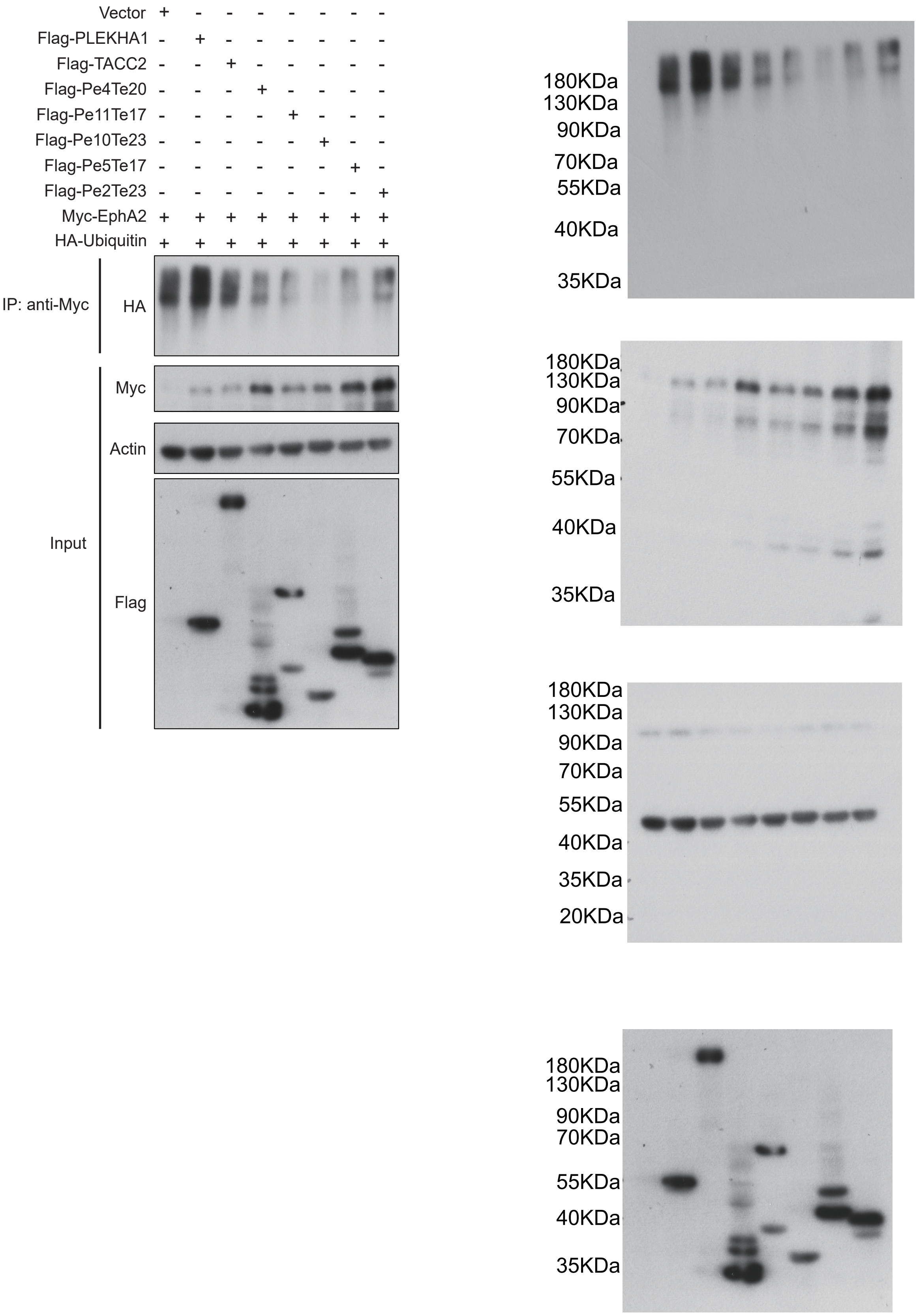
Vector + - - - - - - -
Flag-PLEKHA1 - + - - - - - -
Flag-TACC2 - - + - - - - -
Flag-Pe4Te20 - - - + - - - -
Flag-Pe11Te17 - - - - + - - -
Flag-Pe10Te23 - - - - - + - -
Flag-Pe5Te17 - - - - - - + -
Flag-Pe2Te23 - - - - - - - +
Myc-EphA2 + + + + + + + +
HA-Ubiquitin + + + + + + + +
IP: anti-Myc
HA
Input
Myc
Actin
Flag
180KDa
130KDa
90KDa
70KDa
55KDa
40KDa
35KDa
180KDa
130KDa
90KDa
70KDa
55KDa
40KDa
35KDa
180KDa
130KDa
90KDa
70KDa
55KDa
40KDa
35KDa
20KDa
180KDa
130KDa
90KDa
70KDa
55KDa
40KDa
35KDa

Figure 3A

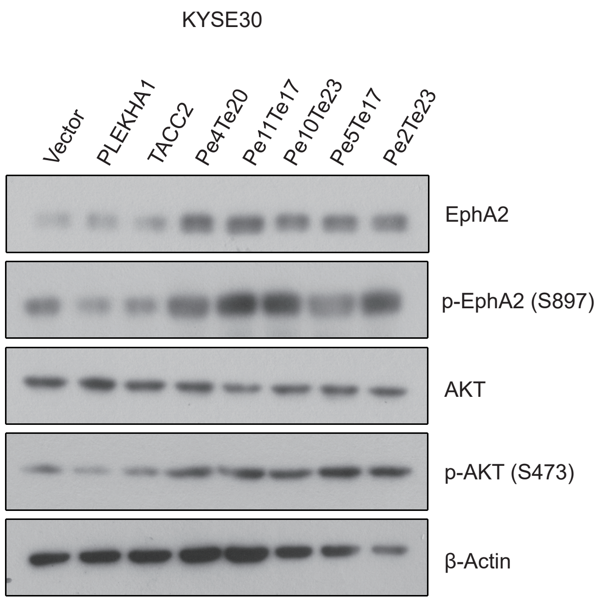
KYSE30
Vector
PLEKHA1
TACC2
Pe4Te20
Pe11Te17
Pe10Te23
Pe5Te17
Pe2Te23
EphA2
p-EphA2 (S897)
AKT
p-AKT (S473)
β-Actin

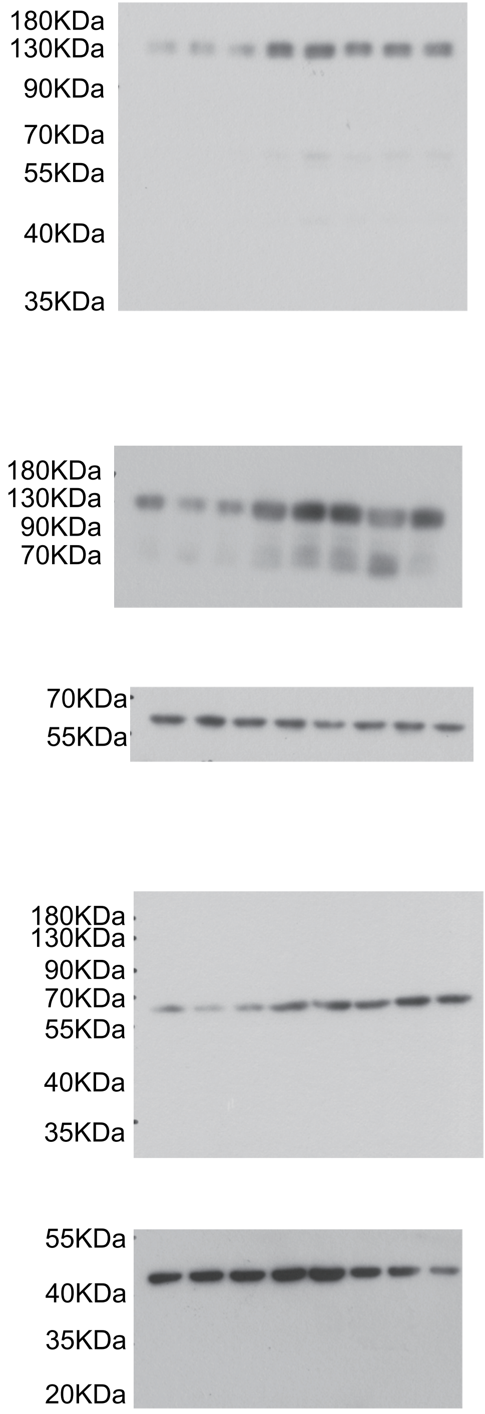
180KDa
130KDa
90KDa
70KDa
55KDa
40KDa
35KDa
180KDa
130KDa
90KDa
70KDa
70KDa
55KDa
180KDa
130KDa
90KDa
70KDa
55KDa
40KDa
35KDa
55KDa
40KDa
35KDa
20KDa

Figure 3C

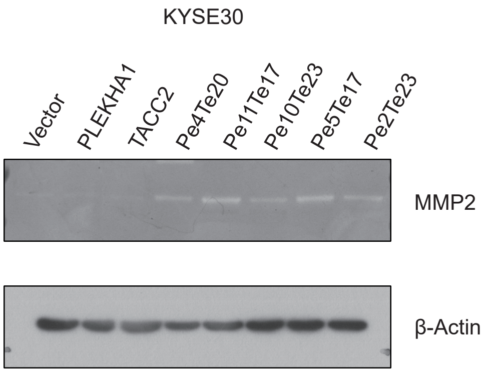
KYSE30
Vector
PLEKHA1
TACC2
Pe4Te20
Pe11Te17
Pe10Te23
Pe5Te17
Pe2Te23
MMP2
β-Actin

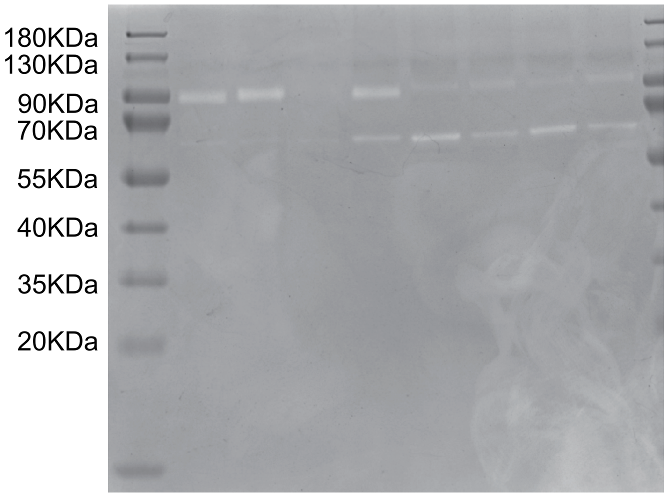
180KDa
130KDa
90KDa
70KDa
55KDa
40KDa
35KDa
20KDa

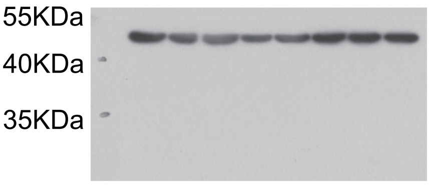
55KDa
40KDa
35KDa

Figure 4A

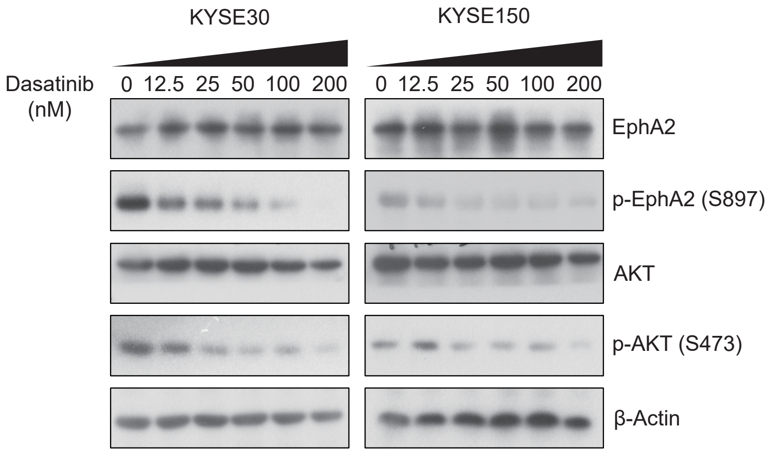
KYSE30
KYSE150
Dasatinib (nM)
0 12.5 25 50 100 200
0 12.5 25 50 100 200
EphA2
p-EphA2 (S897)
AKT
p-AKT (S473)
β-Actin

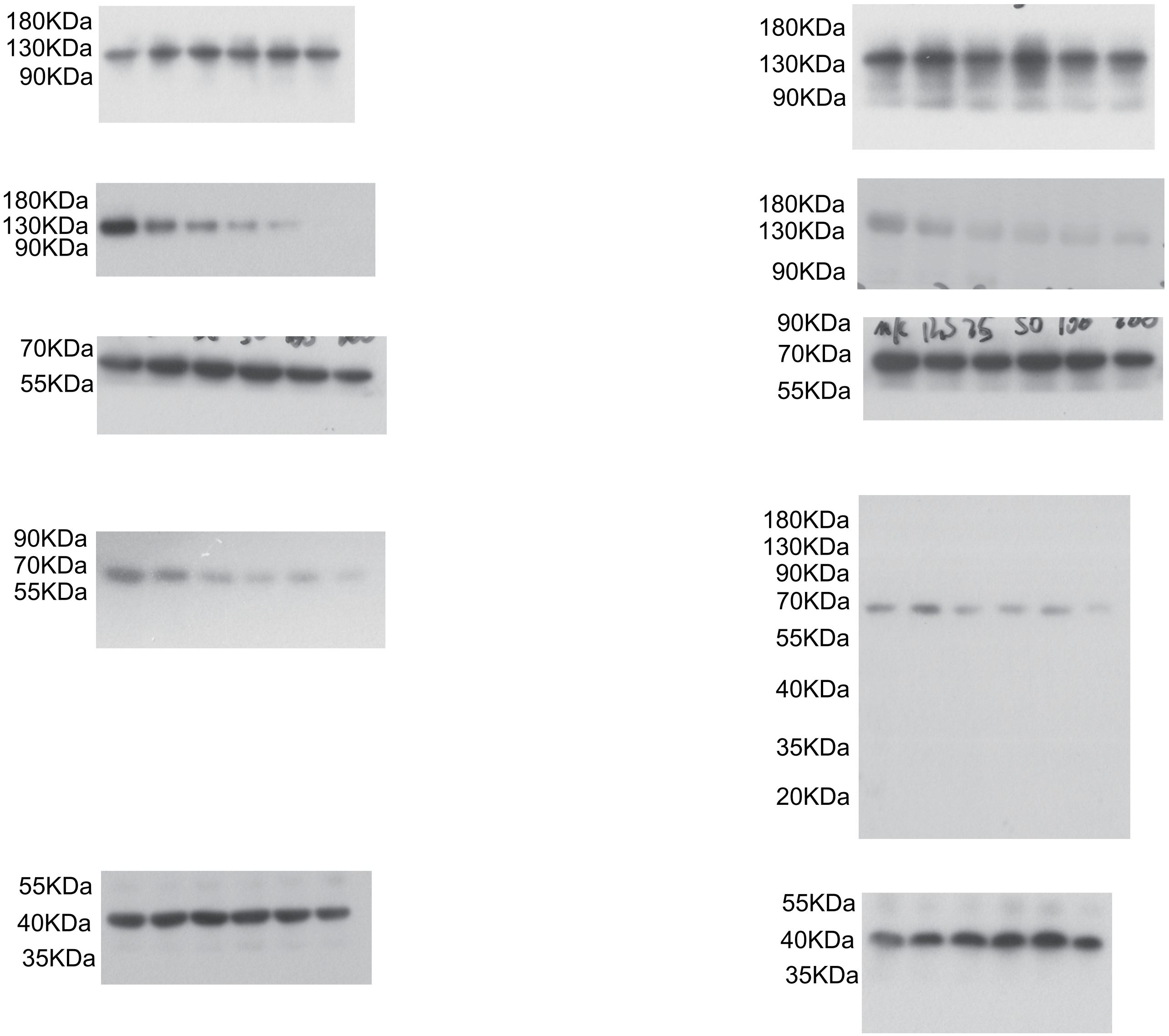
180KDa
130KDa
90KDa
180KDa
130KDa
90KDa
180KDa
130KDa
90KDa
180KDa
130KDa
90KDa
70KDa
55KDa
90KDa
70KDa
55KDa
90KDa
70KDa
55KDa
180KDa
130KDa
90KDa
70KDa
55KDa
40KDa
35KDa
20KDa
55KDa
40KDa
35KDa
55KDa
40KDa
35KDa

Figure 4G

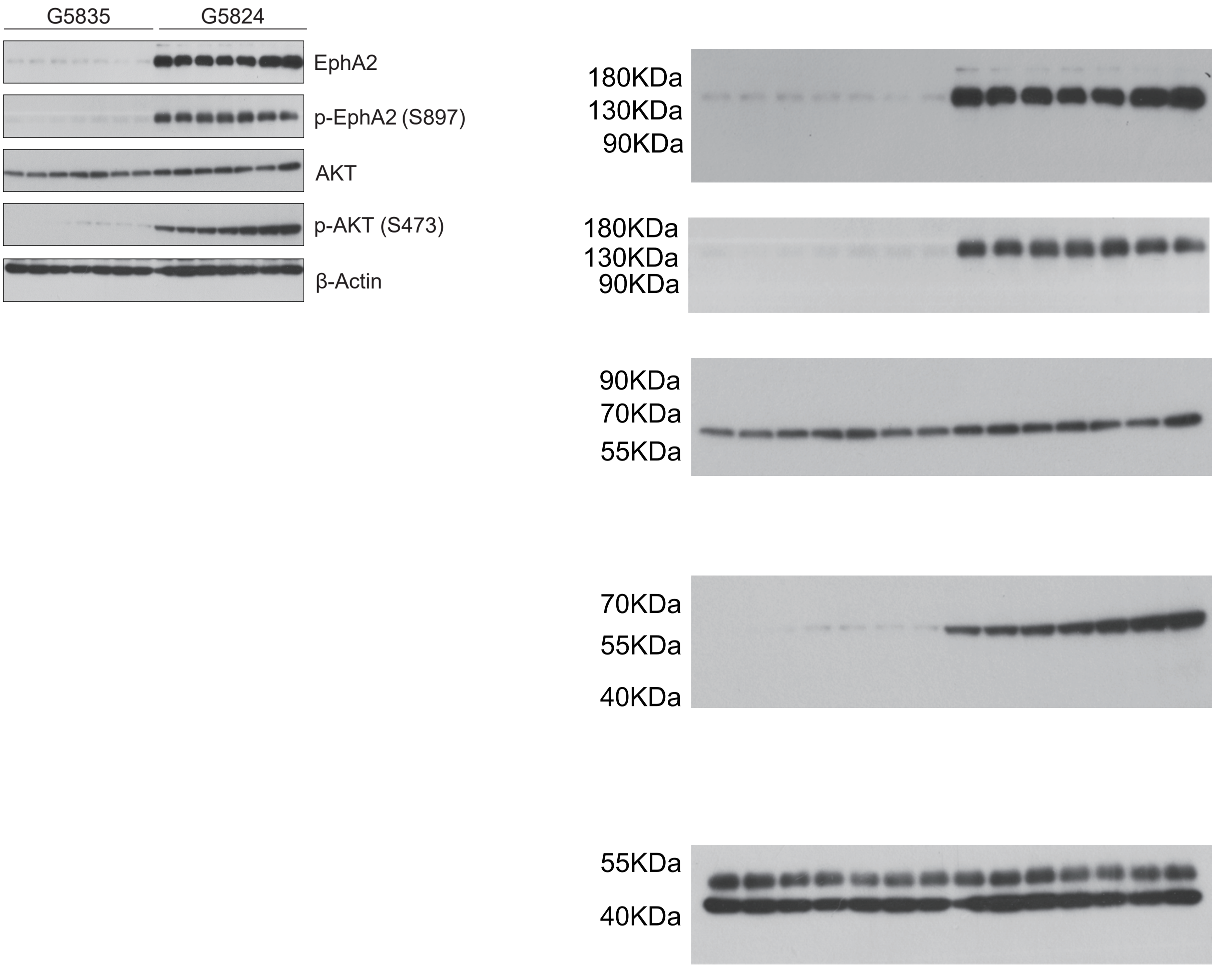
G5835
G5824
EphA2
p-EphA2 (S897)
AKT
p-AKT (S473)
β-Actin
180KDa
130KDa
90KDa
180KDa
130KDa
90KDa
90KDa
70KDa
55KDa
70KDa
55KDa
40KDa
55KDa
40KDa

Figure 4H

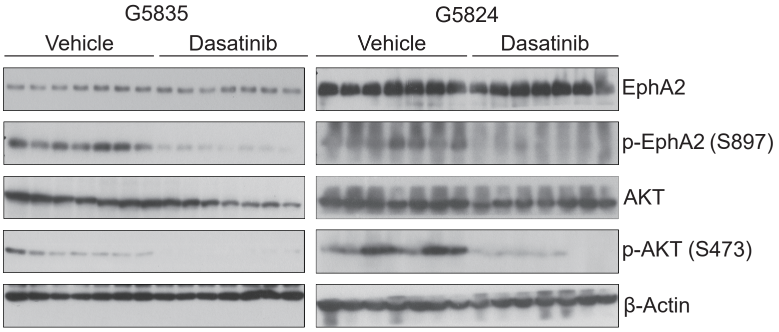
G5835
G5824
Vehicle
Dasatinib
Vehicle
Dasatinib
EphA2
p-EphA2 (S897)
AKT
p-AKT (S473)
β-Actin

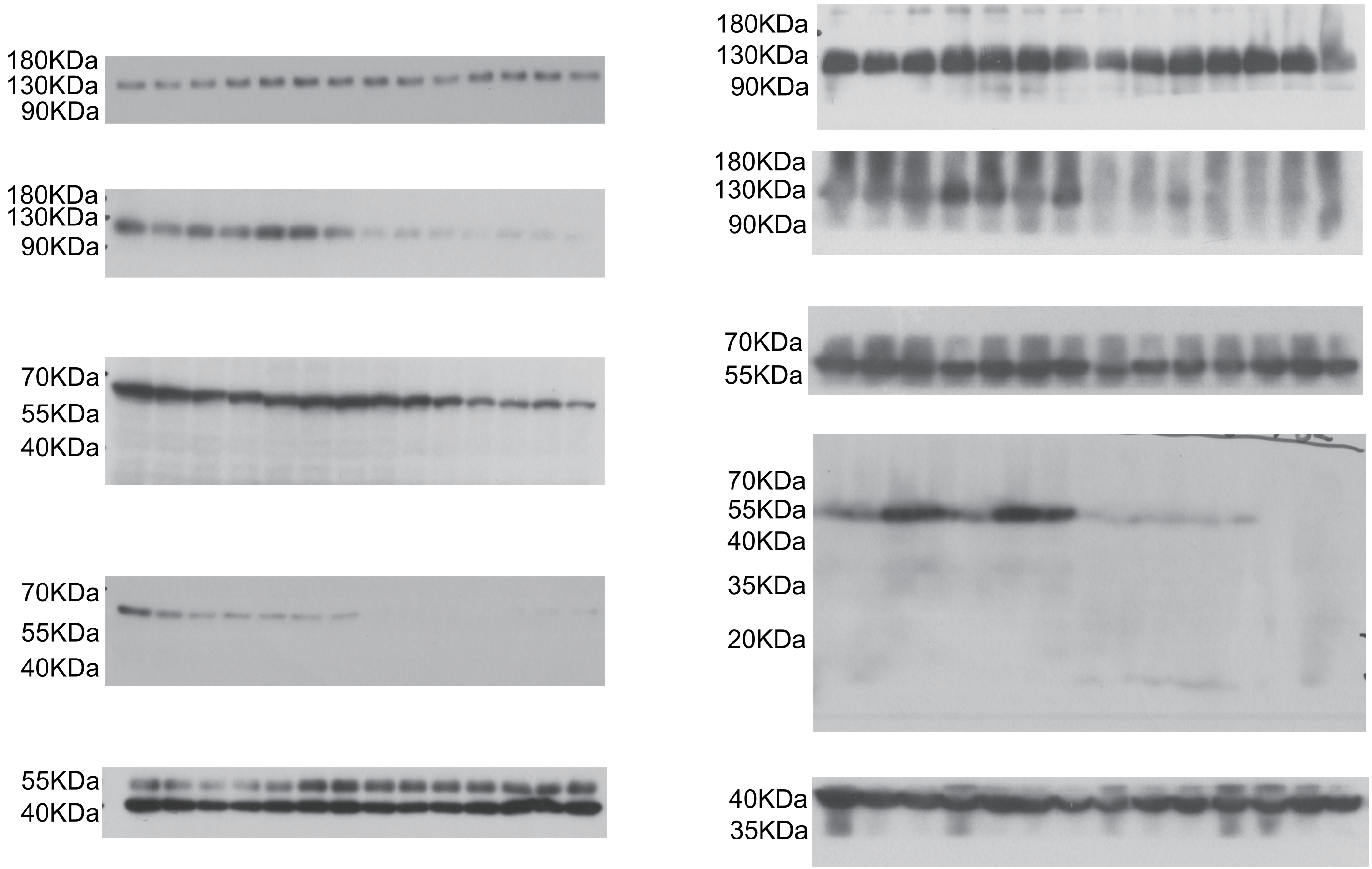
180KDa
130KDa
90KDa
180KDa
130KDa
90KDa
70KDa
55KDa
40KDa
70KDa
55KDa
40KDa
55KDa
40KDa
180KDa
130KDa
90KDa
180KDa
130KDa
90KDa
70KDa
55KDa
70KDa
55KDa
40KDa
35KDa
20KDa
40KDa
35KDa

Figure 6A

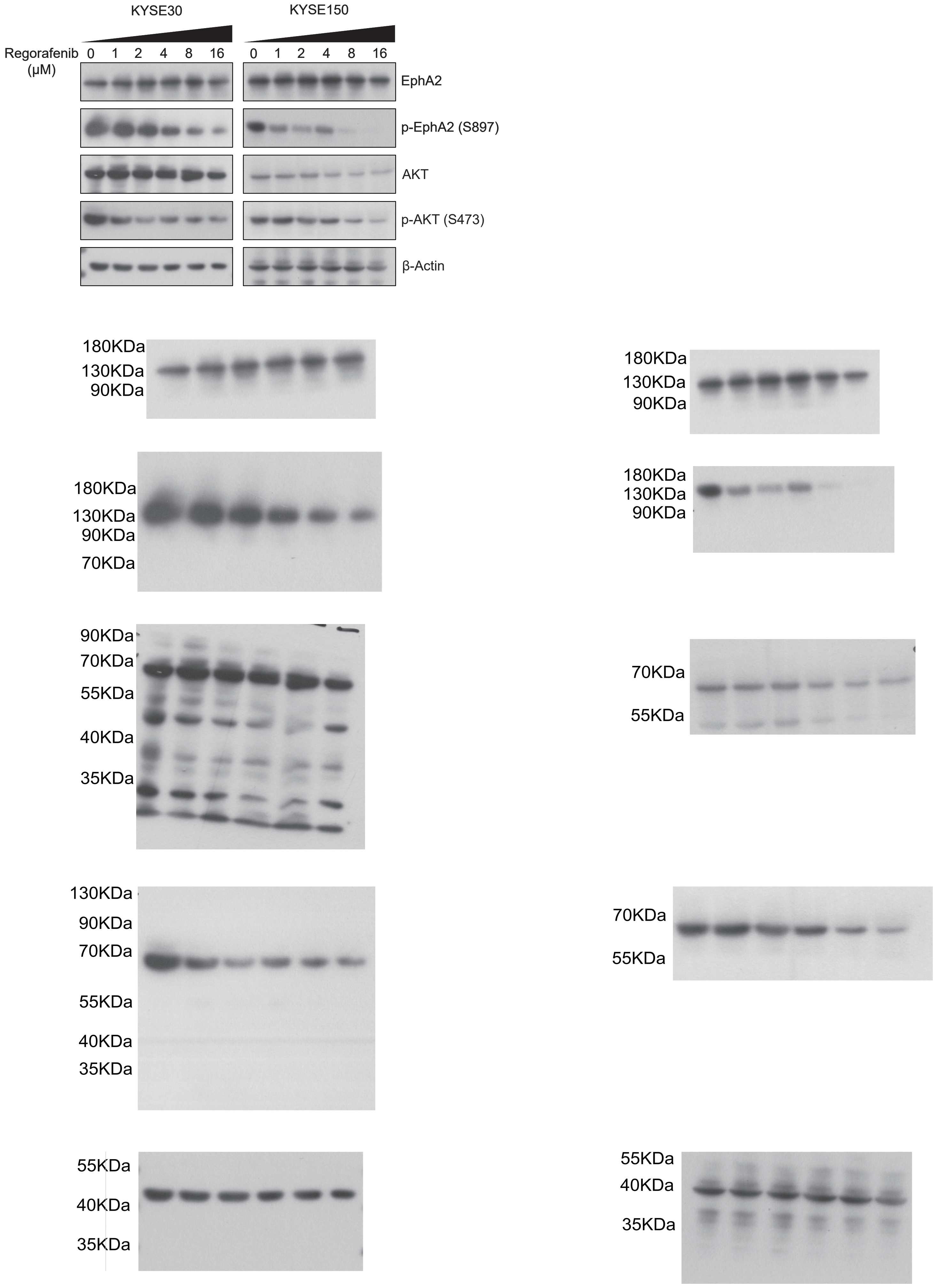
KYSE30
KYSE150
Regorafenib (μM)
0 1 2 4 8 16
0 1 2 4 8 16
EphA2
p-EphA2 (S897)
AKT
p-AKT (S473)
β-Actin
180KDa
130KDa
90KDa
70KDa
55KDa
40KDa
35KDa

Figure S4A

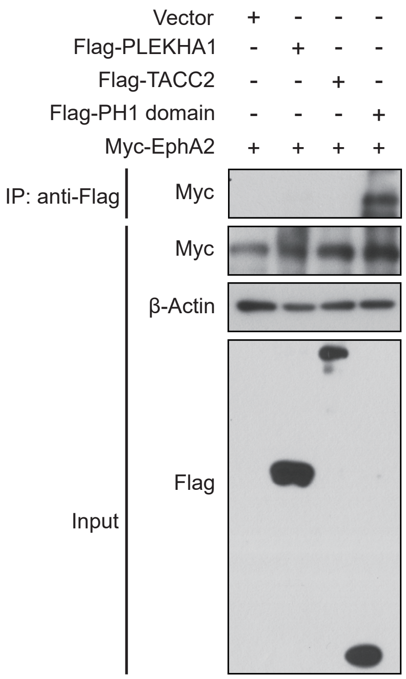

Vector + - - -
Flag-PLEKHA1 - + - -
Flag-TACC2 - - + -
Flag-PH1 domain - - - +
Myc-EphA2 + + + +
IP: anti-Flag
Myc
Myc
β-Actin
Flag
Input

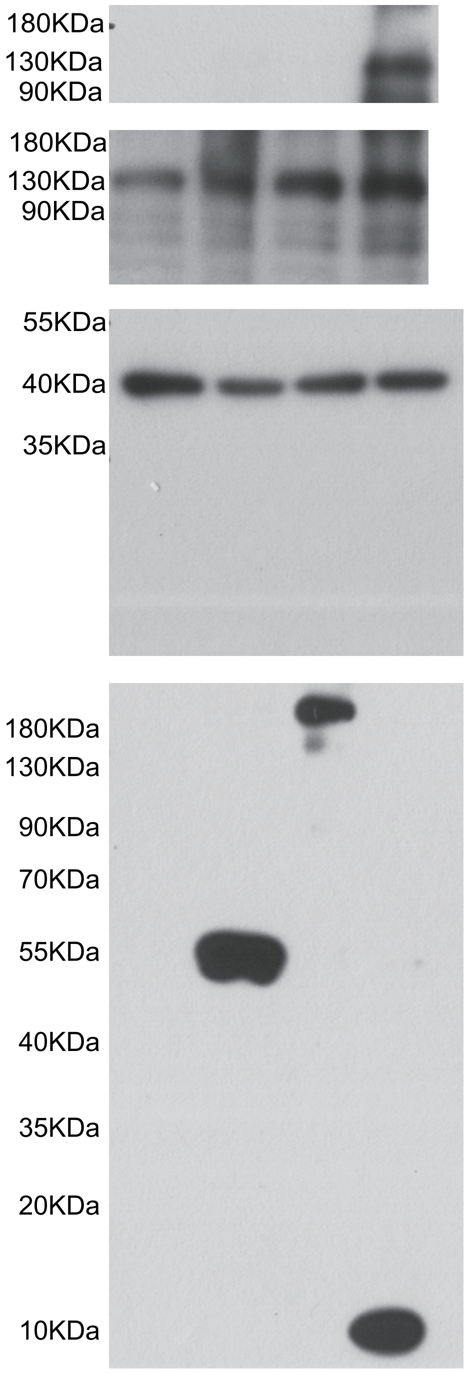

180KDa
130KDa
90KDa
180KDa
130KDa
90KDa
55KDa
40KDa
35KDa
180KDa
130KDa
90KDa
70KDa
55KDa
40KDa
35KDa
20KDa
10KDa

Figure S4B

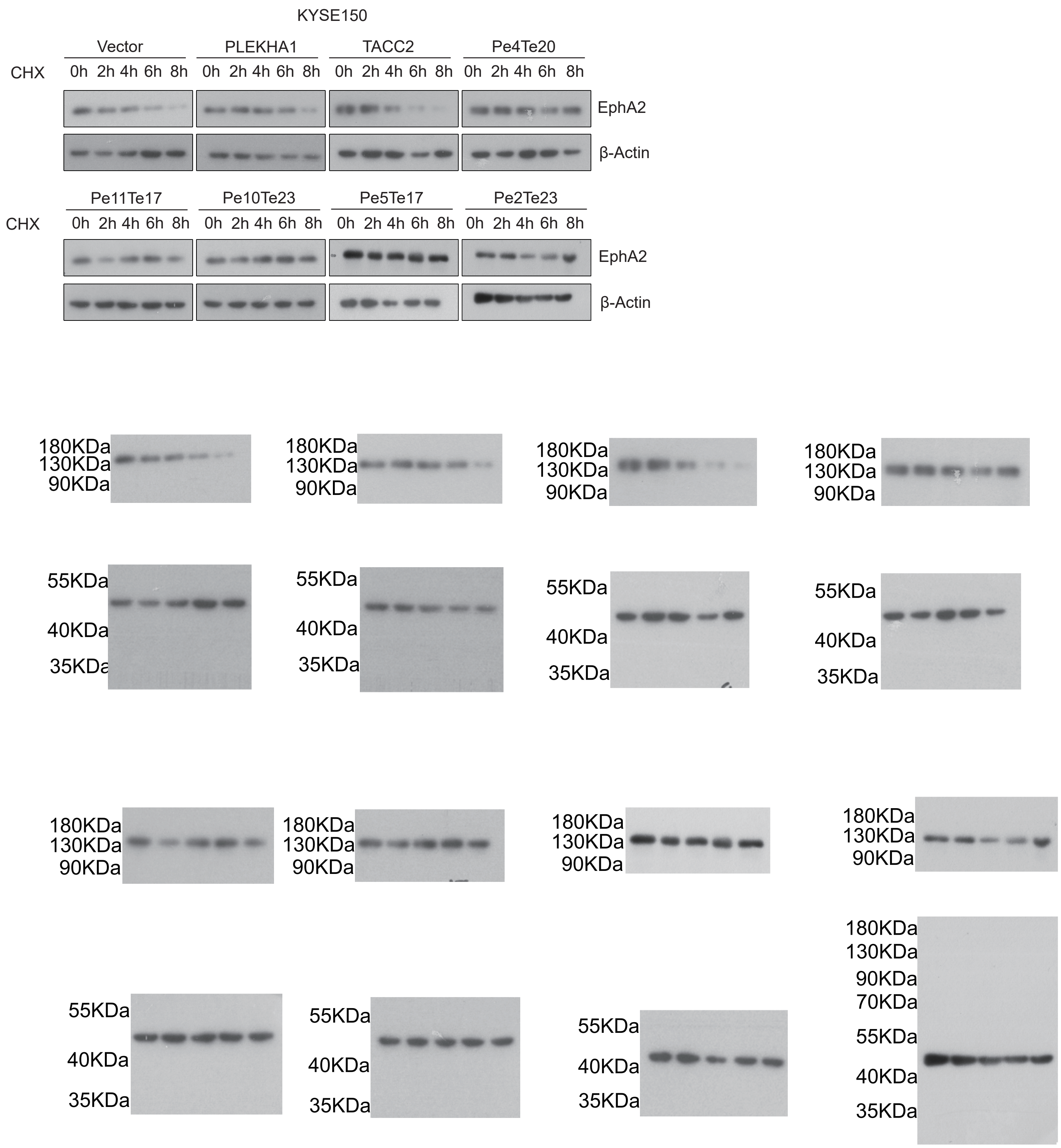
KYSE150
Vector
PLEKHA1
TACC2
Pe4Te20
CHX
0h 2h 4h 6h 8h
EphA2
β-Actin
Pe11Te17
Pe10Te23
Pe5Te17
Pe2Te23
180KDa
130KDa
90KDa
70KDa
55KDa
40KDa
35KDa

Figure S4C

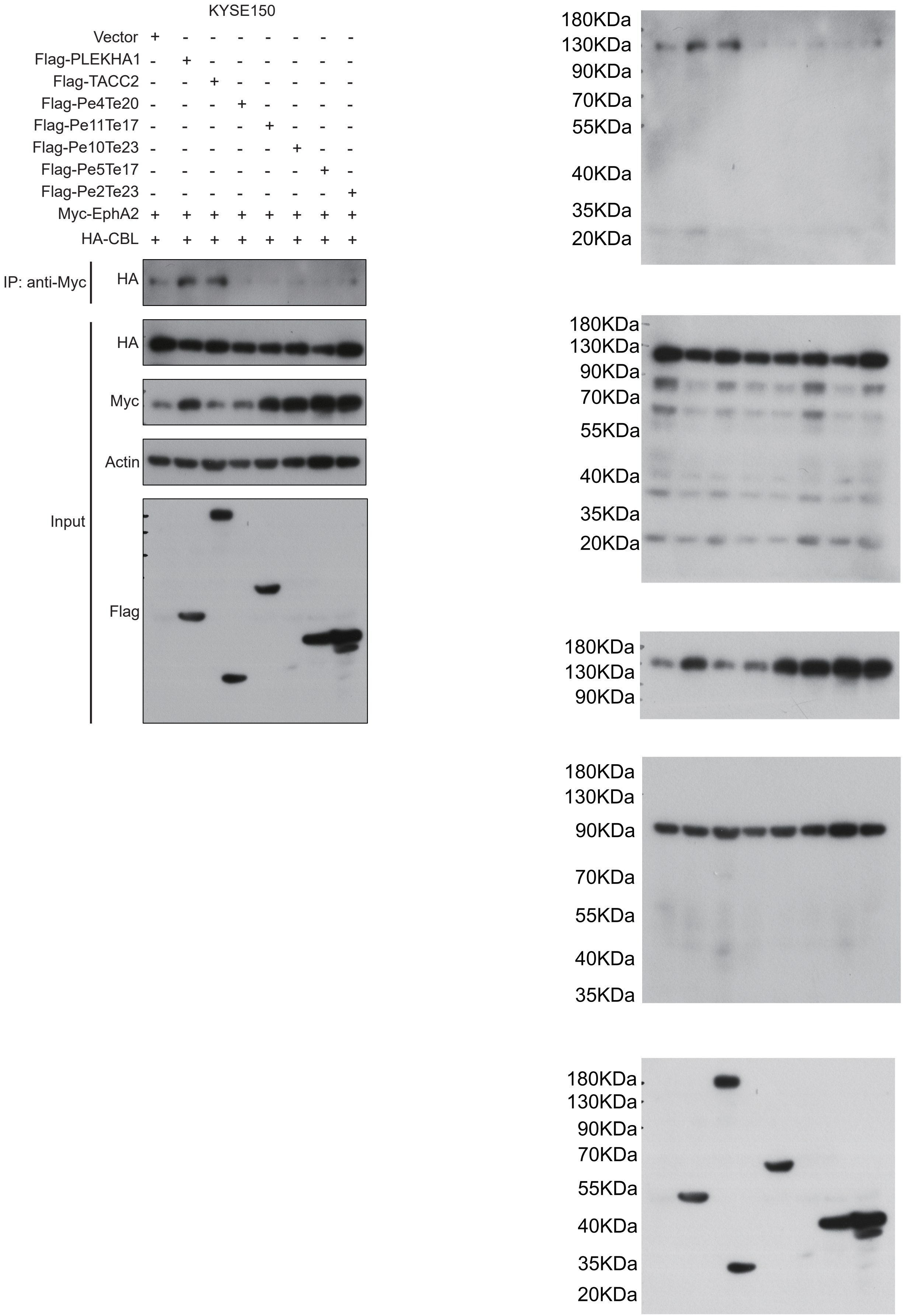
KYSE150
Vector + - - - - - - -
Flag-PLEKHA1 - + - - - - - -
Flag-TACC2 - - + - - - - -
Flag-Pe4Te20 - - - + - - - -
Flag-Pe11Te17 - - - - + - - -
Flag-Pe10Te23 - - - - - + - -
Flag-Pe5Te17 - - - - - - + -
Flag-Pe2Te23 - - - - - - - +
Myc-EphA2 + + + + + + + +
HA-CBL + + + + + + + +
IP: anti-Myc
HA
HA
Myc
Actin
Input
Flag
180KDa
130KDa
90KDa
70KDa
55KDa
40KDa
35KDa
20KDa
180KDa
130KDa
90KDa
70KDa
55KDa
40KDa
35KDa
20KDa
180KDa
130KDa
90KDa
180KDa
130KDa
90KDa
70KDa
55KDa
40KDa
35KDa
180KDa
130KDa
90KDa
70KDa
55KDa
40KDa
35KDa
20KDa

Figure S4D

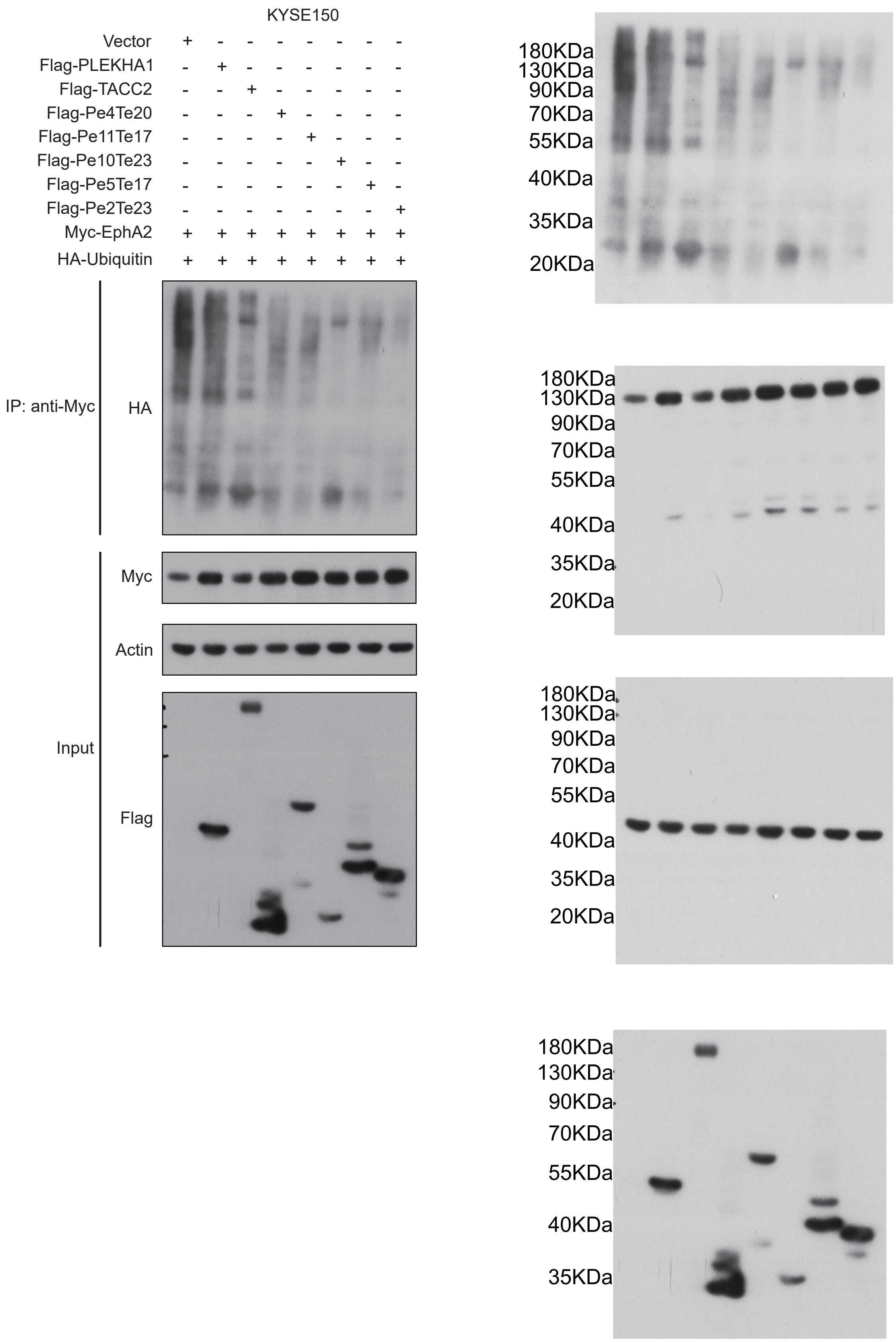
KYSE150
Vector + - - - - - - -
Flag-PLEKHA1 - + - - - - - -
Flag-TACC2 - - + - - - - -
Flag-Pe4Te20 - - - + - - - -
Flag-Pe11Te17 - - - - + - - -
Flag-Pe10Te23 - - - - - + - -
Flag-Pe5Te17 - - - - - - + -
Flag-Pe2Te23 - - - - - - - +
Myc-EphA2 + + + + + + + +
HA-Ubiquitin + + + + + + + +
IP: anti-Myc
HA
Myc
Actin
Input
Flag
180KDa
130KDa
90KDa
70KDa
55KDa
40KDa
35KDa
20KDa

Figure S5A

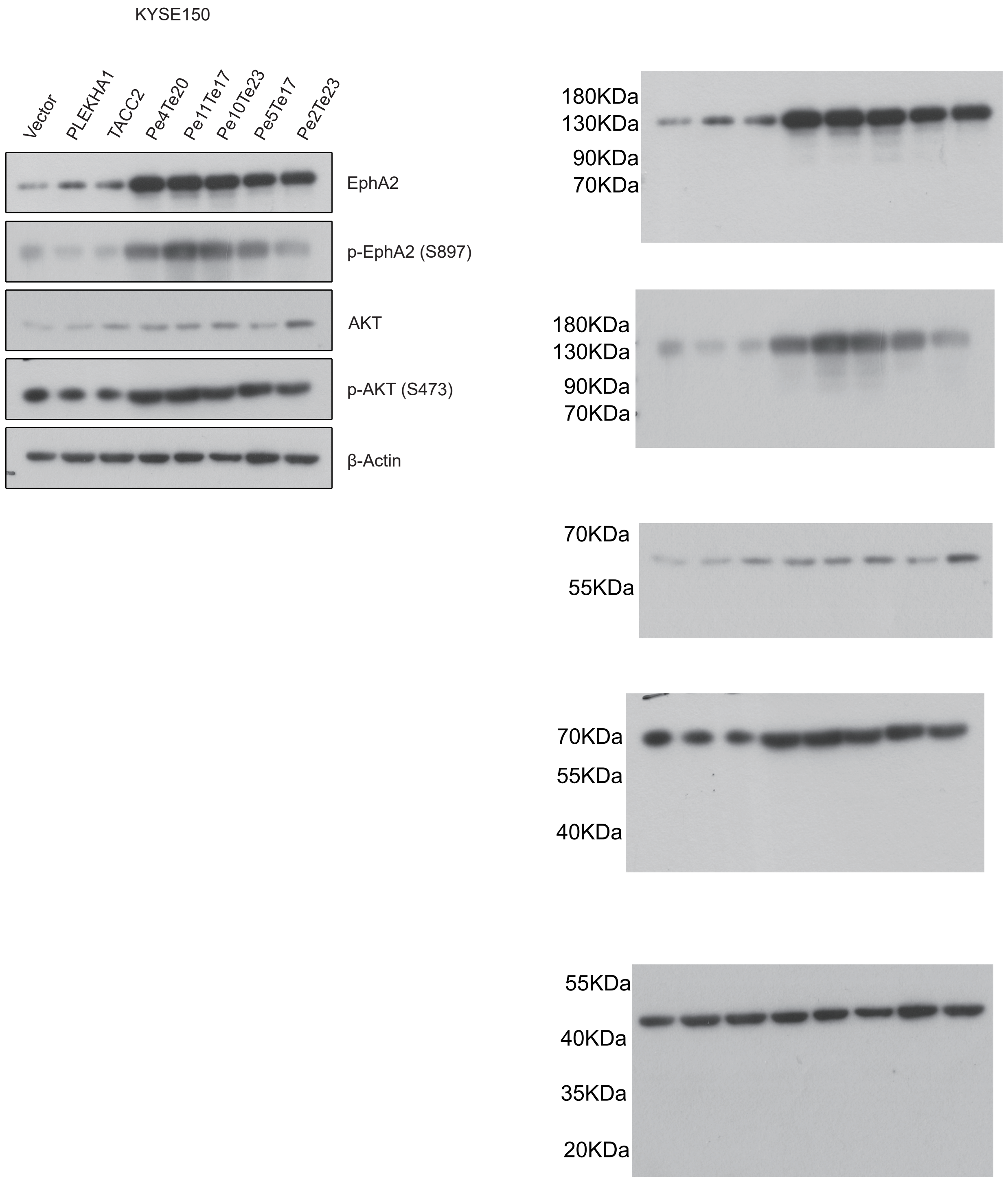
KYSE150
Vector
PLEKHA1
TACC2
Pe4Te20
Pe11Te17
Pe10Te23
Pe5Te17
Pe2Te23
EphA2
p-EphA2 (S897)
AKT
p-AKT (S473)
β-Actin
180KDa
130KDa
90KDa
70KDa
180KDa
130KDa
90KDa
70KDa
70KDa
55KDa
70KDa
55KDa
40KDa
55KDa
40KDa
35KDa
20KDa

Figure S5C

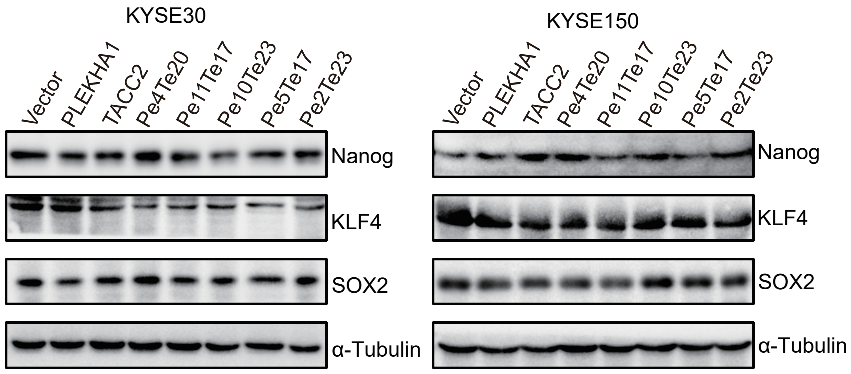
KYSE30
Vector
PLEKHA1
TACC2
Pe4Te20
Pe11Te17
Pe10Te23
Pe5Te17
Pe2Te23
Nanog
KLF4
SOX2
α-Tubulin
KYSE150
Vector
PLEKHA1
TACC2
Pe4Te20
Pe11Te17
Pe10Te23
Pe5Te17
Pe2Te23
Nanog
KLF4
SOX2
α-Tubulin

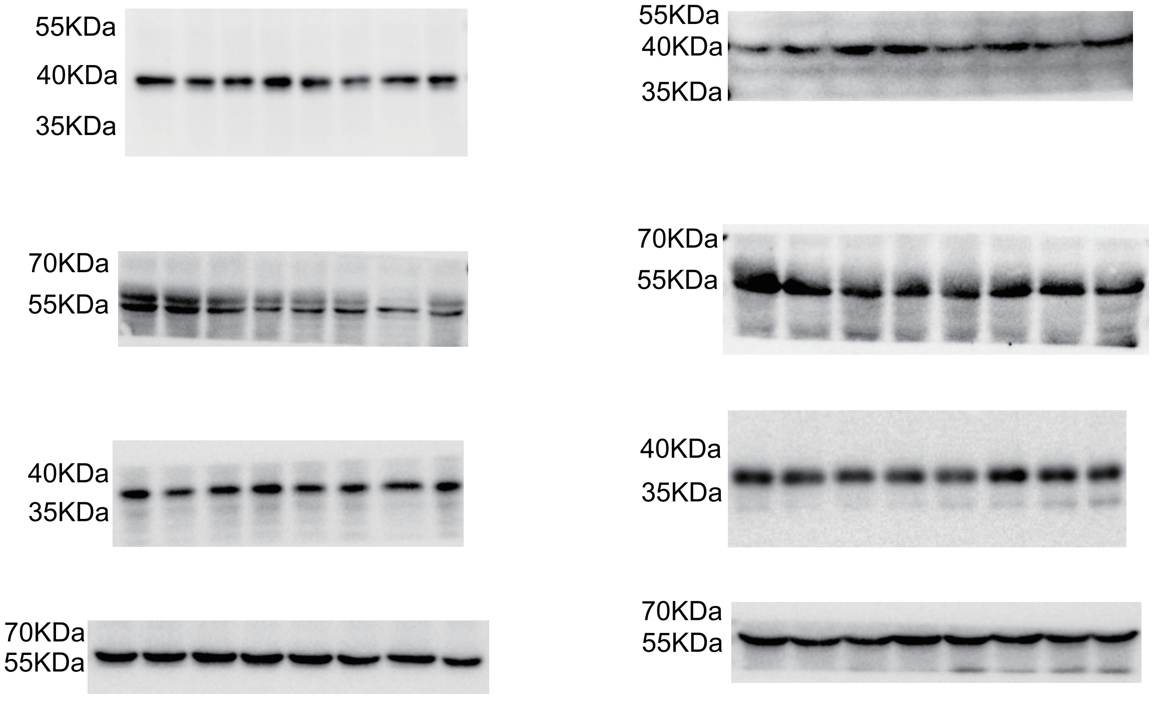
55KDa
40KDa
35KDa
55KDa
40KDa
35KDa
70KDa
55KDa
70KDa
55KDa
40KDa
35KDa
40KDa
35KDa
70KDa
55KDa
70KDa
55KDa

Figure S6B

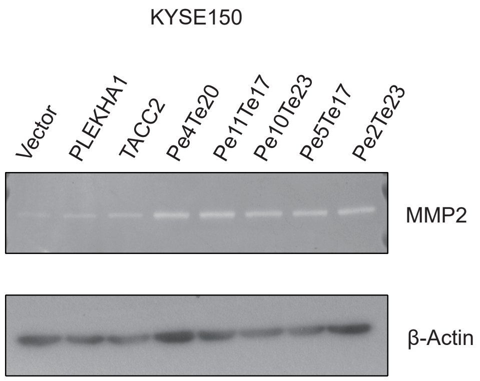

KYSE150
Vector
PLEKHA1
TACC2
Pe4Te20
Pe11Te17
Pe10Te23
Pe5Te17
Pe2Te23
MMP2
β-Actin

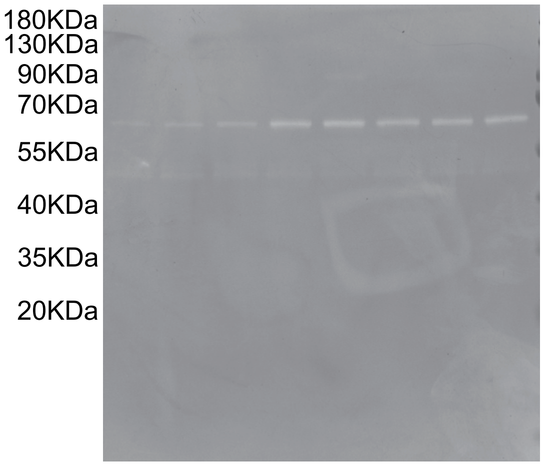

180KDa
130KDa
90KDa
70KDa
55KDa
40KDa
35KDa
20KDa

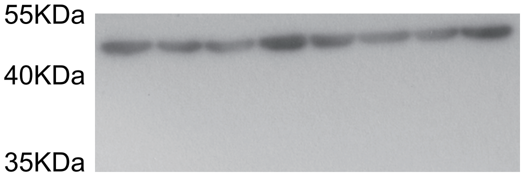

55KDa
40KDa
35KDa

Figure S9A

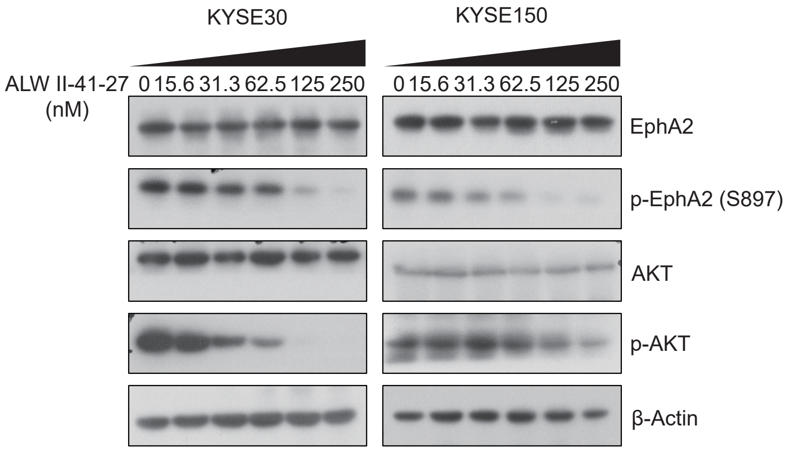
KYSE30
KYSE150
ALW II-41-27
(nM)
0 15.6 31.3 62.5 125 250
0 15.6 31.3 62.5 125 250
EphA2
p-EphA2 (S897)
AKT
p-AKT
β-Actin

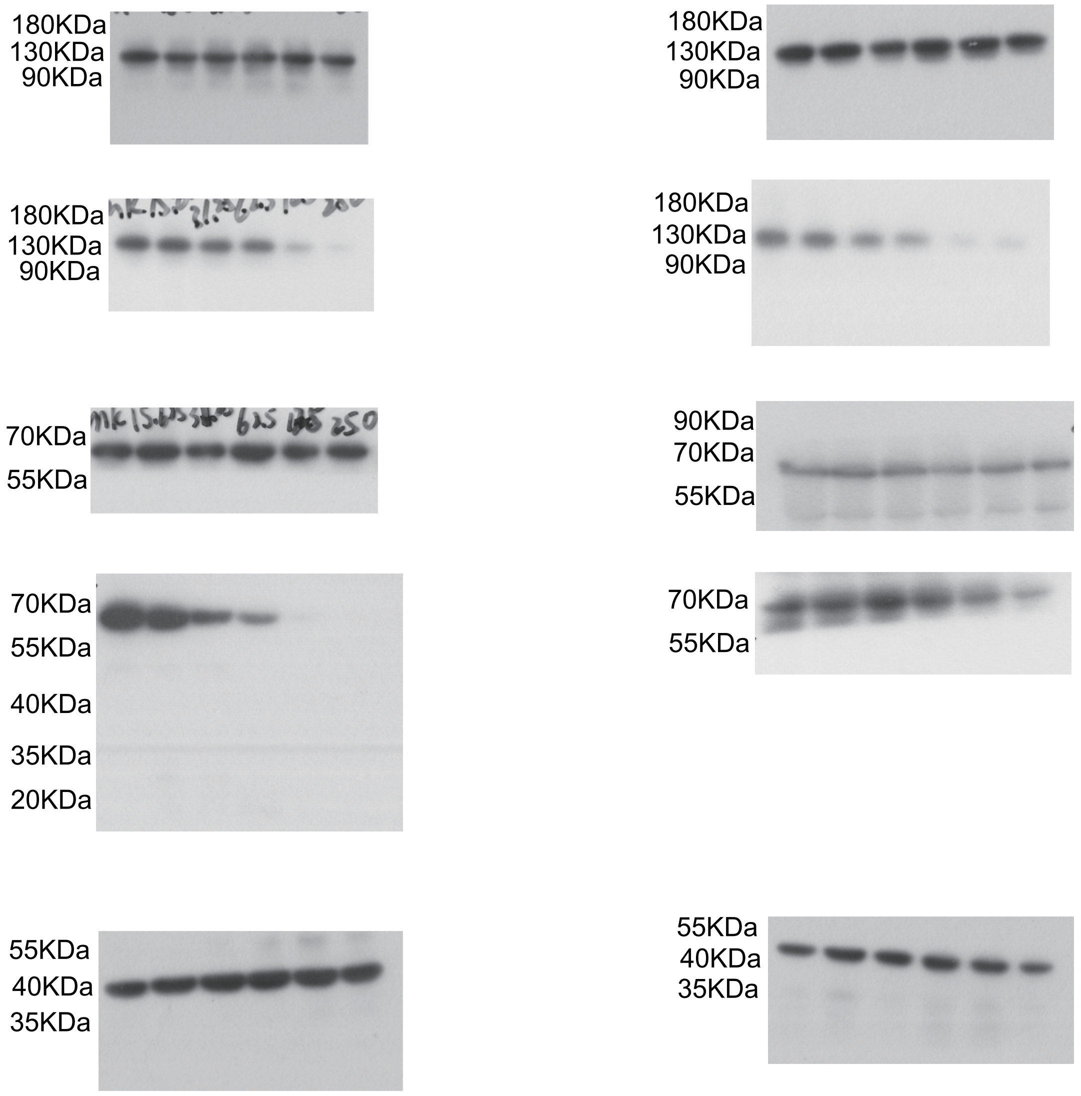
180KDa
130KDa
90KDa
180KDa
130KDa
90KDa
180KDa
130KDa
90KDa
180KDa
130KDa
90KDa
70KDa
55KDa
90KDa
70KDa
55KDa
70KDa
55KDa
40KDa
35KDa
20KDa
70KDa
55KDa
55KDa
40KDa
35KDa
55KDa
40KDa
35KDa

Figure S9G

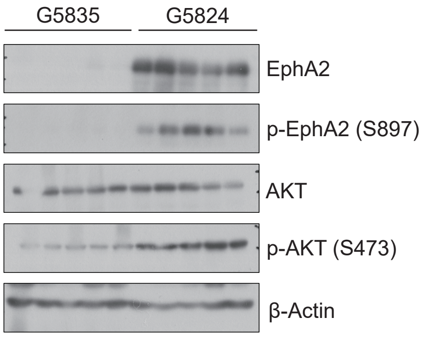
G5835
G5824
EphA2
p-EphA2 (S897)
AKT
p-AKT (S473)
β-Actin

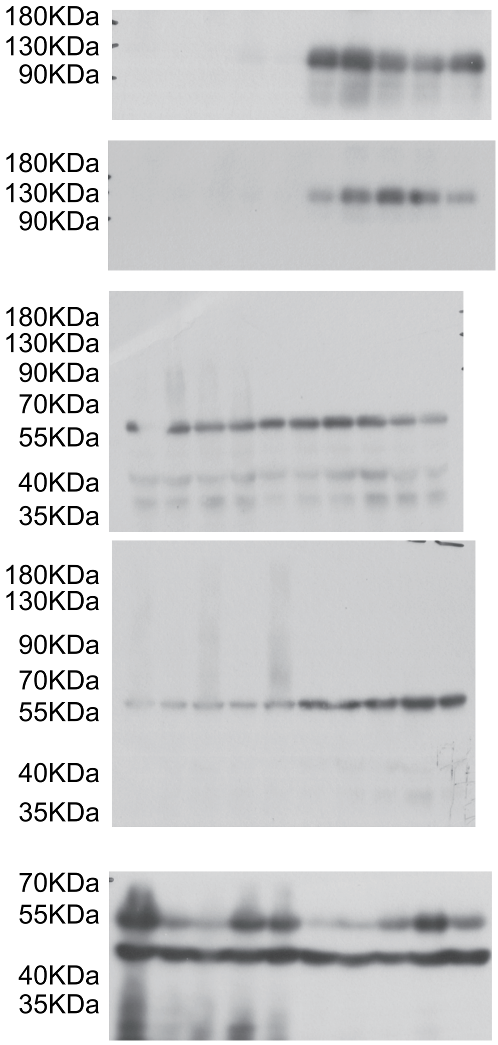
180KDa
130KDa
90KDa
180KDa
130KDa
90KDa
180KDa
130KDa
90KDa
70KDa
55KDa
40KDa
35KDa
180KDa
130KDa
90KDa
70KDa
55KDa
40KDa
35KDa
70KDa
55KDa
40KDa
35KDa

Figure S9H

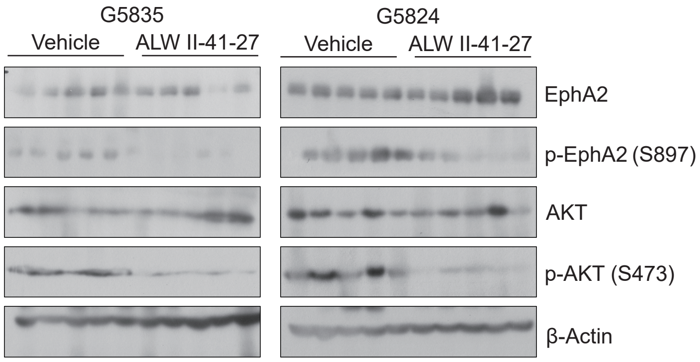
G5835
Vehicle
ALW II-41-27
G5824
Vehicle
ALW II-41-27
EphA2
p-EphA2 (S897)
AKT
p-AKT (S473)
β-Actin

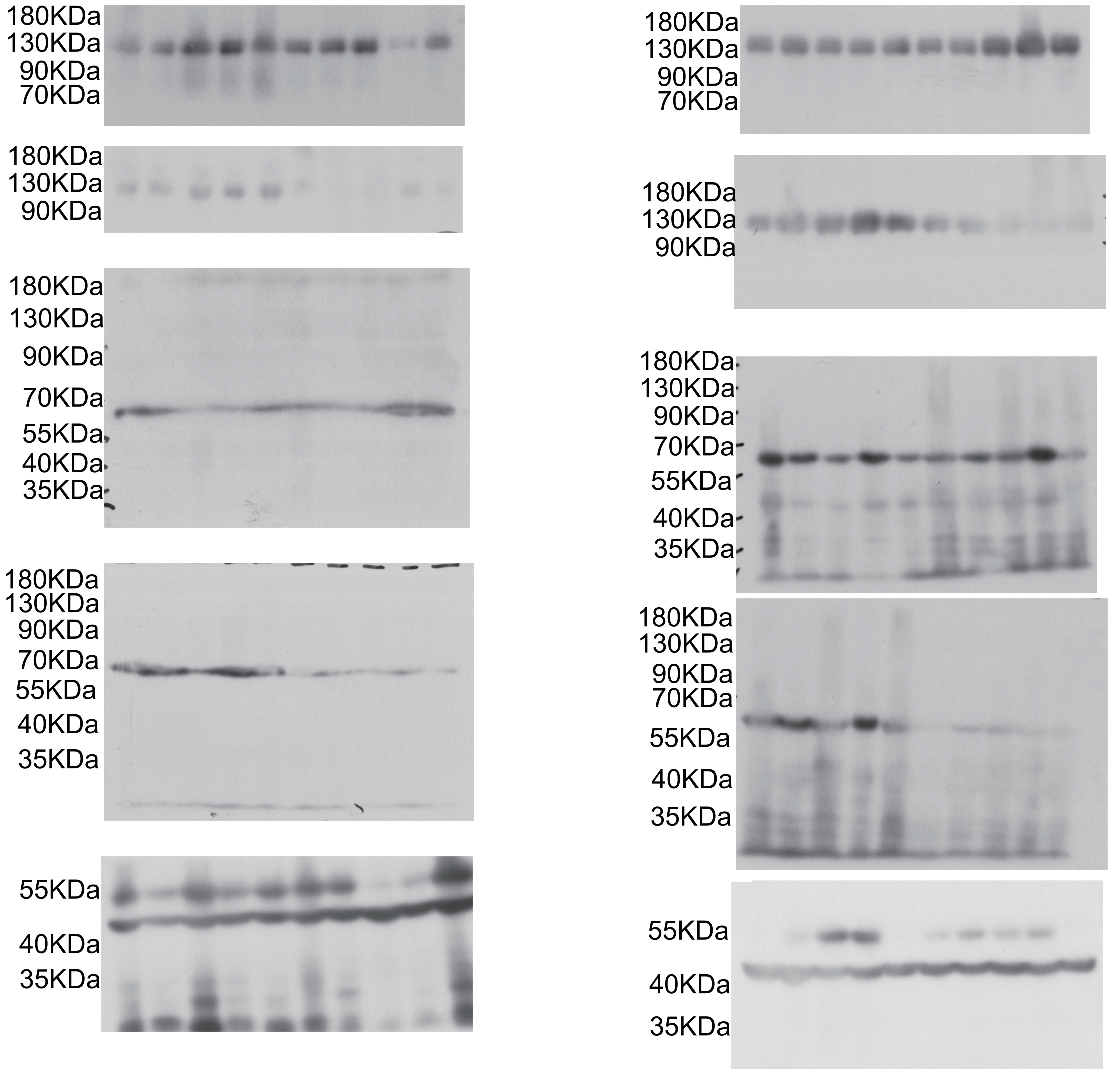
180KDa
130KDa
90KDa
70KDa
55KDa
40KDa
35KDa
